# Retarding efficiency loss upon scaling in perovskite photovoltaics as fill factor becomes the bottleneck

**DOI:** 10.1038/s41467-026-78867-1

**Published:** 2026-10-29

**Authors:** Buyi Yan, Ruihao Gong, Yanchun Huang, Nannan Gu, Runfeng Li, Dongxue Liu, Kangrong Yan, Xuehui Xu, Yang Michael Yang, Jizhong Yao, Dongchen Lan

**Affiliations:** 1 Microquanta Semiconductor Co., Ltd, Hangzhou, China; 2 College of Electrical Engineering, Zhejiang University, Hangzhou, China; 3 China Three Gorges Renewable (Group) Co., Ltd, Beijing, China; 4 State Key Laboratory of Modern Optical Instrumentation, Institute of Advanced Photonics, College of Optical Science and Engineering, Zhejiang University, Hangzhou, Zhejiang China; 5 Australian Centre for Advanced Photovoltaics, Sydney, Australia; 6 Zhejiang Provincial Key Laboratory of Optoelectronic Functional Materials and Devices, Hangzhou, China

**Keywords:** Solar energy and photovoltaic technology, Photovoltaics

## Abstract

Perovskite solar cells approach crystalline-silicon efficiencies at small areas, yet substantial performance losses during scale-up hinder their translation into practical modules. Here we quantify the contributions of device-performance parameters to efficiency loss during scale-up across diverse perovskite technologies, identifying fill factor (FF) as a major contributor and target for mitigating this loss. Further analysis resolves the *FF* deficit into voltage-, diode-non-ideality- and transport-associated contributions. Cryo-controlled processing provides an experimental route to preserve FF by regulating intermediate-phase evolution during crystallization. The resulting FF-preservation advantage arises predominantly from reduced diode non-ideality, with the other contributions being smaller. This advantage increases across device scales. A mini-module (19.48 cm^2^ aperture) achieves a record certified steady-state efficiency of 23.9% with an FF of 83.3%, the highest reported for perovskite modules. The same FF limitation governs operational performance decay, linking efficiency retention during scale-up with long-term stability. Together, these results establish FF preservation and the factors governing it as central to mitigating performance loss during scale-up and operation.

## Abstract (web summary)

Quantifying cell-to-module efficiency loss is critical for optimizing perovskite modules. Lan and colleagues quantify the contributions to this loss and establish fill factor preservation via cryo-controlled processing for mitigating scale-up loss. The resulting mini modules achieve a certified power conversion efficiency of 23.9%.

## Introduction

Perovskite solar cells (PSCs) have rapidly emerged as one of the most promising next-generation photovoltaic technologies owing to their high efficiency potential and low-cost manufacturing prospects, with certified power conversion efficiencies exceeding 28% in small-area devices^1^. Yet translating such performance to larger-area modules remains a central challenge for practical deployment^2–23^. Whereas the efficiency gap from record cells to modules is typically modest in crystalline silicon, perovskite photovoltaics still incur substantially larger losses during cell-to-module translation, leaving module performance well below that implied by high-performance laboratory-scale cells^1^. Resolving this translation gap is central to turning high-performance laboratory-scale perovskite cells into a scalable photovoltaic technology.

Numerous strategies have been developed to mitigate scaling-induced efficiency loss, including crystallization control^3,5–7,12,24–27^, interfacial engineering^4,8,10,11,28,29^ and module patterning optimization^5^. Although these approaches have steadily improved the performance of larger-area perovskite devices, they are frequently tailored to particular material systems, interfaces or processing routes, limiting their generality across device formats and manufacturing methods. More recently, figures of merit referenced to the Shockley-Queisser (SQ) limit^30^ have been used in cross-literature analyses to compare the evolution of losses associated with open-circuit voltage (${V}_{{oc}}$), short-circuit current density (${J}_{{sc}}$) and fill factor (FF) during scale-up across diverse fabrication routes, suggesting that FF generally represents the largest loss during upscaling^31^. Such analyses provide a broader picture of how ${V}_{{oc}}$-, ${J}_{{sc}}$- and FF-associated losses evolve with increasing device scale. Yet, a direct quantitative attribution of the measured CTM efficiency loss to ${V}_{{oc}}$, ${J}_{{sc}}$ and FF remains lacking. More importantly, understanding the origin of the pronounced FF loss during scale-up is essential for developing effective strategies for preserving FF and thereby mitigating the associated CTM efficiency loss.

Here, we quantify the respective contributions of ${V}_{{oc}}$, ${J}_{{sc}}$ and *FF* to the practical cell-to-module efficiency penalty, establishing *FF* as a major target for mitigating scale-up loss, and further resolving the factors underlying its preservation. To experimentally demonstrate this FF-preservation strategy, we employ cryo-controlled processing to regulate crystallization and preserve FF during scale-up^5,12,27^. Stepwise analysis reveals that the resulting FF preservation manifests predominantly through the contribution associated with the diode ideality factor (${n}_{{id}}$), with smaller contributions associated with the recombination-related voltage deficit and transport. Perovskite mini-modules consequently achieve a certified steady-state efficiency of 23.9% with an FF of 83.3%. The FF advantage is retained across the investigated device areas, helping to offset scale-up-induced efficiency loss. During operation, power conversion efficiency degradation closely follows FF degradation, whereas improved FF retention markedly slows performance decay. Together, these results demonstrate that preserving FF provides an effective strategy for maintaining photovoltaic performance during both device scale-up and long-term operation, offering practical guidance for efficient, stable and scalable perovskite photovoltaics.

## Results

### Efficiency loss during scale-up

**Fig. 1:**
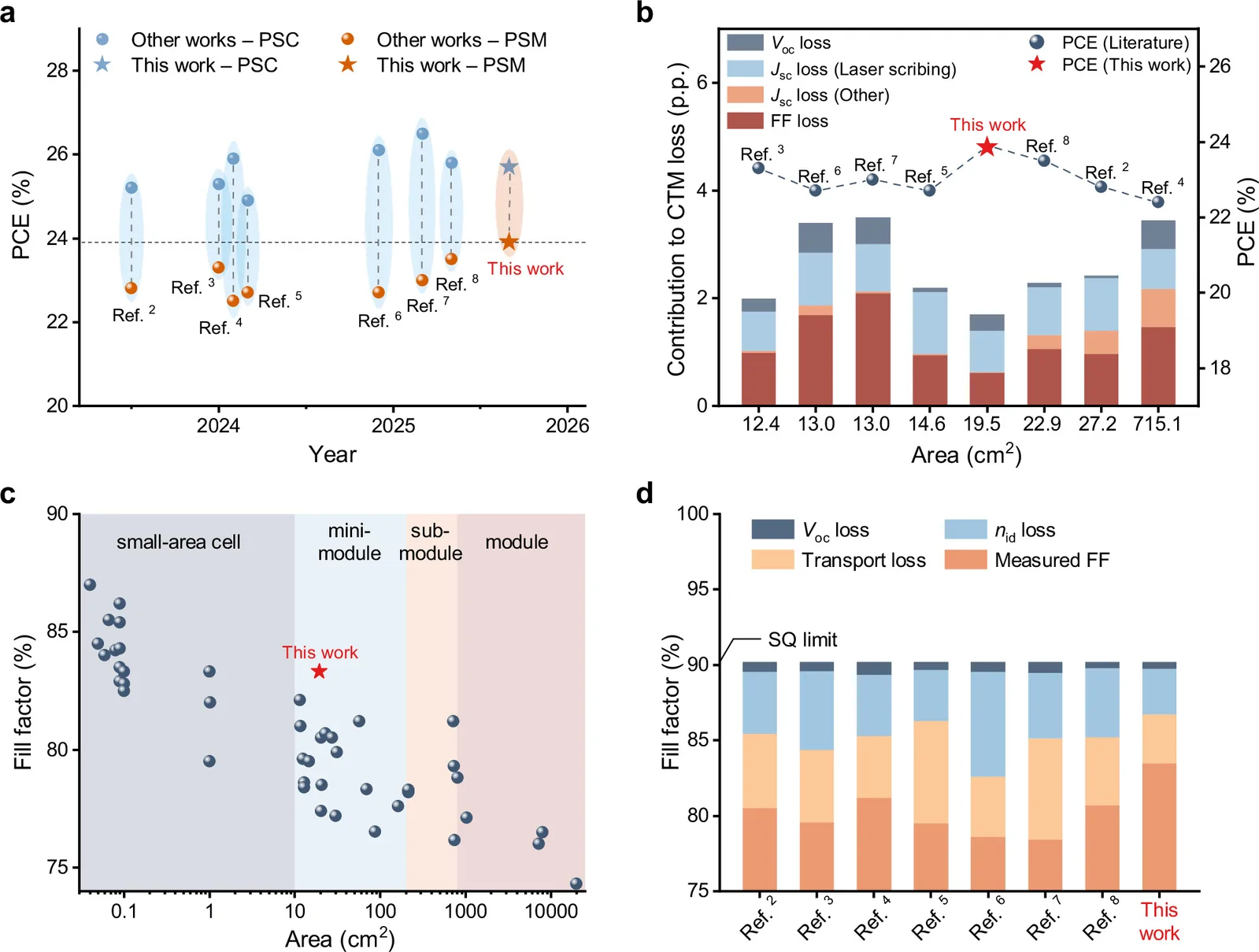
Scale-up loss analysis in perovskite photovoltaics. **a** Paired comparison of reported perovskite solar cells (PSCs) and their corresponding scaled-up perovskite solar modules (PSMs), showing the efficiency loss from cells to modules^2–8^. **b** Contributions of open-circuit voltage (${V}_{{oc}}$), short-circuit current density (${J}_{{sc}}$) and fill factor (FF) to the absolute cell-to-module (CTM) efficiency loss for PSMs of different aperture areas^2–8^, shown by the stacked bars in percentage points (p.p.) on the left axis, with the ${J}_{{sc}}$ contribution further separated into laser-scribing-related and residual losses. The circles and star indicate the corresponding module PCEs on the right axis. **c** FF as a function of device area for reported perovskite small-area cells, mini-modules, sub-modules and modules^1–8,10–12,25,42–52^; detailed data are provided in Supplementary Table 1. **d** Analysis of the FF deficit between the measured FF and its Shockley–Queisser limit into ${V}_{{oc}}$-related, ${n}_{{id}}$-associated and transport-associated contributions for reported mini-modules and this work^2–8^. The aligned upper boundary of each bar corresponds to the Shockley–Queisser FF limit^33^.

We first examined the efficiency gap between laboratory-scale PSCs and mini-modules across representative studies^2–8^ under matched processing conditions, to show the penalty incurred during scale-up (Fig. 1a). These paired cell–module comparisons reveal pronounced cell-to-module (CTM) efficiency losses despite the high efficiencies achieved in the corresponding small-area cells. To systematically understand these efficiency losses, we decomposed the absolute efficiency gap into contributions from ${V}_{{oc}}$, ${J}_{{sc}}$ and FF using a Shapley-value-based framework (Fig. 1b; methods described in Supplementary Note 1). Across these paired cell–module comparisons, FF emerges as the major contributor to CTM efficiency loss after excluding the ${J}_{{sc}}$ loss inherently associated with laser-scribing-induced inactive areas. Notably, the mini-modules developed in this work show the smallest CTM efficiency loss, largely owing to their reduced FF penalty (Fig. 1b and Supplementary Fig. 1), and consequently achieve the highest efficiency among modules with comparable aperture areas^1^.

More broadly, FF shows an overall decrease with increasing device area across diverse perovskite cells, mini-modules, sub-modules and modules (Fig. 1c), highlighting FF deterioration as a recurring challenge during scale-up in perovskite photovoltaics. Against this trend, the 19.48 cm^2^ device developed in this work retains an FF higher than many leading centimetre-scale devices, despite having an aperture area more than an order of magnitude larger. To quantify the parameter-level basis of this FF retention, we stepwise analysed the deficit between the measured FF and its theoretical upper limit, determined by Shockley–Queisser analysis, into ${V}_{{oc}}$-related, ${n}_{{id}}$-associated and transport-associated components (Fig. 1d; methods described in Supplementary Note 2)^32–35^. The ${V}_{{oc}}$-related component represents the FF loss associated with the recombination-related voltage deficit, whereas the ${n}_{{id}}$-associated component captures the FF loss due to diode non-ideality, potentially reflecting defect, interfacial, ionic and other effects^36–38^. The remaining transport-associated component accounts for FF losses represented by series and shunt resistances. The comparison indicates that both ${n}_{{id}}$-associated and transport-associated losses are reduced in this work relative to the literature benchmarks, contributing to the high FF achieved at module scale.

### Recombination-loss suppression

**Fig. 2:**
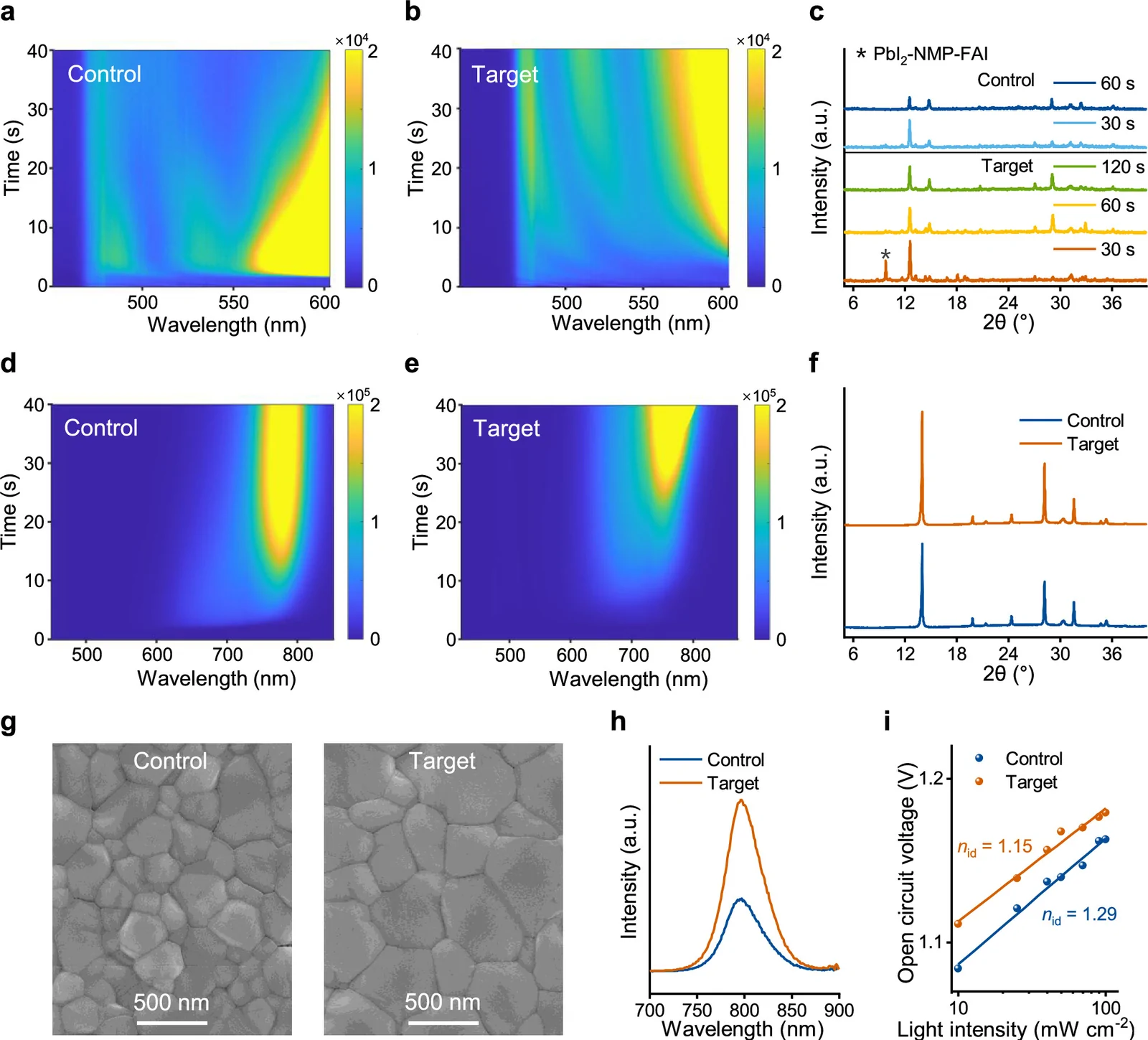
Crystallization control for recombination-loss suppression. **a**,** b** In situ photoluminescence (PL) spectra showing the evolution of intermediate phase-related PL signal during the initial vacuum-flash stage in control (**a**) and target perovskite films (**b**). **c** In situ XRD patterns recorded during vacuum-flash treatment of control and target films. The diffraction peak assigned to the PbI_2_-NMP-FAI intermediate is marked with an asterisk. **d**,** e** In situ PL evolution showing photoactive perovskite-phase formation in control (**d**) and target perovskite films (**e**). **f** XRD patterns of annealed control and target perovskite films. **g** SEM images of control and target perovskite films. Scale bar, 500 nm. **h** Steady-state PL spectra of control and target perovskite films. **i** Suns–${V}_{{oc}}$ measurements of control and target perovskite devices.

Recombination losses in perovskite photovoltaics are strongly influenced by the structural and optoelectronic quality of the perovskite film, which is established during crystallization^2,4,12,27,39^. Building on previous advances in low-temperature crystallization control^5,12,27^, we employed cryo-controlled processing to prolong intermediate-phase evolution by moderating solvent evaporation before vacuum flash (Supplementary Figs. 2 and 3). In situ photoluminescence (PL) and X-ray diffraction (XRD) were used to track phase evolution during film formation. Because solvent-coordinated precursor/intermediate complexes can give rise to transient PL features during wet-film formation^40^, we examined the 500–550 nm PL signal during the initial vacuum-flash stage. This PL signal rapidly decayed in the conventionally processed perovskite films (denoted as control films), whereas it persisted for more than twice as long in films processed with cryo-controlled crystallization (denoted as target films) (Fig. 2a, b). This delayed decay indicates that cryo-controlled crystallization prolongs the lifetime of precursor/intermediate species during early film formation. In situ XRD independently corroborated this effect. The diffraction feature assigned to the PbI_2_-NMP-FAI intermediate phase^40^ remained prominent in the target film at 30 s, whereas it was largely diminished in the control film at the same time (Fig. 2c). Its subsequent disappearance upon further drying indicates that cryo-controlled crystallization transiently retards intermediate-phase conversion rather than permanently trapping the intermediate. The formation of the photoactive perovskite phase was further tracked using in situ PL evolution (Fig. 2d, e). Compared with the abrupt emergence and rapid red-shift of the PL signal in the control films, the target films showed a delayed and more gradual PL evolution. This indicates that cryo-controlled crystallization slows intermediate-to-perovskite conversion during film formation.

XRD patterns showed sharper diffraction peaks in the target films than in the control films (Fig. 2f), while top-view SEM images revealed larger and more uniform grains (Fig. 2g), and cross-sectional SEM images showed larger grains extending through much of the perovskite-layer thickness (Supplementary Fig. 4), together indicating improved crystallinity and morphological quality. Steady-state PL and PL mapping were used to assess the optoelectronic quality of the annealed films. Steady-state PL intensity increased substantially after cryo-controlled crystallization, indicating reduced non-radiative recombination (Fig. 2h). PL mapping further revealed enhanced emission intensity and improved spatial uniformity across the film (Supplementary Fig. 5). Energy-dispersive X-ray spectroscopy (EDS) measurements at nine spatially distributed positions showed good compositional uniformity in both films (Supplementary Fig. 6). At the device level, EL imaging further revealed a more spatially uniform emission distribution across the target mini-module (Supplementary Fig. 7). Consistently, Suns–${V}_{{oc}}$ measurements showed an increase in ${V}_{{oc}}$ and a decrease in ${n}_{{id}}$ from 1.29 to 1.15 (Fig. 2i). The increase in ${V}_{{oc}}$ is consistent with reduced recombination at the device level, whereas the change in ${n}_{{id}}$ reflects altered diode non-ideality whose physical origin is not uniquely attributable to non-radiative recombination. Taken together, these results indicate that prolonged intermediate-phase evolution improves the crystallinity and morphological quality of the final perovskite films, thereby suppressing non-radiative recombination.

### Charge transport improvement

**Fig. 3:**
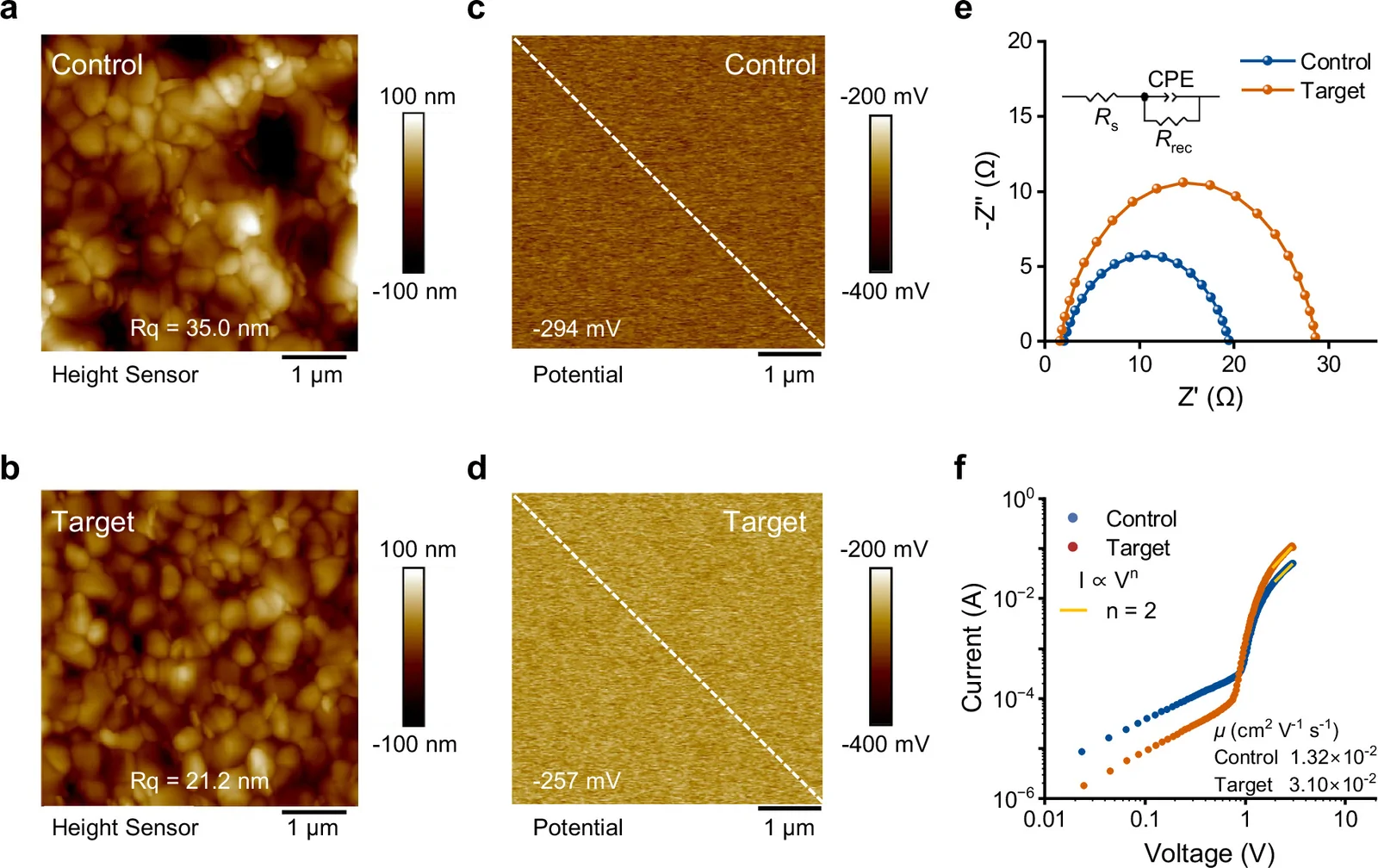
Charge-transport enhancement through crystallization control. **a**, **b** Atomic force microscopy (AFM) topography maps of control (**a**) and target (**b**) perovskite films. **c**, **d** Kelvin probe force microscopy (KPFM) surface-potential maps of control (**c**) and target (**d**) perovskite films. **e** Nyquist plots from electrochemical impedance spectroscopy (EIS) measurements of control and target devices. **f** Space-charge-limited current (SCLC) measurements of electron-only devices indicate enhanced electron mobility in the target device.

Cryo-controlled crystallization also altered film morphology and interfacial energetics, both of which influence charge transport. Cryo-controlled crystallization produced a smoother surface in the target film compared with the control, with the surface roughness decreasing from 35.0 to 21.2 nm (Fig. 3a, b). This improved morphology reduces interfacial inhomogeneity and provides a more uniform contact geometry, serving as a material-level basis for enhanced charge transport^41^. We then probed the corresponding interfacial energetics using Kelvin probe force microscopy (KPFM). The target film showed a higher surface potential than the control film (Fig. 3c, d and Supplementary Fig. 8), corresponding to a relative reduction in surface work function. This shift suggests an upward movement of the Fermi level, which may reduce the energetic offset at the perovskite/electron-transport-layer interface and facilitate electron transport. These observations suggest improved interfacial uniformity and more favourable energetics for charge extraction.

Device-level electrical measurements further show that cryo-controlled crystallization alleviates transport limitations while suppressing recombination. Electrochemical impedance spectroscopy (EIS), analysed by equivalent-circuit fitting of the Nyquist plots, revealed a lower series resistance and a higher recombination resistance in the target devices (Fig. 3e), indicating more efficient charge extraction and reduced recombination in complete devices. Consistently, space-charge-limited current (SCLC) measurements in electron-only devices showed that cryo-controlled crystallization increased the electron mobility by more than twofold (Fig. 3f), supporting faster carrier transport through the perovskite bulk. Taken together, these results indicate enhanced charge extraction and faster bulk transport, thereby reducing transport-related loss.

### Fill-factor preservation

**Fig. 4:**
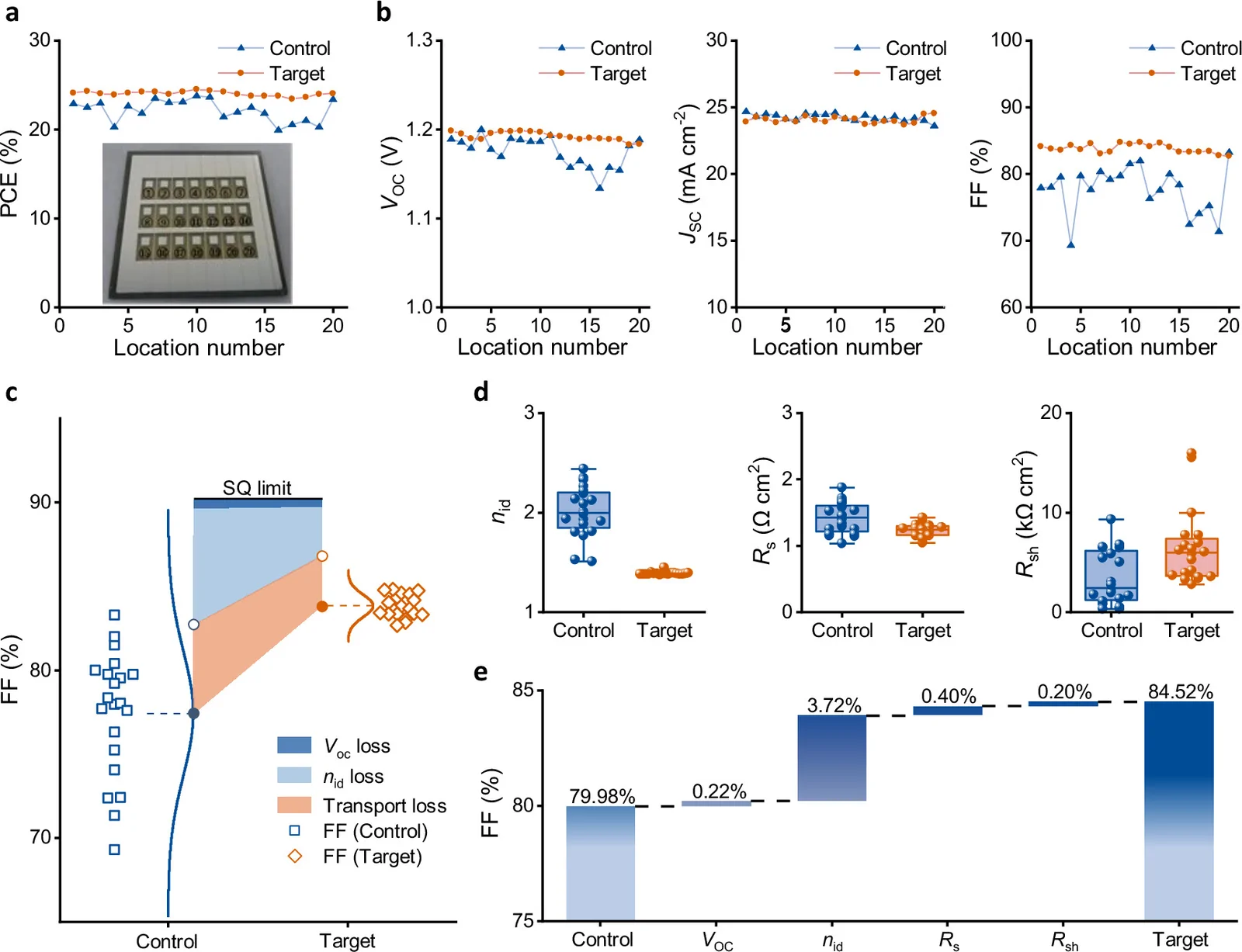
Fill-factor preservation at module scale. **a** PCE distribution across sampled regions of control and target mini-modules. **b** Spatial distribution of ${V}_{{oc}}$, ${J}_{{sc}}$ and FF across the mini-module for control and target devices. **c** Partitioning of the deficit between the measured FF and its SQ limit into ${V}_{{oc}}$-related, ${n}_{{id}}$-associated and transport-associated contributions for control and target mini-module regions. The solid and open circles denote the mean measured FF and the corresponding mean resistance-free FF calculated as described in Supplementary Note 2, respectively. Curves represent fitted normal distributions. **d** Ideality factor (${n}_{{id}}$), series resistance (${R}_{s}$) and shunt resistance (${R}_{{sh}}$) for control and target mini-modules. **e** Relative contributions of ${V}_{{oc}}$, ${n}_{{id}}$, ${R}_{s}$ and ${R}_{{sh}}$ to FF improvement. For **a**–**d**, twenty sampled regions were analysed for each control and target mini-module. Parameters shown in **d** and **e** were extracted from the corresponding regions analysed in **a**–**c** using single-diode-model analysis of the local current–voltage characteristics^53,54^.

The target mini-module exhibited higher and more uniform power conversion efficiency (PCE) than the control mini-module (Fig. 4a). The improvement was driven primarily by a higher and more spatially uniform FF, whereas the overall increase in ${V}_{{oc}}$ was smaller and ${J}_{{sc}}$ remained nearly unchanged across the sampled regions (Fig. 4b), consistent with the nearly identical absorption characteristics and closely matched EQE spectra of the control and target devices (Supplementary Figs. 9 and 10). Similar FF-centred performance improvements were observed in both mini-modules and larger-area modules (Supplementary Tables 2 and 3), further supporting that FF preservation plays a major role in mitigating performance loss upon scaling.

Analysis of the FF deficit relative to its theoretical upper bound revealed that the ${V}_{{oc}}$-related loss remained nearly unchanged, whereas the reduction in FF loss arose predominantly from the ${n}_{{id}}$-associated term, with a smaller contribution from the transport-associated term (Fig. 4c and Supplementary Note 2). The narrower distribution of measured FF in the target device further indicates reduced region-to-region variation across the mini-module. These changes are consistent with the improved optoelectronic and transport properties identified in the target films (Figs. 2 and 3).

The target devices exhibited lower ideality factor (${n}_{{id}}$), together with reduced series resistance (${R}_{s}$) and increased shunt resistance (${R}_{{sh}}$) (Fig. 4d). The narrower fitted-parameter distributions further indicate improved spatial uniformity across the target mini-module. Quantification of the individual parameter contributions further showed that the ${V}_{{oc}}$-, ${n}_{{id}}$-, ${R}_{s}$-, ${R}_{{sh}}$-associated terms contributed 0.22, 3.72, 0.40 and 0.20 percentage points, respectively, to the FF improvement (Fig. 4e). Thus, the FF improvement manifests predominantly as a reduction in the ${n}_{{{{\rm{id}}}}}$-associated loss, whereas the ${V}_{{oc}}$-related and transport-associated contributions are comparatively small.

### Scalability and stability

**Fig. 5:**
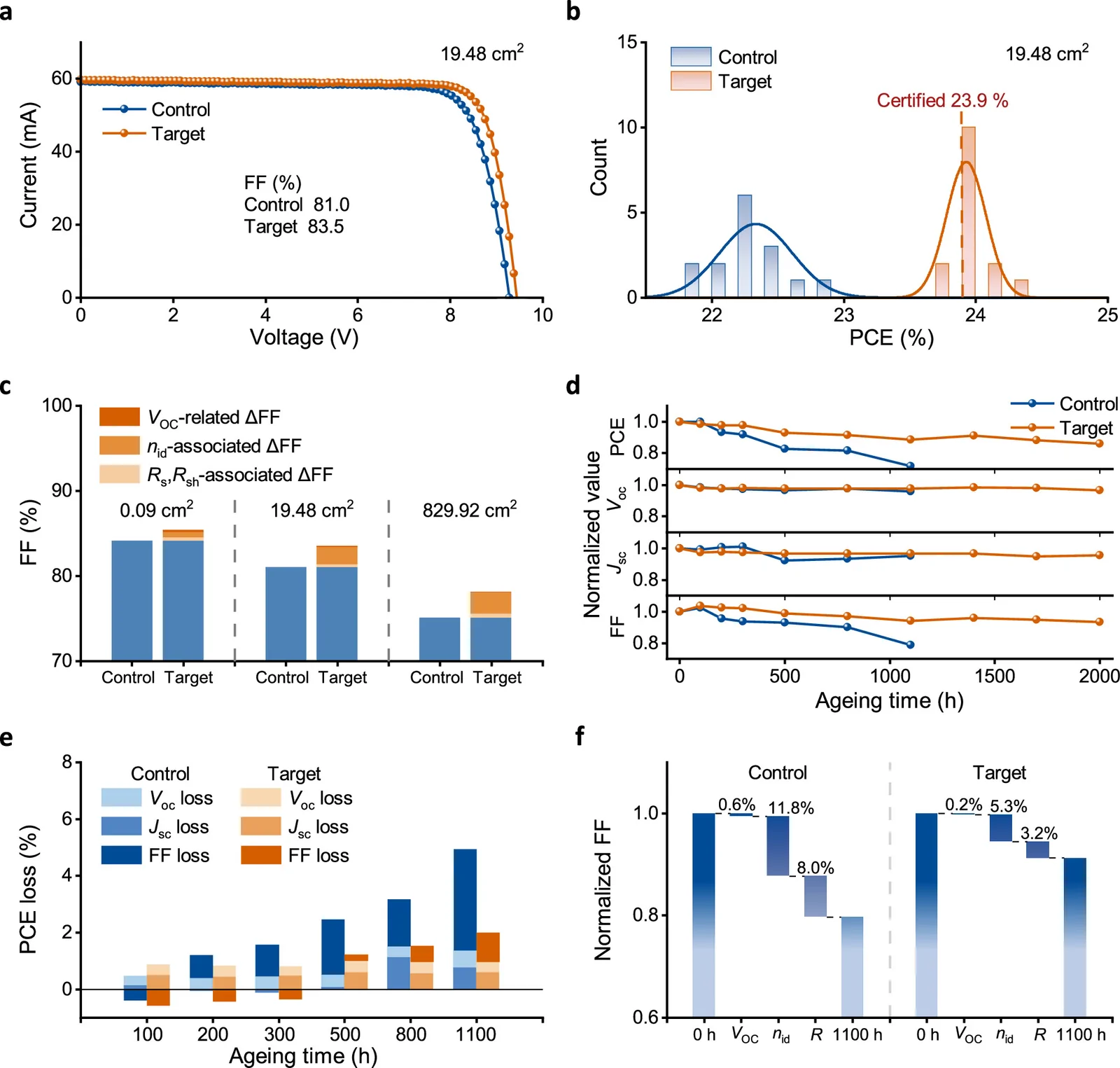
Performance preservation across area and ageing. **a** Current–voltage characteristics of 19.48 cm^2^ control and target mini-modules. **b** Efficiency distributions of 19.48 cm^2^ control and target mini-modules (N=15 per group). **c** Stepwise attribution of the FF improvements across device areas. Blue bars represent the measured FF of control cells, mini-modules and modules. Stacked increments indicate gains associated with ${V}_{{oc}}$, ${n}_{{{{\rm{id}}}}}$, ${R}_{s}$ and ${R}_{{{{\rm{sh}}}}}$; the total height represents the FF of the corresponding target devices. **d** Normalized evolution of PCE, ${V}_{{oc}}$, ${J}_{{sc}}$ and FF for control and target encapsulated modules during ageing under continuous open-circuit one-sun illumination. **e** Contributions of ${V}_{{oc}}$, ${J}_{{sc}}$ and FF degradation to efficiency degradation during ageing. **f** Stepwise analysis of the ageing-induced FF degradation into ${V}_{{{{\rm{oc}}}}}$-related, ${n}_{{{{\rm{id}}}}}$-associated and transport-associated contributions for control and target modules.

The target mini-modules exhibited not only higher efficiencies than the control mini-modules, but also a narrower efficiency distribution, indicating improved reproducibility (Fig. 5a, b). The certified steady-state efficiency of 23.9% places the target mini-module among the highest-performing module-level perovskite devices reported to date (Supplementary Fig. 11); notably, it attains an FF of 83.3%, approaching values reported for state-of-the-art crystalline-silicon modules despite the substantially larger scale than laboratory perovskite cells^1^.

The FF gain from control to target increased with device area, thereby offsetting scale-up-induced FF loss and improving FF preservation in the target devices (Fig. 5c). Consistently, higher efficiencies were maintained in the target devices from cells to modules (Fig. 5a, Supplementary Figs. 12–14 and Supplementary Tables 2–5; see Supplementary Fig. 15 for photographs of the mini-module and module). Stepwise attribution of the FF gain showed that the ${n}_{{{{\rm{id}}}}}$-associated contribution became increasingly dominant throughout scaling, whereas the ${V}_{{{{\rm{oc}}}}}$-related and transport-associated contributions remained small (Fig. 5c), consistent with the scale-dependent ${V}_{{oc}}$ and ${n}_{{id}}$ analysis in Supplementary Note 3. A comparison across device sizes further showed that the increase in ${n}_{{id}}$-associated loss constitutes the major contribution to FF degradation during scaling, whereas the increases in the ${V}_{{{{\rm{oc}}}}}$-related and transport-associated losses are comparatively small (Supplementary Note 4). This indicates that lateral current transport associated with module interconnection is not the primary origin of the scaling-induced FF degradation here. These findings show that the FF-preservation enabled by cryo-controlled crystallization remains effective as devices are further enlarged, manifesting predominantly as a reduced ${n}_{{id}}$-associated contribution, with improved charge transport providing an additional benefit.

During ageing, the PCE degradation largely tracked FF degradation in both control and target devices, with substantially smaller changes in ${J}_{{sc}}$ and ${V}_{{oc}}$ (Fig. 5d). Under continuous open-circuit one-sun illumination of encapsulated modules, FF was substantially better preserved in the target devices, accompanied by slower efficiency decay, whereas the control devices exhibited correspondingly larger FF losses and faster performance degradation. Partitioning the ageing-induced efficiency loss into ${V}_{{oc}}$-, ${J}_{{sc}}$- and FF-related contributions confirmed that FF loss dominated the efficiency decay, with the FF-related contribution reduced by more than threefold in the target devices compared with the controls (Fig. 5e). Further partitioning of FF degradation showed that the improved FF stability manifested predominantly as a smaller increase in the ${n}_{{id}}$-associated contribution, while transport-associated loss was also reduced (Fig. 5f; ${n}_{{id}}$-associated FF loss decreased by more than half, from 11.8 to 5.3 percentage points). This durability advantage was further supported under UV ageing, where the target devices remained above 80% of their initial efficiency until an accumulated UV dose of nearly 150 kWh, whereas the control devices crossed this threshold before 100 kWh (Supplementary Fig. 16). Together, these results show that FF preservation underpins both reduced scale-up loss and improved operational stability, with the reduction in ${n}_{{id}}$-associated loss providing the dominant contribution to FF preservation in the present cryo-controlled implementation.

## Discussion

We establish FF as a major target for reducing scale-up-induced efficiency loss and resolve the parameter-level basis of its preservation. Paired cell–module analysis quantifies the contributions of key performance parameters to the scale-up efficiency penalty, identifying *FF* as the largest recurring contribution across diverse perovskite technologies. Stepwise analysis then resolves the *FF-*preservation advantage predominantly into reduced ${n}_{{{{\rm{id}}}}}$-associated loss, with the other contributions being comparatively smaller. Cryo-controlled crystallization provides an experimental route to achieve this FF preservation by prolonging intermediate-phase evolution and improving film uniformity. The resulting *FF* advantage is retained across device scales and during operation, revealing a shared parameter-level pattern between scale-up loss and operational decay. Hence, preserving *FF* offers a unifying optimization target for developing efficient, stable and scalable perovskite photovoltaics.

## Methods

### Resource availability

#### Lead contact

Further information should be directed to and will be fulfilled by the Lead Contact, Dongchen Lan (d.lan@zju.edu.cn).

### Materials

Nickel acetylacetonate dihydrate (Ni(acac)_2_·2H_2_O, 99%) was purchased from Shanghai Aladdin. Ethanol (99.7%), ethanolamine (99.5%), N,N-dimethylformamide (DMF, 99.5%) and 1-methyl-2-pyrrolidinone (NMP, 99.5%) were obtained from J&K Scientific. Tetramethylene sulfoxide (TMSO, 98%), 3PATAT-C3 (98%), lead iodide (PbI_2_, 99.0%), formamidinium iodide (FAI, 98.0%), and lead chloride (PbCl_2_, 99%) were purchased from TCI Chemicals. Cesium iodide (CsI, 99.9%), methylammonium chloride (MACl, 98%) and ethylenediamine diiodide (EDAI_2_, 98%) were purchased from Sigma–Aldrich. C_60_ (99.5%) was purchased from Henan Fullerene Nano New Material Technology. Bathocuproine (BCP, 99%) was obtained from ChasingLight Technology. All chemicals were used as received without further purification.

### Solution preparation

#### NiO_x_ precursor solution

$1{{{\rm{mmol}}}}$ of nickel acetylacetonate dihydrate (Ni(acac)_2_·2H_2_O) was dissolved in $10\,{{\mathrm{mL}}}$ of ethanol and $50\,{{{\rm{\mu }}}}{{{\rm{L}}}}$ of ethanolamine under continuous mechanical stirring for $4\,{{{\rm{h}}}}$ to form a homogeneous solution. The solution was filtered through a 0.22-μm PTFE filter before use. The preparation process was carried out in air with a relative humidity below 45%.

#### 3PATAT-C3 solution

3PATAT-C3 was mixed with DMF to form a 0.25 mg mL^−1^ solution. The solution was prepared in 10-mL batches every $12\,{{{\rm{h}}}}$ and filtered through a 0.22-μm PTFE filter before use. All preparation steps were carried out in a nitrogen‑filled glovebox.

#### Perovskite precursor solution

The composition of FA_0.95_Cs_0.05_PbI_3_ was adopted for this study. First, $6.915{{{\rm{g}}}}$ PbI_2_, $2.451{{{\rm{g}}}}$ FAI, and $0.195{{{\rm{g}}}}$ CsI were dissolved in 10 mL of a mixed solvent of DMF:NMP:TMSO (15:4:1 by volume) and stirred mechanically for 2 h. Subsequently, an excess of 10 mol% of MACl and 3 mol% of PbCl_2_ was added to the perovskite precursor solution. The solution was prepared in a nitrogen‑filled glovebox and filtered through a 0.22-μm PTFE filter before use.

### Device fabrication

#### Solar-cell fabrication

ITO-coated glass substrates (2.5 × 2.5 cm) were sequentially ultrasonicated in diluted detergent, deionized water, isopropanol and ethanol for 15 min each, then dried under a nitrogen stream and treated with UV-ozone for 10 min before deposition of hole-transport layers (HTLs). The NiO_x_ precursor was spin-coated on the as-cleaned ITO substrates and then annealed at 350 °C for 10 min. The 3PATAT-C3 layer was deposited by spin-coating at 4000 rpm for 30 s (ramp rate, 2000 rpm s^−1^) in a nitrogen-filled glovebox, followed by annealing at 100 °C for 2 min. The perovskite precursor solution was spin-coated onto the substrates using a two-step program: first at 1000 rpm for $10{{{\rm{s}}}}$ (ramp rate, $1000\,{{\mathrm{rpm}}}{{{{\rm{s}}}}}^{-1}$), and then at 5000 rpm for 40 s (ramp rate, $2000\,{{\mathrm{rpm}}}{{{{\rm{s}}}}}^{-1}$) in air with 15–20% relative humidity. Immediately after spin-coating, the wet perovskite films were cooled for 15 s, reaching an experimentally measured film temperature of 0–10 °C immediately before vacuum treatment (Supplementary Fig. 17). The films were then transferred to the vacuum-flash chamber within approximately 5 s. Vacuum flashing was performed in a chamber with an effective internal volume of approximately $69.6\,{{{{\rm{cm}}}}}^{3}$ for a total treatment time of 120 s. The chamber pressure was initially approximately $1.0\times {10}^{5}\,{{{\rm{Pa}}}}$ and decreased to below 10 Pa within approximately 8 s. During the remaining approximately 112 s, the pressure gradually decreased from approximately 10 Pa to approximately 5 Pa, reaching a final pressure of approximately 5 Pa (Supplementary Fig. 18). The films were then annealed at 110 °C for 30 min in air. After cooling to room temperature, a solution of EDAI_2_ in isopropanol ($0.5\,{{\mathrm{mg}}}\,{{{\mathrm{mL}}}}^{-1}$) was spin-coated onto the perovskite films at $5,000{{{\rm{rpm}}}}$ and annealed at 85 °C for $1\,\min$. Finally, $20\,{{\mathrm{nm}}}$ of C_60_, $5\,{{\mathrm{nm}}}$ of BCP and $120\,{{\mathrm{nm}}}$ of Ag were sequentially deposited onto the perovskite films by thermal evaporation.

#### Module fabrication

The perovskite mini-modules used an inverted architecture with a layer-by-layer structure of ITO/NiO_x_/3PATAT-C3/perovskite (FA_0.95_Cs_0.05_PbI_3_) /C_60_/BCP/Ag. First, a 532-nm laser was used to perform P1 scribing on ITO glass substrates ($6\times 6\,{{{{\rm{cm}}}}}^{2}$ or $30\times 40\,{{{{\rm{cm}}}}}^{2}$) to define the P1 region (average laser power, $5\,{{{\rm{W}}}}$; pulse frequency, $700\,{{\mathrm{kHz}}}$; spot diameter, $10\,{{{\rm{\mu }}}}{{{\rm{m}}}}$; scribing line width, $20\,{{{\rm{\mu }}}}{{{\rm{m}}}}$; scribing speed, $1000\,{{\mathrm{mm}}}\,{{{{\rm{s}}}}}^{-1}$). The scribed substrates were then sent to an in-line cleaning machine and ultrasonically cleaned with diluted detergent and deionized water. After cleaning, the substrates were blown dry with compressed dry air and treated with UV-ozone for 15 min.

Subsequently, the NiO_x_ and 3PATAT-C3 layers were deposited sequentially by slot-die coating. For the NiO_x_ film, slot-die coating was performed on a heated stage at 70 °C, with a solution flow rate of $0.6{{{\rm{mL}}}}{\min }^{-1}$, a coating speed of $13\,{{\mathrm{mm}}}\,{{{{\rm{s}}}}}^{-1}$ and a head gap of $80\,{{{\rm{\mu }}}}{{{\rm{m}}}}$. After coating, the substrate was transferred to a hot plate and annealed at 350 °C for $10\,\min$, and then allowed to cool completely to room temperature. The 3PATAT-C3 layer was subsequently deposited by slot-die coating under the following conditions: solution flow rate, $0.5\,{{\mathrm{mL}}}\,{\min }^{-1}$; coating speed, $13\,{{\mathrm{mm}}}\,{{{{\rm{s}}}}}^{-1}$; and head gap, $80\,{{{\rm{\mu }}}}{{{\rm{m}}}}$. After coating, the substrate was transferred to a hot plate and heated at 100 °C for $10\,\min$, followed by cooling to room temperature.

Next, the perovskite film was deposited on the ITO/NiO_x_/3PATAT-C3 substrate via slot-die coating under the following conditions: solution flow rate, $0.4\,{{\mathrm{mL}}}\,{\min }^{-1}$; coating speed, $10\,{{{\rm{mm}}}}{{{{\rm{s}}}}}^{-1}$; and coating head gap, $60\,{{{\rm{\mu }}}}{{{\rm{m}}}}$. After coating, the wet perovskite films were cooled for 15 s, reaching an experimentally measured film temperature of 0−10 °C immediately before vacuum treatment (Supplementary Fig. 17). The films were then transferred to the vacuum-flash chamber within approximately 5 s. Vacuum flashing was performed in a chamber with an effective internal volume of approximately $69.6\,{{{{\rm{cm}}}}}^{3}$ for a total treatment time of 300 s. The chamber pressure was initially approximately $1.0\times {10}^{5}\,{{{\rm{Pa}}}}$ and decreased to below 10 Pa within approximately 8 s. During the remaining approximately 292 s, the pressure gradually decreased from approximately 10 Pa to approximately 5 Pa, reaching a final pressure of approximately 5 Pa (Supplementary Fig. 18). The films were then annealed at 80 °C for 2 min and then at 150 °C for 10 min. Thereafter, a passivation layer of EDAI_2_ ($0.1\,{{\mathrm{mg}}}\,{{{\mathrm{mL}}}}^{-1}$) was coated onto the perovskite film by slot-die coating under the following conditions: solution flow rate, $2.5\,{{\mathrm{mL}}}\,{\min }^{-1}$; coating speed, $30{{{\rm{mm}}}}{{{{\rm{s}}}}}^{-1}$; head gap, 50 μm; followed by annealing at 85 °C for 2 min. Finally, 20 nm of C_60_ and 5 nm of BCP were deposited on the perovskite film by thermal evaporation. After BCP deposition, a second laser-scribing process using a 532-nm laser scriber (average laser power, 0.3 W; pulse frequency, 500 kHz; spot diameter, $30\,{{{\rm{\mu }}}}{{{\rm{m}}}}$; scribing line width, 50 μm; scribing speed, $1000\,{{\mathrm{mm}}}\,{{{{\rm{s}}}}}^{-1}$) was applied to define the P2 region. The module stack was then completed by thermal evaporation of a 120-nm silver electrode. Finally, monolithic interconnection of the sub-cells was achieved by laser scribing using a 532-nm laser system with optimized parameters (average laser power, 0.5 W; pulse frequency, 700 kHz; spot diameter, $30\,{{{\rm{\mu }}}}{{{\rm{m}}}}$; scribing line width, 70 μm; scribing speed: $1000\,{{\mathrm{mm}}}\,{{{{\rm{s}}}}}^{-1}$) to form the P3 region. This process enables the fabrication of either a $19.48\,{{{{\rm{cm}}}}}^{2}$ aperture-area minimodule (eight cells in series) or a larger module with an aperture area of $829.92\,{{{{\rm{cm}}}}}^{2}$ (fifty-six cells in series), both with a geometric fill factor of 97%. For the champion module, a 150-nm MgF_2_ anti-reflection layer was deposited on the glass side by thermal evaporation.

### Characterizations

#### Film and device characterization

Field-emission scanning electron microscopy (FESEM) was performed using a Hitachi S-4800 microscope. X-ray diffraction (XRD) patterns were recorded from ITO/HTL/perovskite films at a scan rate of $5^\circ \,{\min }^{-1}$ using a Rigaku Ultima IV X-ray diffractometer with Cu Kα Radiation ($0.15406\,{{\mathrm{nm}}}$). Photoluminescence (PL) spectra were recorded using a PTI QM-40 spectrofluorometer with an excitation wavelength of 500 nm. For PL measurements, the perovskite films were directly deposited on quartz substrates. Energy-dispersive X-ray spectroscopy (EDS) measurements were performed using a scanning electron microscope (Gemini 360, ZEISS) equipped with an EDS detector (ULTIM MAX 100, OXFORD). The measurements were conducted at an accelerating voltage of 15 kV and a working distance of 8.5 mm. Electroluminescence (EL) images of the mini-modules were acquired in a dark environment after P3 laser scribing. A digital camera (Sony A7R II equipped with a Sony FE 50 mm lens) was mounted above the mini-module, and the EL images were recorded under a fixed injection current of 20 mA supplied by a Wanptek power source (15 V). Ultraviolet-visible (UV-vis) absorption spectra were recorded using a Shimadzu UV-2450 spectrophotometer. Atomic force microscopy (AFM) and Kelvin probe force microscopy (KPFM) were performed using a Bruker MultiMode 8 system. Space-charge-limited current (SCLC) measurements were conducted using electron-only devices. The thickness and relative dielectric constant used in SCLC analysis were $500\,{{\mathrm{nm}}}$ and 20 respectively. The I−V curves of SCLC devices were measured with a Keithley 2400 source meter in the dark and recorded from 0 to 2 V at a voltage step of 0.01 V with a delay time of 0.2 s. The electron mobility (μ) was derived by fitting the J−V curves using the Mott–Gurney law. External quantum efficiency (EQE) spectra were acquired using an integrated system (QER3018, EnliTech).

#### In situ photoluminescence

In situ photoluminescence spectra were acquired using a home-built system comprising an excitation source, optical fibres and a detector. The samples were placed in a vacuum-flash chamber equipped with optical fibres for excitation and signal collection. A 425-nm laser was used as the excitation source, and the emitted light was collected through optical fibres and recorded using a spectrophotometer. A 450-nm long-pass filter was placed in the optical path before detection.

#### Electrochemical impedance spectroscopy

Electrical impedance spectroscopy (EIS) of the modules was measured over the frequency range from 1 Hz to 1 MHz with a 7 V external bias under dark conditions using a PARSTAT 3000A-DX system, and the results were fitted by ZView2.

#### Photovoltaic measurements

Current–voltage *(J–V*) characteristics were measured under AM 1.5 G illumination (100 mW cm^−2^) using a Class A solar simulator equipped with a xenon lamp. The light intensity was calibrated using a Newport-calibrated monocrystalline silicon reference cell.

#### Suns-${{{{\boldsymbol{V}}}}}_{{{{\boldsymbol{oc}}}}}$ measurements

To study carrier recombination before and after treatment, we used the light-intensity-dependent ${V}_{{oc}}$ method (Suns-${V}_{{oc}}$) by measuring the ${V}_{{oc}}$ values of the devices under different light intensities. Different light intensities were obtained by adjusting the grating of the solar simulator and calibrated using a Newport-calibrated monocrystalline Si diode.

### Stability measurements

#### Illumination-stability measurements

For illumination-stability measurements, encapsulated modules were tested under continuous open-circuit one-sun illumination (100 mW cm^−2^, xenon lamp) under ambient conditions.

#### UV-ageing tests

UV-ageing tests were conducted in a standard UV-ageing chamber (GRO-SUV3226T, Shanghai Reichy). The chamber temperature was maintained at 60 ± 5 °C. The combined UVA and UVB irradiance was $200\,\pm \,30\, {{{\rm{W}}}}{{{{\rm{m}}}}}^{-2}$, with a UVB/UVA ratio of 5−9%, in accordance with IEC 61215.

## Supplementary information

Supplementary Information

Transparent Peer Review file

## Source data

Source data

## Data Availability

All data supporting the findings of this study are available within this article, its Supplementary Information and the accompanying Source Data file. Source data are provided with this paper.
