# Supplementary material for: Retarding efficiency loss upon scaling in perovskite photovoltaics as fill factor becomes the bottleneck
Source: Nat Commun. 2026 Oct 29;17:11641. doi: 10.1038/s41467-026-78867-1 (PMC13760041; doi:10.1038/s41467-026-78867-1)
Supplement: Supplementary file 1 [file 41467_2026_78867_MOESM1_ESM.pdf]

Supplementary Information for:

Retarding efficiency loss upon scaling in perovskite photovoltaics as fill factor becomes the bottleneck

Buyi Yan[1] *#, Ruihao Gong[2] #, Yanchun Huang[1], Nannan Gu[1], Runfeng Li[2], Dongxue Liu[3], Kangrong Yan[1], Xuehui Xu[4], Yang (Michael) Yang[4], Jizhong Yao[1] *, Dongchen Lan[2,5,6] *#

[1]Microquanta Semiconductor Co., Ltd., Hangzhou, 311121, China

[2]College of Electrical Engineering, Zhejiang University, Hangzhou, 310027, China

[3]China Three Gorges Renewable (Group) Co., Ltd., Beijing, 101199, China

[4]State Key Laboratory of Modern Optical Instrumentation, Institute of Advanced Photonics, College of Optical Science and Engineering, Zhejiang University, Hangzhou, Zhejiang 310027, China

[5]Australian Centre for Advanced Photovoltaics, Sydney, 2052, Australia

[6]Zhejiang Provincial Key Laboratory of Optoelectronic Functional Materials and Devices, Hangzhou 311200, China

#These authors contributed equally: Buyi Yan, Ruihao Gong, Dongchen Lan.

*Correspondence: buyi.yan@microquanta.com (B. Yan), jizhong.yao@microquanta.com (J. Yao), d.lan@zju.edu.cn (D. Lan)

Lead contact: Dongchen Lan

Table of Contents

Supplementary Notes 1-4

Supplementary Figures 1-18

Supplementary Tables 1-5

Supplementary References

Supplementary Note 1 | Quantifying the origins of cell-to-module efficiency loss

To quantify the contributions of different photovoltaic parameters to cell-to-module efficiency loss, a multiplicative attribution framework was employed. The power conversion efficiency (PCE) of a solar cell or module can be expressed as:

$$\eta = V_{OC} J_{SC} FF \tag{S1}$$

where V_{oc} is open-circuit voltage, J_{sc} is short-circuit current density and FF is fill factor.

The reference cell parameters were denoted as V_{OC0}, J_{SC0} and FF_0, and the corresponding module parameters were denoted as V_{OCm}, J_{SCm} and FF_m. The total cell-to-module PCE loss was calculated as:

$$\Delta\eta = \eta_0 - \eta_m = V_{OC0}J_{SC0}FF_0 - V_{OCm}J_{SCm}FF_m \tag{S2}$$

where η_0 and η_m are the PCEs of the reference cell and module, respectively.

The losses in the photovoltaic parameters were defined as:

$$\Delta V_{OC} = V_{OC0} - V_{OCm} \tag{S3}$$

$$\Delta J_{SC} = J_{SC0} - J_{SCm} \tag{S4}$$

$$\Delta FF = FF_0 - FF_m \tag{S5}$$

A positive value therefore represents a loss during the cell-to-module transition.

Because PCE is the product of V_{oc}, J_{sc} and FF, the total cell-to-module efficiency loss cannot be uniquely assigned to individual photovoltaic parameters without accounting for interaction terms among them. To avoid this ambiguity, a Shapley-value-based multiplicative decomposition was employed to allocate the interaction effects in an order-independent manner[1,2]. The contributions from V_{oc}, J_{sc} and FF were calculated as:

$$C_{Voc} = \Delta V_{OC}J_{SC0}FF_0 - \frac{1}{2}\Delta V_{OC}\Delta J_{SC}FF_0 - \frac{1}{2}\Delta V_{OC}J_{SC0}\Delta FF + \frac{1}{3}\Delta V_{OC}\Delta J_{SC}\Delta FF \tag{S6}$$

$$C_{Jsc} = V_{OC0}\Delta J_{SC}FF_0 - \frac{1}{2}\Delta V_{OC}\Delta J_{SC}FF_0 - \frac{1}{2}V_{OC0}\Delta J_{SC}\Delta FF + \frac{1}{3}\Delta V_{OC}\Delta J_{SC}\Delta FF \tag{S7}$$

$$C_{FF} = V_{OC0}J_{SC0}\Delta FF - \frac{1}{2}\Delta V_{OC}J_{SC0}\Delta FF - \frac{1}{2}V_{OC0}\Delta J_{SC}\Delta FF + \frac{1}{3}\Delta V_{OC}\Delta J_{SC}\Delta FF \tag{S8}$$

where C_{Voc}, C_{Jsc} and C_{FF} represent the PCE losses attributed to V_{oc}, J_{sc} and FF, respectively. This decomposition satisfies $C_{Voc} + C_{Jsc} + C_{FF} = \Delta\eta$ and therefore conserves the total PCE loss.

To further identify the origin of current-density loss, the total J_{sc}-related contribution, C_{Jsc}, was partitioned into laser-scribing-related and residual components. Assuming that the short-circuit current density scales linearly with the photoactive area, the current density expected after accounting only for the geometrical fill factor (GFF) is:

$$J_{SC,GFF} = J_{SC0} \times GFF \tag{S9}$$

The corresponding current-density losses were calculated as:

$$\Delta J_{SC,GFF} = J_{SC0} - J_{SC,GFF} \tag{S10}$$

$$\Delta J_{SC,other} = J_{SC,GFF} - J_{SCm} \tag{S11}$$

The total J_{sc}-related PCE loss was then apportioned according to the relative contributions of the two current-density losses:

$$C_{Jsc,GFF} = C_{Jsc} \frac{\Delta J_{SC,GFF}}{\Delta J_{SC}} \tag{S12}$$

$$C_{Jsc,other} = C_{Jsc} \frac{\Delta J_{SC,other}}{\Delta J_{SC}} \tag{S13}$$

where $C_{Jsc,GFF}$ and $C_{Jsc,other}$ represent the PCE losses attributed to GFF-related and residual current-loss mechanisms, respectively.

The reported V_{oc}, J_{sc} and FF values for each paired cell–module dataset were analysed using the framework above, yielding the corresponding contributions of V_{oc}, J_{sc} and FF to the total cell-to-module efficiency loss. By construction, the resulting attribution exactly conserves the measured cell-to-module loss.

Supplementary Note 2 | Stepwise analysis of fill-factor deficits

The fill factor (FF) of a practical photovoltaic device generally falls below its theoretical limit as its operating parameters deviate from their ideal values. To quantify the contributions associated with these deviations, we progressively introduce device-specific parameters into a common analytical framework. We employed the single-diode model, in which the device current density–voltage characteristic is expressed as[3]:

$$J = J_{ph} - J_0 \left[\exp\left(\frac{q(V + JR_s)}{n_{id} kT} \right) - 1 \right] - \frac{V + JR_s}{R_{sh}} \tag{S14}$$

where J_{ph} is the photogenerated current density, J_0 is dark saturation current density, R_s is series resistance, R_{sh} is shunt resistance, q is elementary charge, n_{id} is ideality factor, k is Boltzmann constant, and T is the absolute temperature.

The fill factor can be expressed as the product of a resistance-free fill factor (FF_0) and a dimensionless resistive correction factor, $f_{tr}(R_s, R_{sh})$, accounting for parasitic series- and shunt-resistance effects[4]:

$$FF = FF_0(V_{oc}, n_{id})\, f_{tr}(R_s, R_{sh}) \tag{S15}$$

In the absence of parasitic resistance effects ($R_s = 0$ and $R_{sh} \rightarrow \infty$), FF_0 can be approximated for sufficiently large normalized open-circuit voltage (v_{oc}) as[3]:

$$FF_0(V_{oc}, n_{id}) = \frac{v_{oc} - \ln(v_{oc} + 0.72)}{v_{oc} + 1}, \quad v_{oc} = \frac{qV_{oc}}{n_{id} kT}. \tag{S16}$$

At the Shockley-Queisser (SQ) limit[5], $V_{oc} = V_{oc}^{SQ}$, $n_{id} = 1$, and parasitic-resistance losses are absent ($f_{tr} = 1$). Accordingly, the SQ-limit fill factor (FF_{SQ}) is evaluated using the same resistance-free FF expression as

$$FF_{SQ} = FF_0\left(V_{oc}^{SQ}, 1\right) \tag{S17}$$

where V_{oc}^{SQ} is the open-circuit voltage at the SQ limit.

Starting from this SQ-limit reference, the FF deficit of a practical device is analysed stepwise. The first two steps remain resistance-free while progressively introducing the measured V_{oc} and the diode ideality factor n_{id}; parasitic-resistance effects are introduced only in the final step:

$$FF_{SQ} \xrightarrow{V_{oc}} FF_0(V_{oc}, 1) \xrightarrow{n_{id}} FF_0(V_{oc}, n_{id}) \xrightarrow{R_s, R_{sh}} FF$$

The FF reduction at each step shown above defines the V_{oc}-related, n_{id}-associated and transport-associated contributions, respectively.

First, V_{oc}^{SQ} is replaced by the measured V_{oc}, while n_{id} is retained at unity and the resistance-free condition is maintained. The resulting V_{oc}-related FF deficit is

$$\Delta FF_{V_{oc}} = FF_{SQ} - FF_0(V_{oc}, 1). \tag{S18}$$

Next, the diode ideality factor n_{id} is introduced while maintaining the measured V_{oc} and the resistance-free condition. The resulting n_{id}-associated FF deficit is

$$\Delta FF_{n_{id}} = FF_0(V_{oc}, 1) - FF_0(V_{oc}, n_{id}). \tag{S19}$$

This stepwise analysis separately quantifies the FF contributions associated with V_{oc} and n_{id}. Physically, the V_{oc}-related FF deficit can be regarded as recombination-related, because V_{oc} is governed by the balance between photogeneration and recombination[6]. By contrast, changes in n_{id} in perovskite devices may arise from multiple coupled factors, including shallow defects, interfacial effects, transport limitations and other factors[7-9].

Finally, while maintaining the measured V_{oc} and n_{id}, parasitic-resistance effects are introduced through $f_{tr}(R_s, R_{sh})$, as in Eq. S15. The resulting reduction from the resistance-free $FF_0(V_{oc}, n_{id})$ to the measured FF is regarded as the transport-associated FF deficit (ΔFF_{tr}). Accordingly, ΔFF_{tr} is given by

$$\Delta FF_{tr} = FF_0(V_{oc}, n_{id}) - FF = FF_0(V_{oc}, n_{id})\,[1 - f_{tr}(R_s, R_{sh})]. \tag{S20}$$

The total FF deficit is therefore expressed as

$$\Delta FF = FF_{SQ} - FF = \Delta FF_{V_{oc}} + \Delta FF_{n_{id}} + \Delta FF_{tr}. \tag{S21}$$

Within the single-diode model, the transport-associated contribution is represented by parasitic series- and shunt-resistance effects. The resistive correction factor $f_{tr}(R_s, R_{sh})$ is evaluated using the explicit analytical expression derived by Green[4]. This expression was shown to be accurate for $v_{oc} > 10$ when the total parasitic-resistance-induced FF loss is below ~ 5%, conditions generally satisfied by well-performing photovoltaic devices.

The transport-associated FF deficit can be approximated as[4]:

$$\Delta FF_{tr} \approx FF_0(V_{oc}, n_{id}) \times \left(\frac{IF_0}{VF_0}\, r_s + \frac{VF_0}{IF_0}\, \frac{1}{r_{sh}} \right) \tag{S22}$$

where

$$r_s = \frac{R_s\, J_{sc}}{V_{oc}}, \qquad r_{sh} = \frac{R_{sh}\, J_{sc}}{V_{oc}},$$

and

$$IF_0 = \frac{J_{mp,0}}{J_{sc}}, \qquad VF_0 = \frac{V_{mp,0}}{V_{oc}}.$$

$J_{mp,0}$ and $V_{mp,0}$ denote the current density and voltage at the maximum power point of the resistance-free reference characteristic, respectively, and are calculated analytically following Green's formulation[4].

This allows further decomposition into series- and shunt- resistance contributions:

$$\Delta FF_{R_s} \approx FF_0(V_{oc}, n_{id}) \frac{IF_0}{VF_0} r_s \tag{S23}$$

$$\Delta FF_{R_{sh}} \approx FF_0(V_{oc}, n_{id}) \frac{VF_0}{IF_0} \frac{1}{r_{sh}}. \tag{S24}$$

In practice, V_{oc} and FF were obtained from the measured current–voltage characteristics, while n_{id}, R_s and R_{sh} were extracted by fitting the corresponding curves using the single-diode model. The fitted n_{id} and measured V_{oc} were used to calculate $FF_0(V_{oc}, n_{id})$. This framework underpins the FF-deficit analyses shown in Fig. 1d, Fig. 4c, e and Fig. 5c, f.

Supplementary Note 3 | Influence of open-circuit voltage and ideality factor on fill factor

As described in Supplementary Note 2, the measured fill factor (FF) can be expressed as the product of the resistance-free fill factor FF_0 and the resistive correction factor $f_{tr}(R_s, R_{sh})$, that is, $FF = FF_0(V_{oc}, n_{id})\, f_{tr}(R_s, R_{sh})$. We therefore compared the area-dependent evolution of FF, $FF_0(V_{oc}, n_{id})$ and $f_{tr}(R_s, R_{sh})$ for the control and target devices to first assess the relative importance of the resistance-free and parasitic-resistance-related contributions to the observed FF variation.

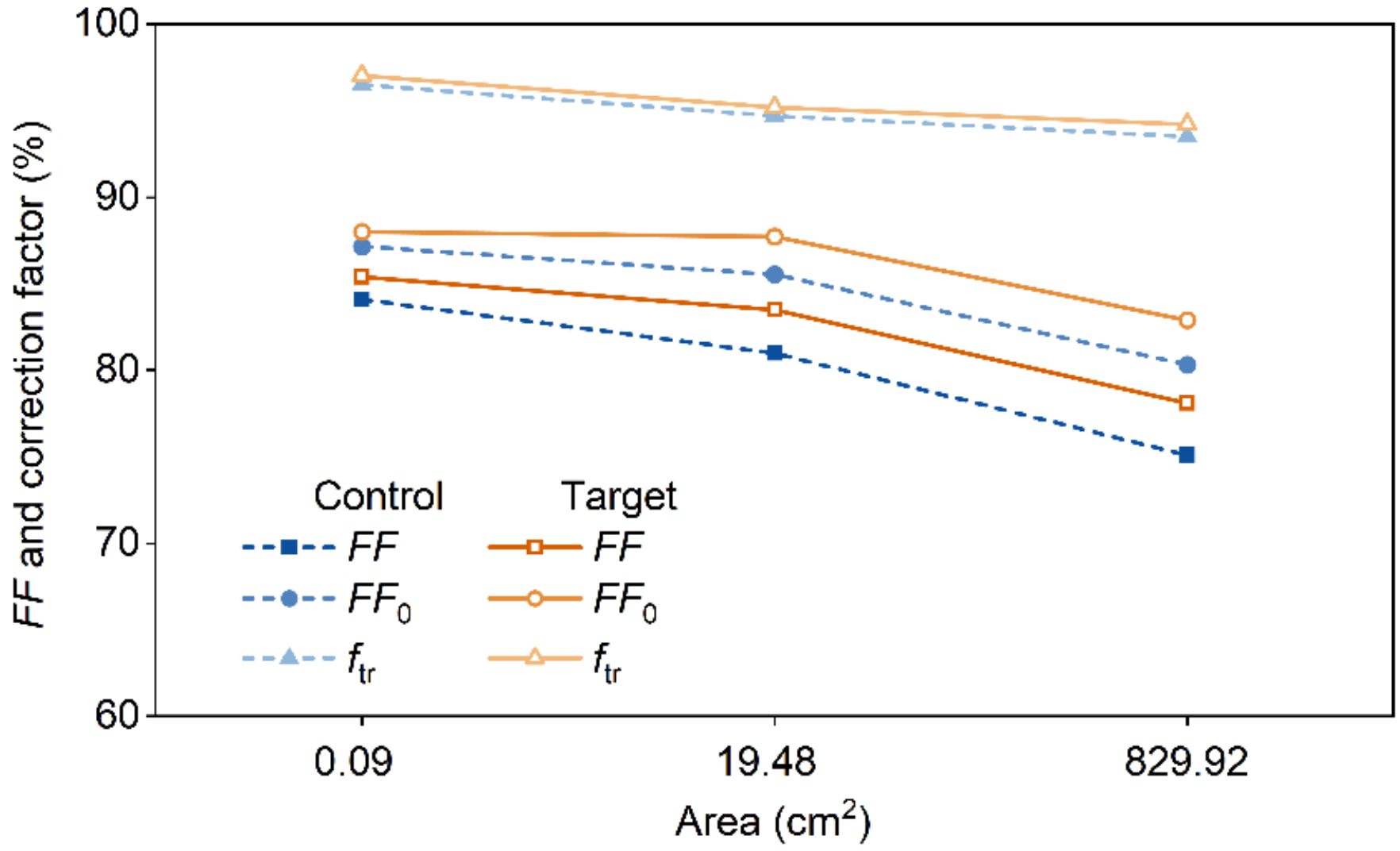

Supplementary Note Figure 1 | Evolution of FF, FF_0 and f_{tr} with device area.

As shown in Supplementary Note Fig. 1, $f_{tr}(R_s, R_{sh})$ varies only modestly with device area and between the control and target devices. By contrast, $FF_0(V_{oc}, n_{id})$ shows substantially larger variations with device area and between the control and target devices, with a trend closely resembling that of the measured FF. This comparison indicates that the observed FF variation is primarily associated with changes in the resistance-free FF_0.

Accordingly, the respective influences of n_{id} and V_{oc} on the observed FF variation can be assessed primarily through their effects on FF_0. We therefore calculated FF_0 as a function of n_{id} at several fixed V_{oc} values. In the figure below, each experimental symbol represents FF_0 calculated from the corresponding measured V_{oc} and n_{id}.

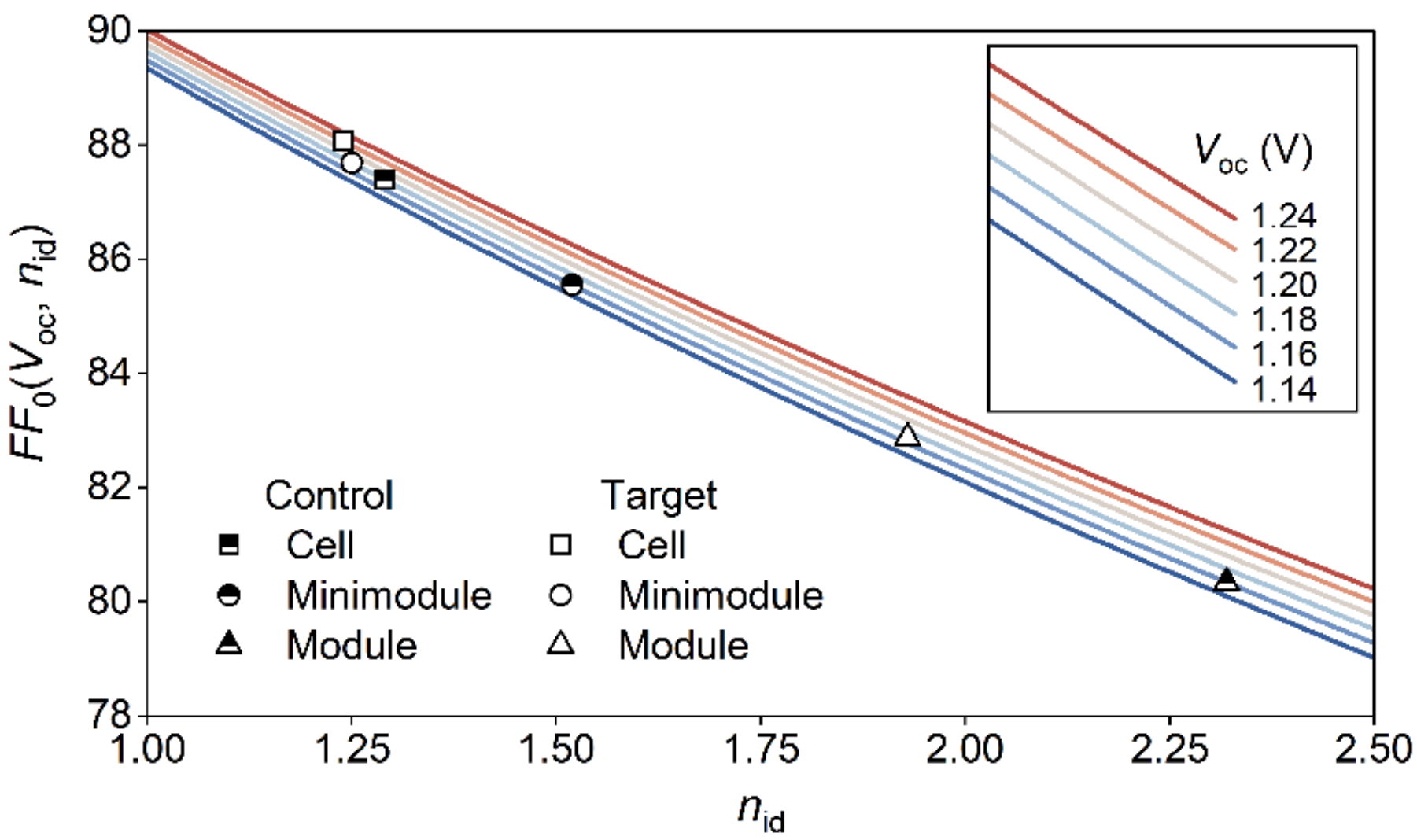

Supplementary Note Figure 2 | Dependence of the resistance-free fill factor on n_{id} at fixed V_{oc} values.

As shown in Supplementary Note Fig. 2, $FF_0(V_{oc}, n_{id})$ decreases monotonically with increasing n_{id} at each fixed V_{oc}, whereas increasing V_{oc} shifts the calculated curves towards higher FF_0. The experimental data further allow the relative influences of V_{oc} and n_{id} to be compared across device scales.

Relative to the corresponding control devices, the target cells, mini-modules and modules show reductions in n_{id} of 0.05, 0.27 and 0.39, respectively. The corresponding increases in $FF_0(V_{oc}, n_{id})$ are 0.68, 2.14 and 2.54 percentage points. By contrast, the target–control V_{oc} gain decreases from 0.04 V for the cells to 0.02 V for the mini-modules and 0.01 V for the modules. Thus, during area scaling, the V_{oc} advantage progressively diminishes, whereas the reduction in n_{id} and the gain in $FF_0(V_{oc}, n_{id})$ become increasingly pronounced. These opposing scale-dependent trends demonstrate that the increasing FF_0 advantage with scaling arises predominantly from the reduction in n_{id} rather than from the diminishing V_{oc} difference. Consistently, the stepwise analysis in Fig. 5c shows that the n_{id}-associated contribution to the FF gain becomes increasingly prominent with scaling, while the V_{oc}-related and transport-associated contributions remain small.

Supplementary Note 4 | Impact of series resistance on fill-factor loss during area scaling

During the scale-up of thin-film perovskite photovoltaics from small-area cells to monolithically interconnected modules, charge collection involves an additional lateral transport component within each sub-cell. This lateral current transport is commonly considered a potential source of fill-factor (FF) loss because it can increase the effective series resistance associated with charge extraction.

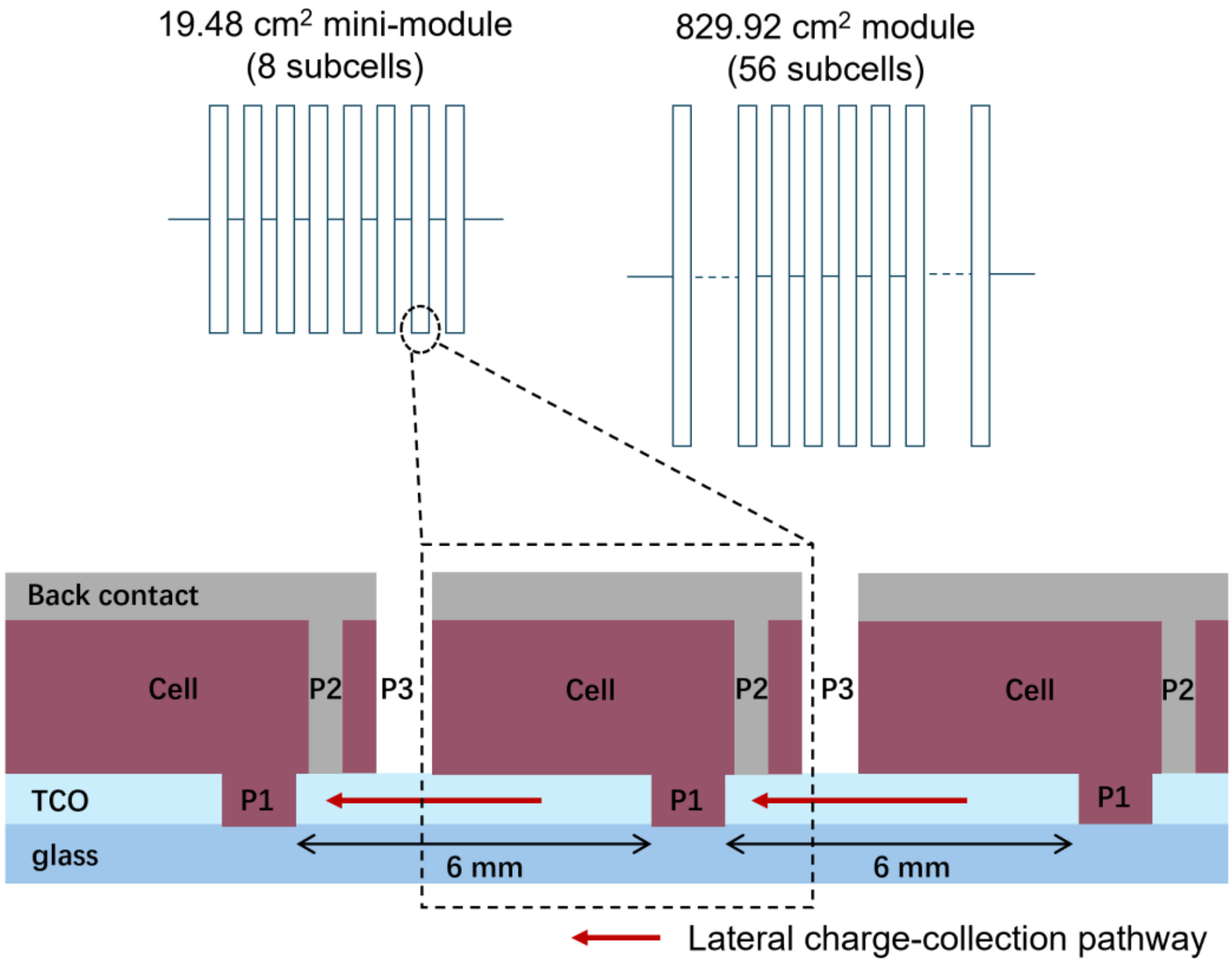

Supplementary Note Figure 3 | Schematic illustration of lateral current transport in perovskite cells and modules.

As shown in Supplementary Note Fig. 3, monolithically interconnected mini-modules introduce lateral current transport within each sub-cell as part of charge collection and interconnection. Further scaling from mini-modules to practical large-area modules does not necessarily introduce additional lateral transport pathways when the sub-cell geometry remains unchanged.

To assess the significance of this FF-loss mechanism during scale-up, the measured FF deficit was separated into V_{oc}-related, n_{id}-associated, series-resistance-related (R_s) and shunt-resistance-related (R_{sh}) components using the framework described in Supplementary Note 2.

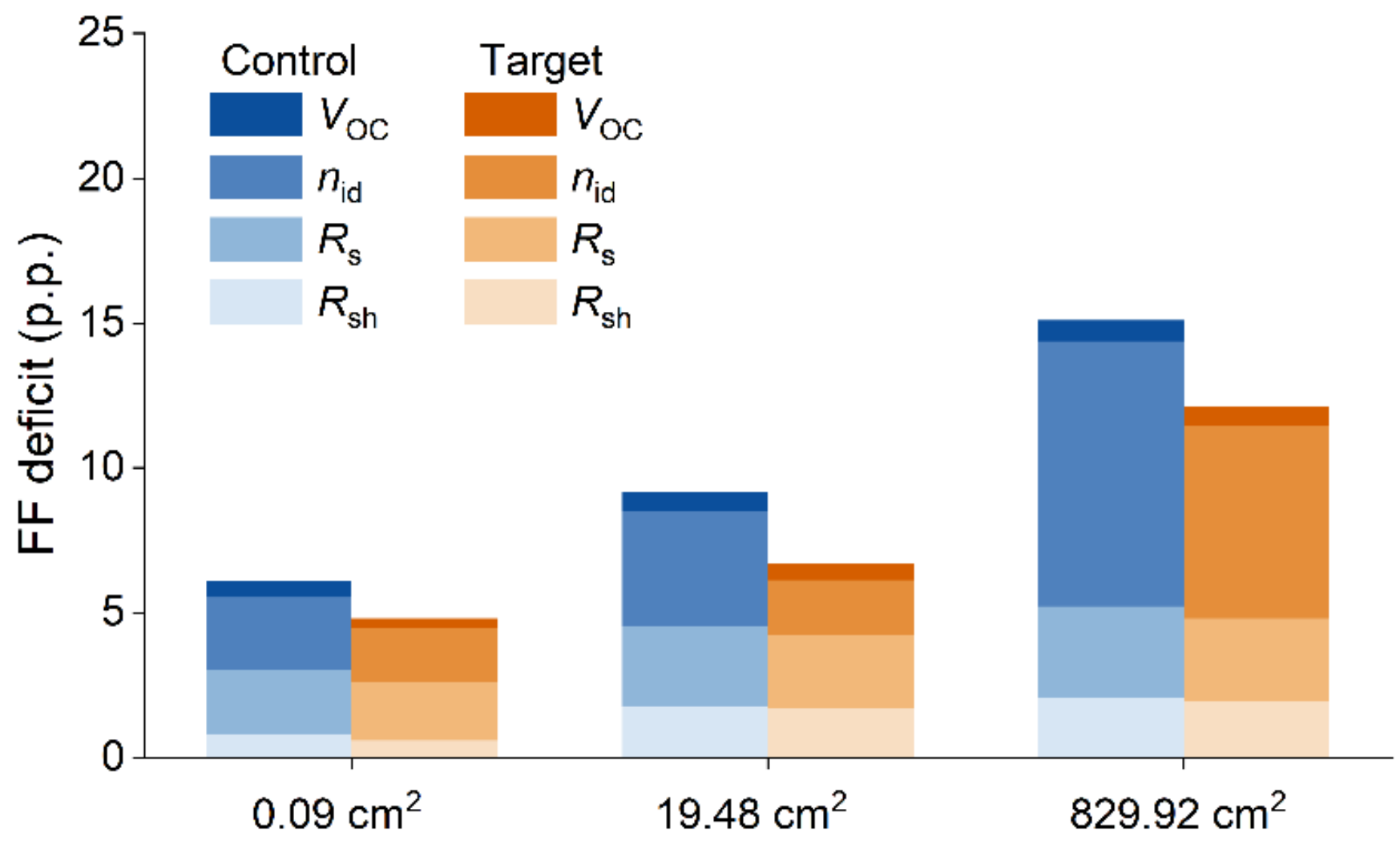

Supplementary Note Figure 4 | *FF*-deficit components for control and target devices across different scales.

As shown in Supplementary Note Fig. 4, the overall FF deficit increases progressively during area scaling for both the control and target devices. Although the series-resistance-related component also increases during scaling, its increase remains modest compared with the corresponding increase in the n_{id}-associated component.

For example, for the control devices, the n_{id}-associated FF deficit increases from $2.48\,\text{p.p.}$ for $0.09\,\text{cm}^2$ cells to $3.95\,\text{p.p.}$ for $19.48\,\text{cm}^2$ mini-modules and further to $9.16\,\text{p.p.}$ for $829.92\,\text{cm}^2$ modules. By comparison, the corresponding series-resistance-related FF deficit increases only from $2.24\,\text{p.p.}$ to $2.76\,\text{p.p.}$ and $3.14\,\text{p.p.}$, respectively. A similar trend is observed for the target devices.

Therefore, the progressive increase in FF deficit during scaling is dominated by n_{id}-associated losses rather than series-resistance-related losses. Although lateral current transport contributes to FF degradation through increased series resistance, it accounts for only a small fraction of the scaling-induced FF loss. This finding supports the conclusion in the main text that elongated lateral charge-collection pathways are not the primary origin of the observed FF reduction.

To further identify the origin of the improved FF preservation achieved by cryo-controlled processing, the contributions of different loss-reduction mechanisms to the FF improvement were quantified relative to the corresponding control devices.

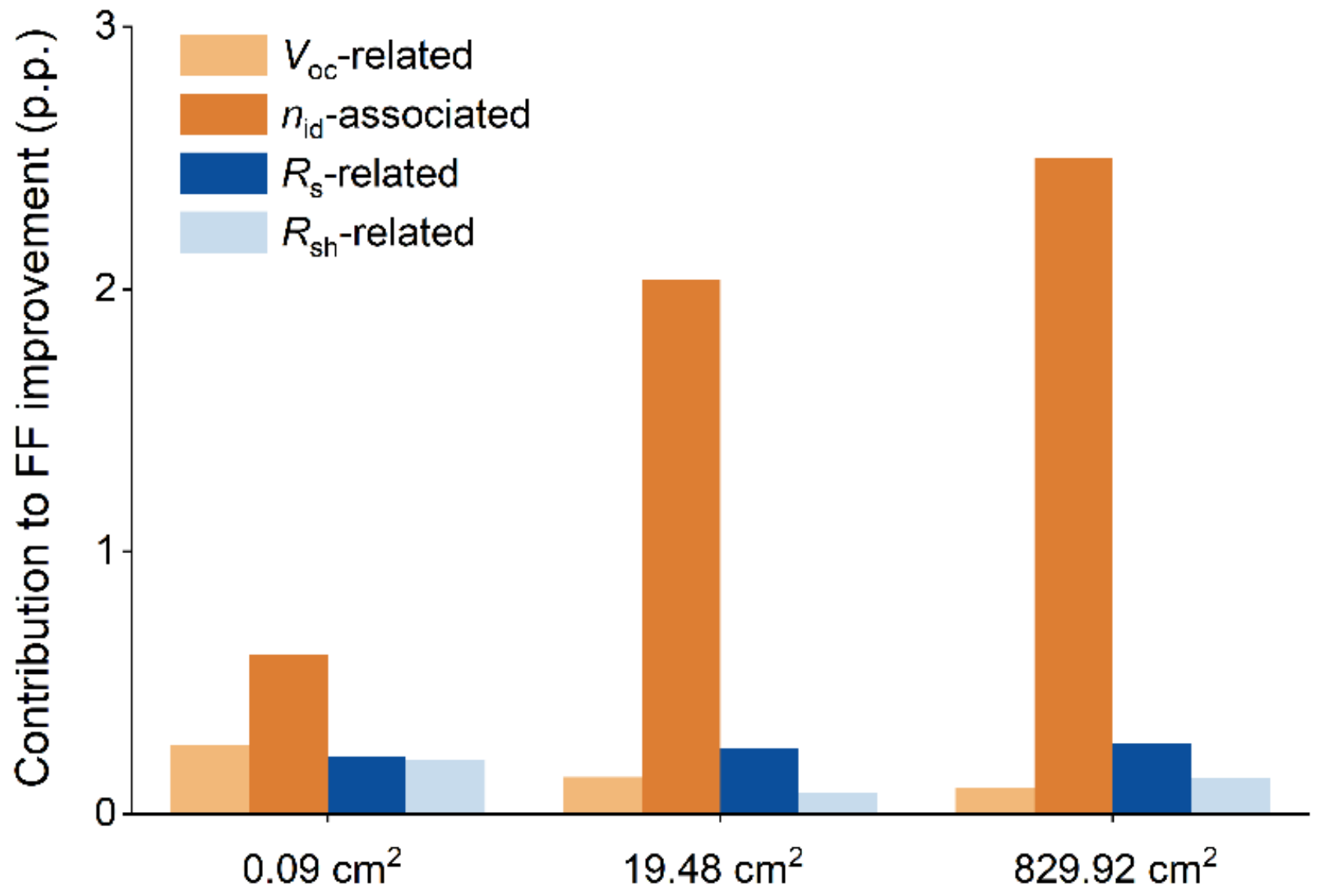

Supplementary Note Figure 5 | Contributions of different loss reductions to fill-factor improvement.

As shown in Supplementary Note Fig. 5, the FF improvement achieved by cryo-controlled processing increases with device size, consistent with the progressively larger FF deficit observed during scaling.

For the $19.48\ \mathrm{cm}^2$ mini-module, cryo-controlled processing improves FF by $2.51\ \mathrm{p.p.}$, of which $0.14\ \mathrm{p.p.}$ originates from reduced V_{oc}-related loss, $2.04\ \mathrm{p.p.}$ originates from reduced n_{id}-associated loss, $0.25\ \mathrm{p.p.}$ originates from reduced series-resistance-related loss and $0.08\ \mathrm{p.p.}$ originates from reduced shunt-resistance-related loss. Similar trends are observed for the $0.09\ \mathrm{cm}^2$ cells and the $829.92\ \mathrm{cm}^2$ modules. Additionally, the FF advantage of the target relative to the control increases with device size, suggesting enhanced effectiveness of cryo-controlled processing at larger device scales.

These results demonstrate that the superior FF of the target devices manifests predominantly through the reduced n_{id}-associated contribution, whereas reductions in series-resistance-related losses provide a secondary contribution.

Collectively, the above analysis shows that series-resistance-related loss associated with lateral current transport is not the primary origin of the FF reduction observed during scale-up. Instead, the increasing FF deficit from cells to modules manifests predominantly through the n_{id}-associated diode-curvature contribution. Furthermore, the superior FF preservation achieved by cryo-controlled processing is dominated by the reduced n_{id}-associated contribution, while the series-resistance-related contribution remains secondary.

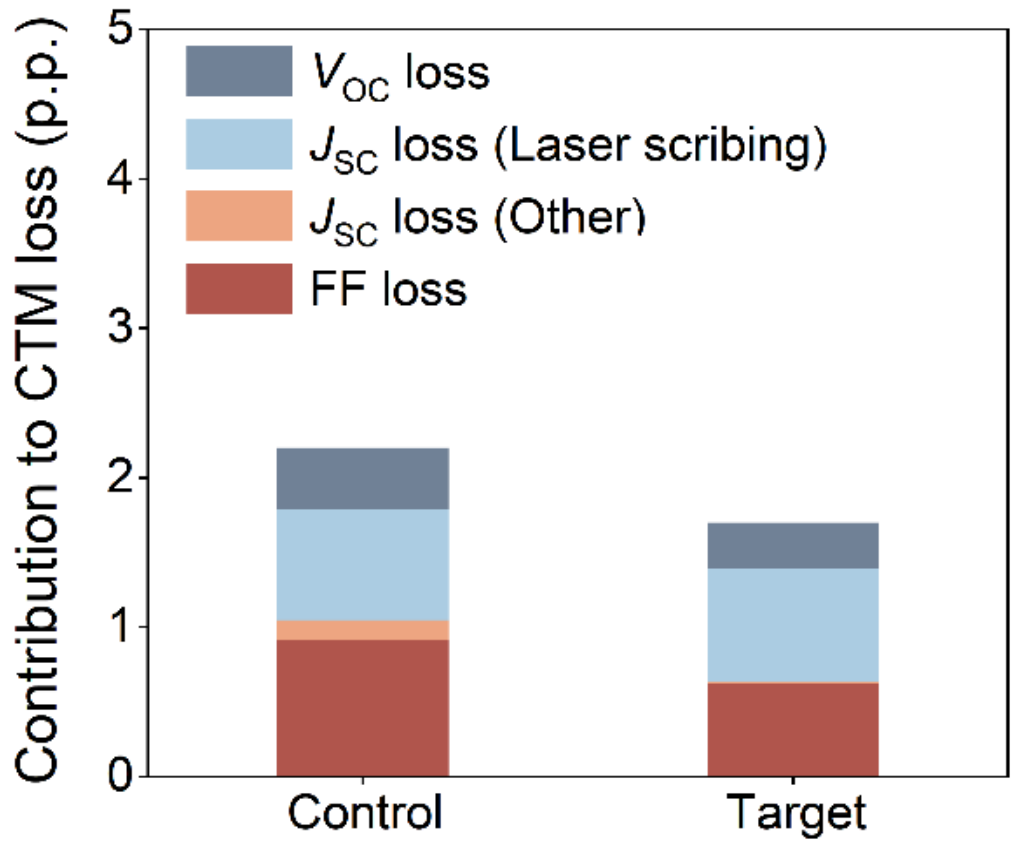

Supplementary Figure 1 | Decomposition of the cell-to-module loss. Decomposition of the absolute cell-to-module efficiency loss into contributions from V_{oc}, J_{sc} and FF for control and target perovskite devices.

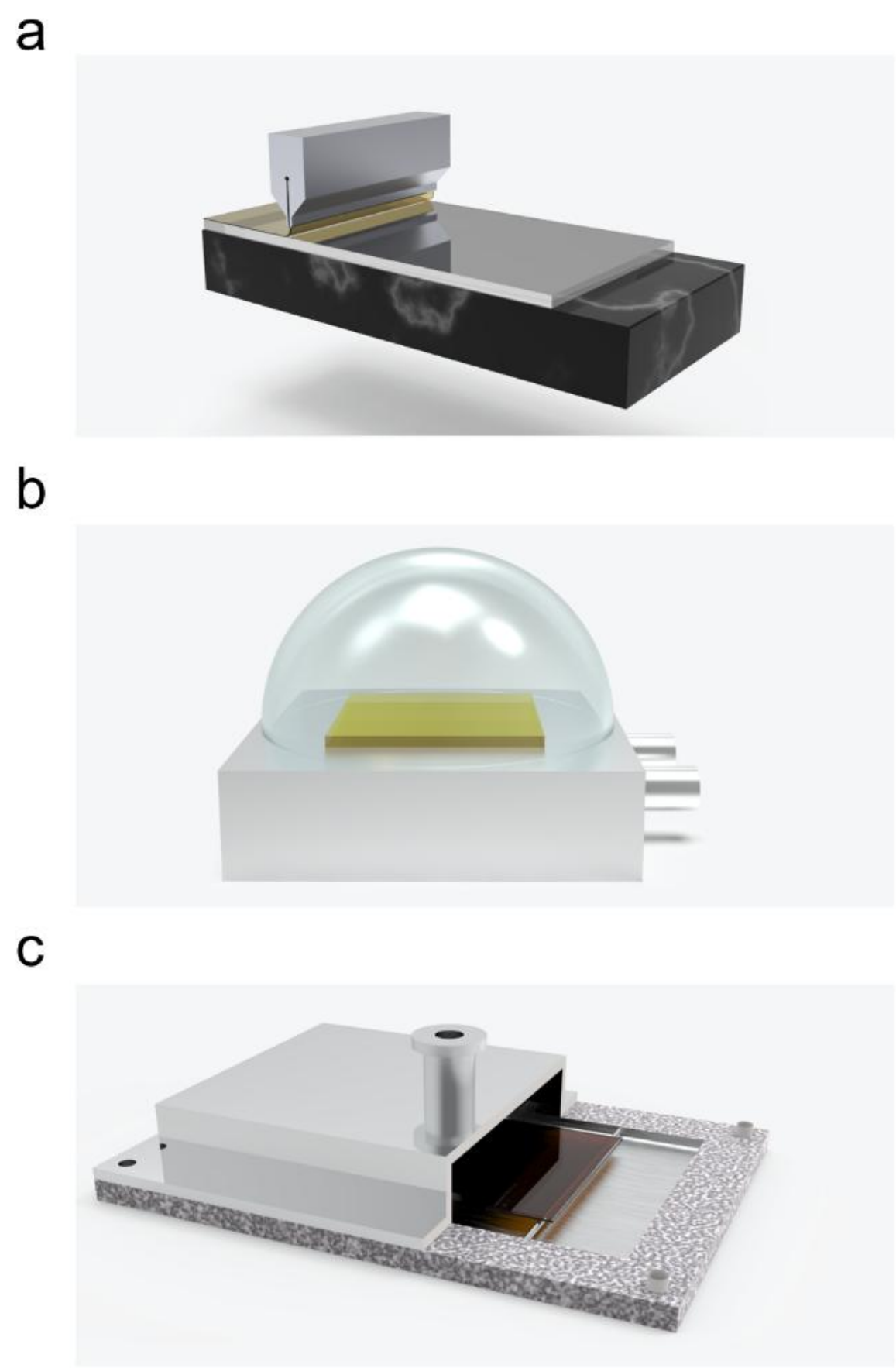

Supplementary Figure 2 | Processing routes used for perovskite-film formation. a, Slot-die coating. **b,** Cryo-controlled crystallization strategy. **c,** Vacuum flash.

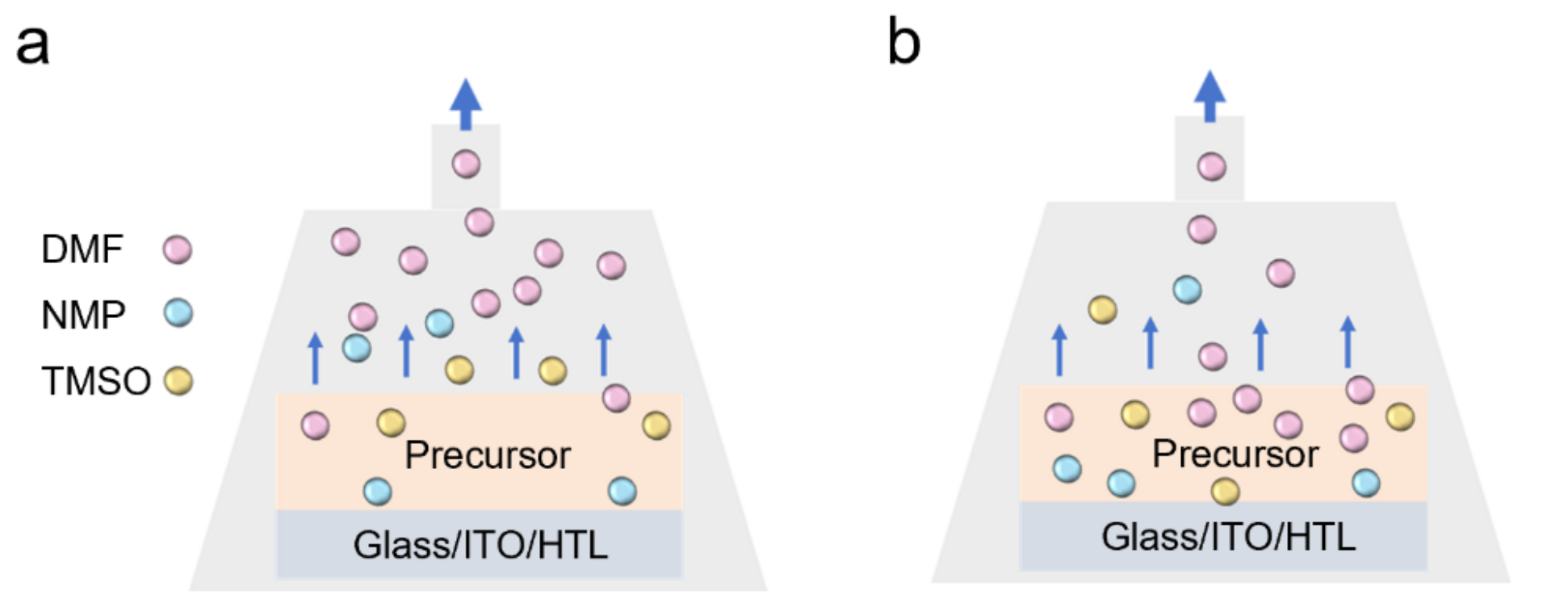

Supplementary Figure 3 | Schematic illustration of moderated solvent evaporation in perovskite precursor films. a, b, Schematic illustration of solvent evaporation in control (**a**) and target perovskite films (**b**).

a

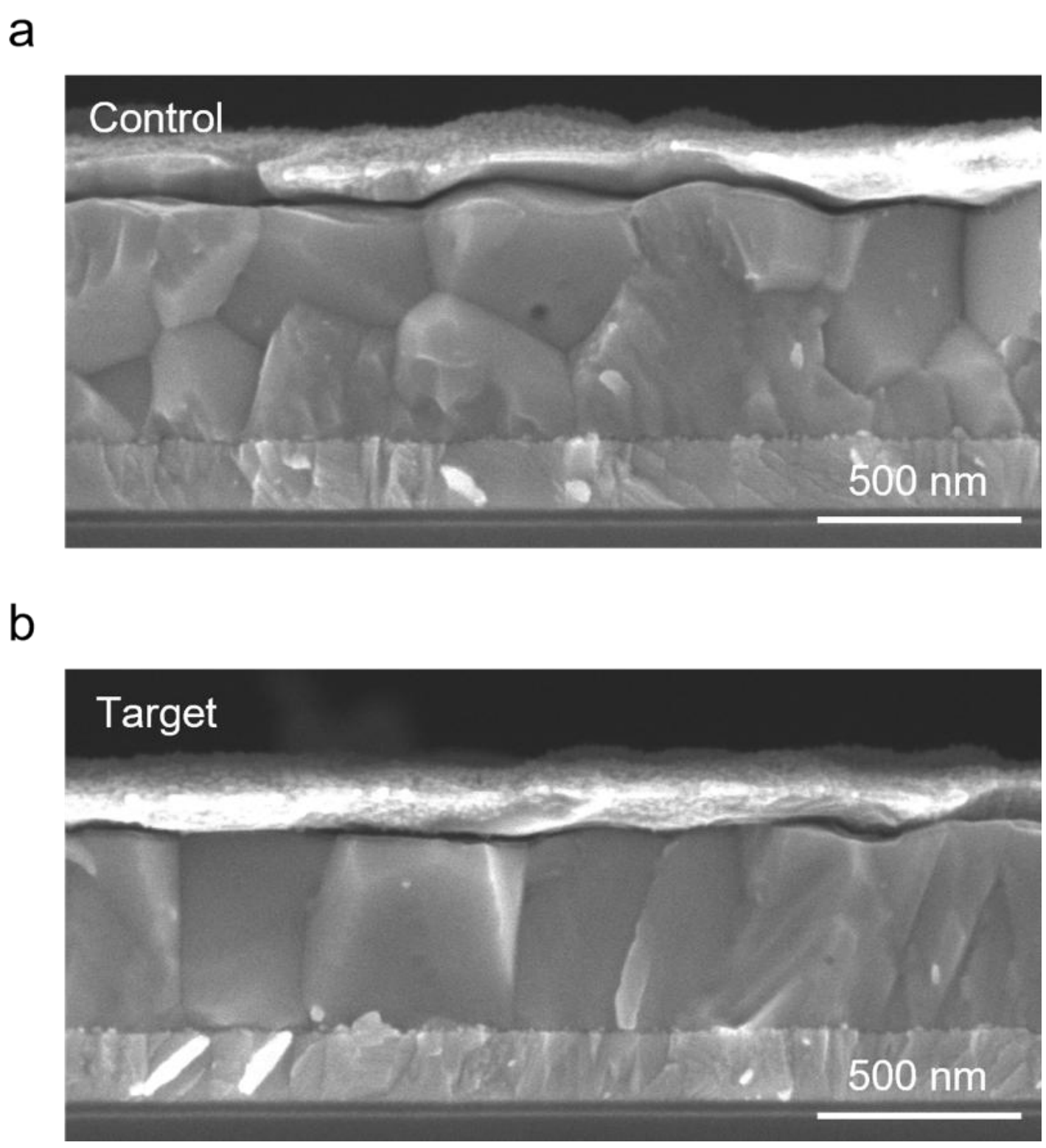

Supplementary Figure 4 | Cross-sectional scanning electron microscopy (SEM) images of perovskite devices. a, Control perovskite device. **b,** Target perovskite device. Scale bars, 500 nm.

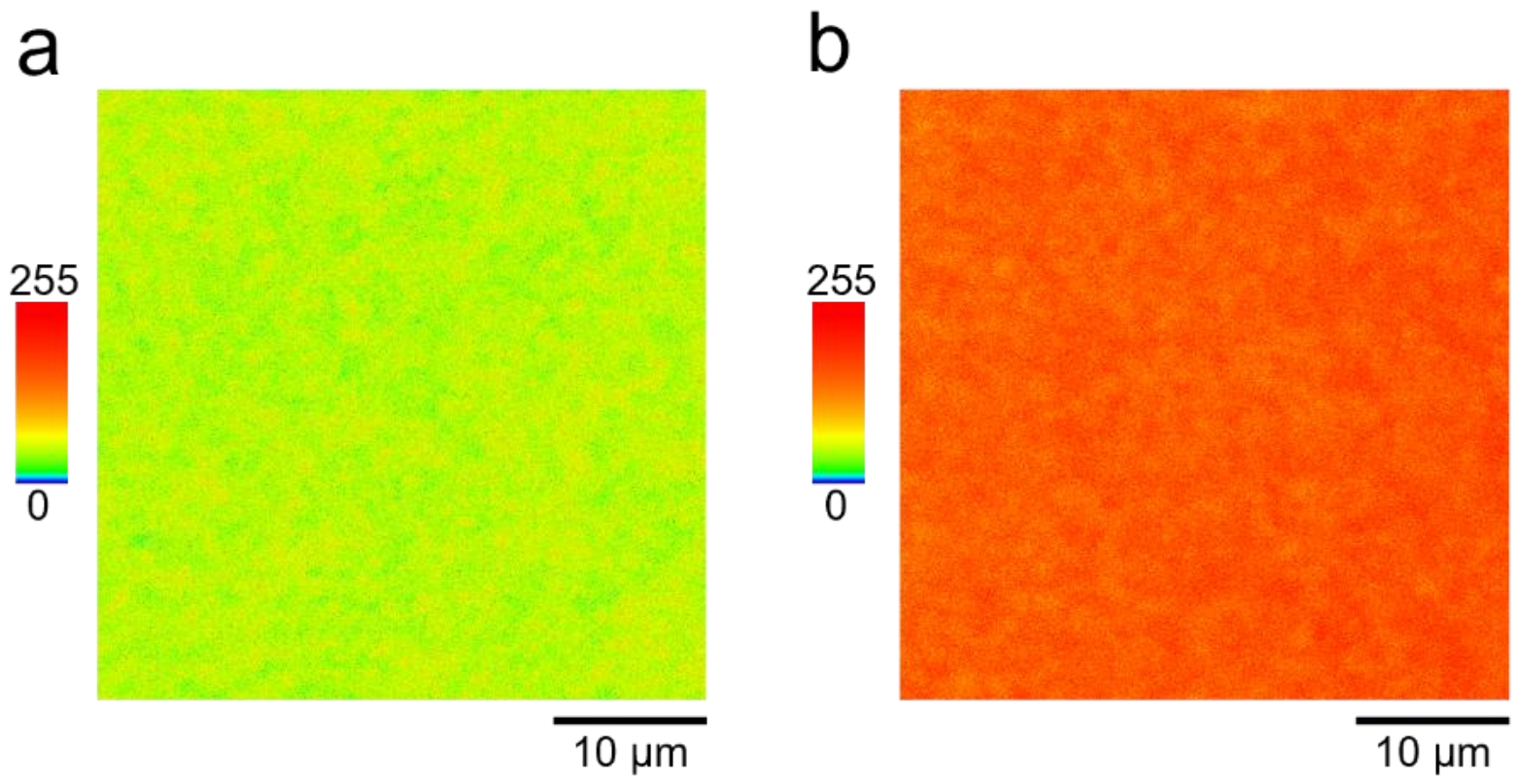

Supplementary Figure 5 | Spatial photoluminescence (PL) mapping of perovskite films.
a, Control perovskite films. **b,** Target perovskite films.

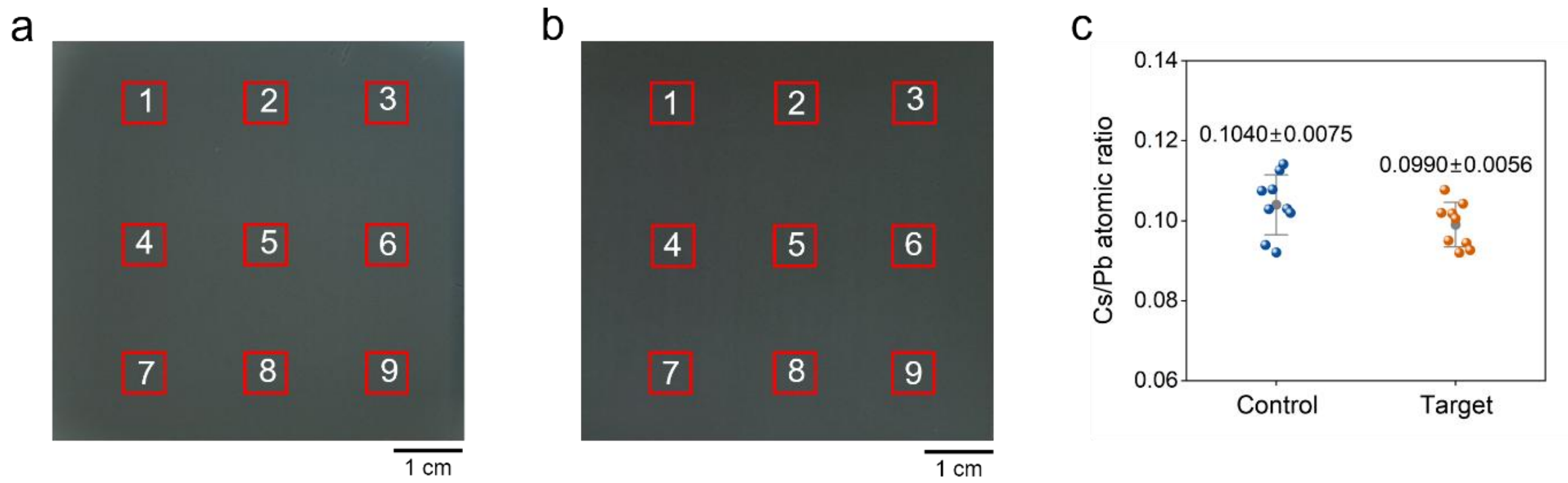

Supplementary Figure 6 | Spatial compositional uniformity of control and target perovskite films determined by energy-dispersive X-ray spectroscopy (EDS). a, b, Photographs of the control (**a**) and target (**b**) perovskite films showing the nine spatially distributed positions selected for EDS measurements. **c,** Cs/Pb atomic ratios obtained at the corresponding positions. Individual points represent measurements at different spatial locations, and grey symbols and error bars indicate the mean ± s.d. (N = 9 spatial positions).

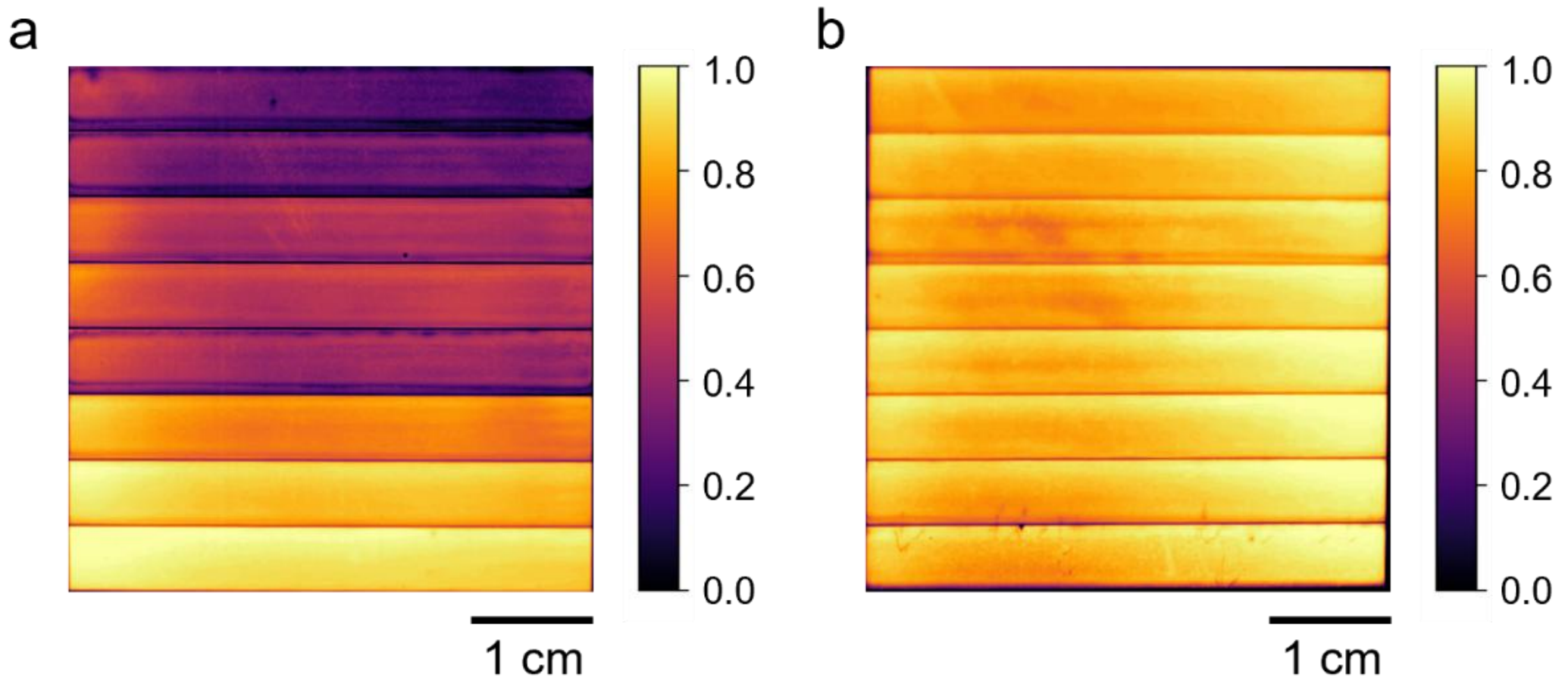

Supplementary Figure 7 | Spatial distribution of normalized electroluminescence (EL) intensity in perovskite minimodules. a, Control minimodule. **b,** Target minimodule.

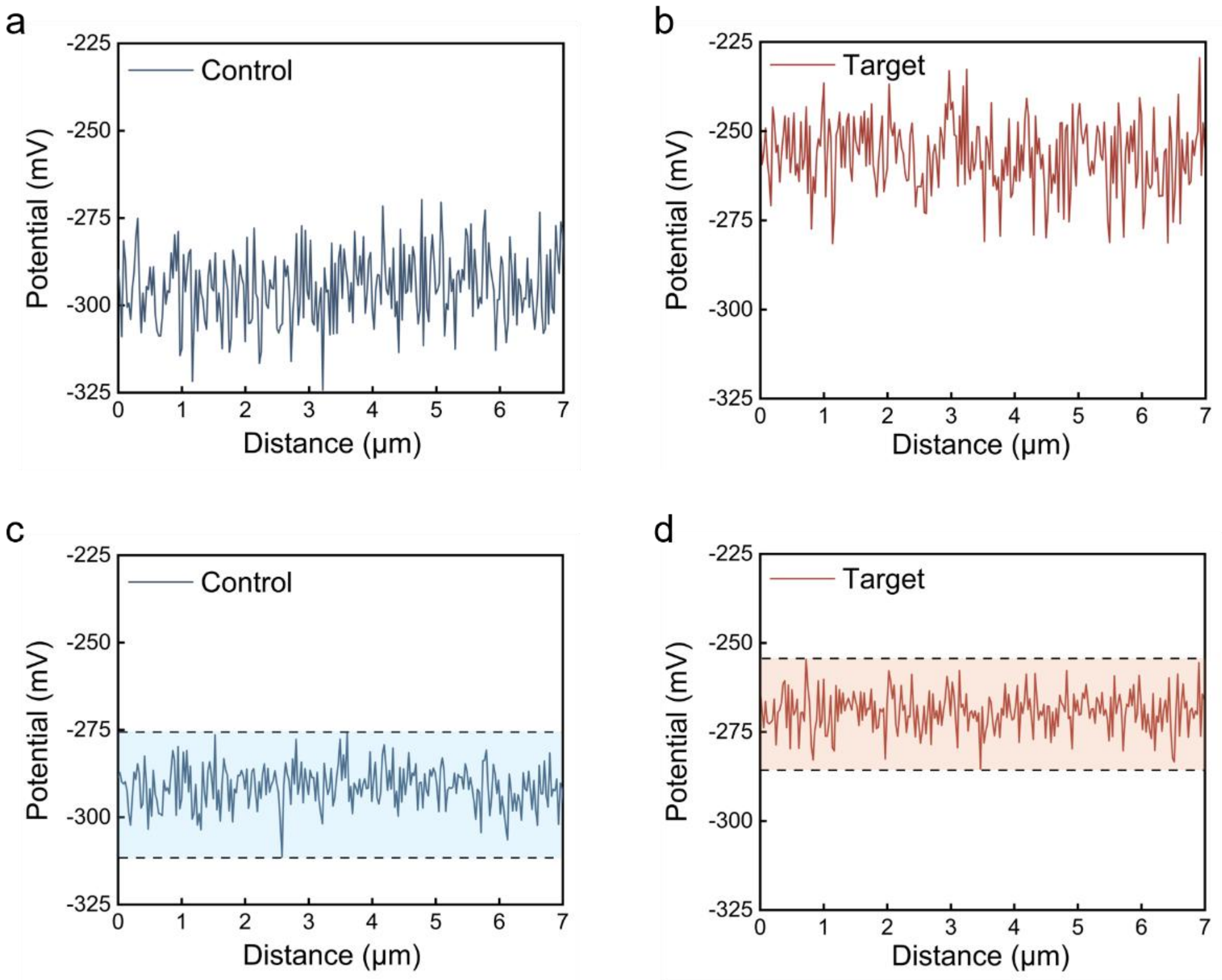

Supplementary Figure 8 | Kelvin probe force microscopy (KPFM) surface-potential profiles of the control and target perovskite films. a, b, Surface-potential line profiles extracted along the diagonal directions of the representative control (**a**) and target (**b**) KPFM maps shown in Fig. 3. **c, d,** Mean surface-potential profiles obtained by averaging line profiles measured at three spatially separated regions of the control (**c**) and target (**d**) films, respectively.

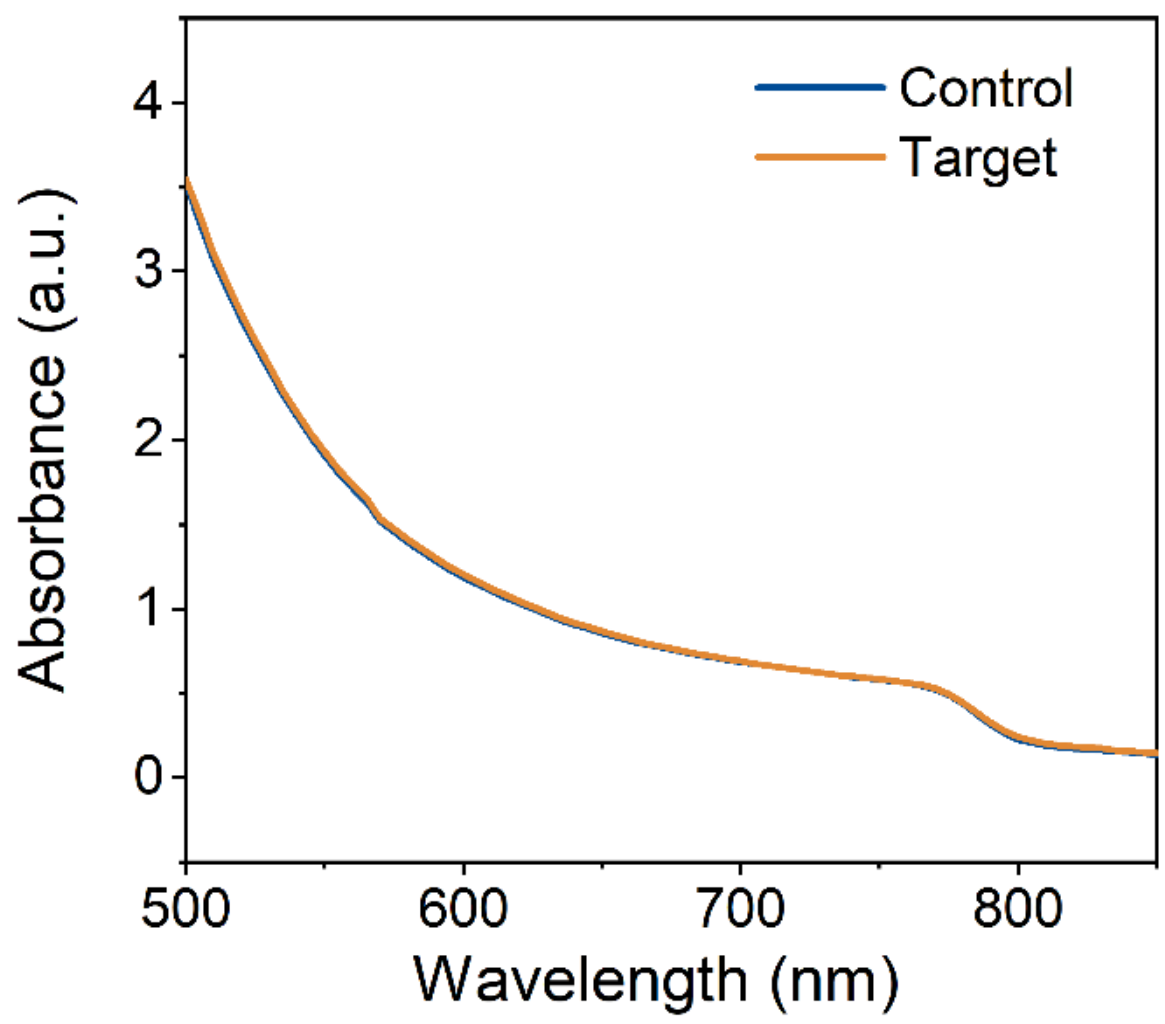

Supplementary Figure 9 | Ultraviolet–visible spectroscopy (UV–VIS) absorption spectra of control and target perovskite films.

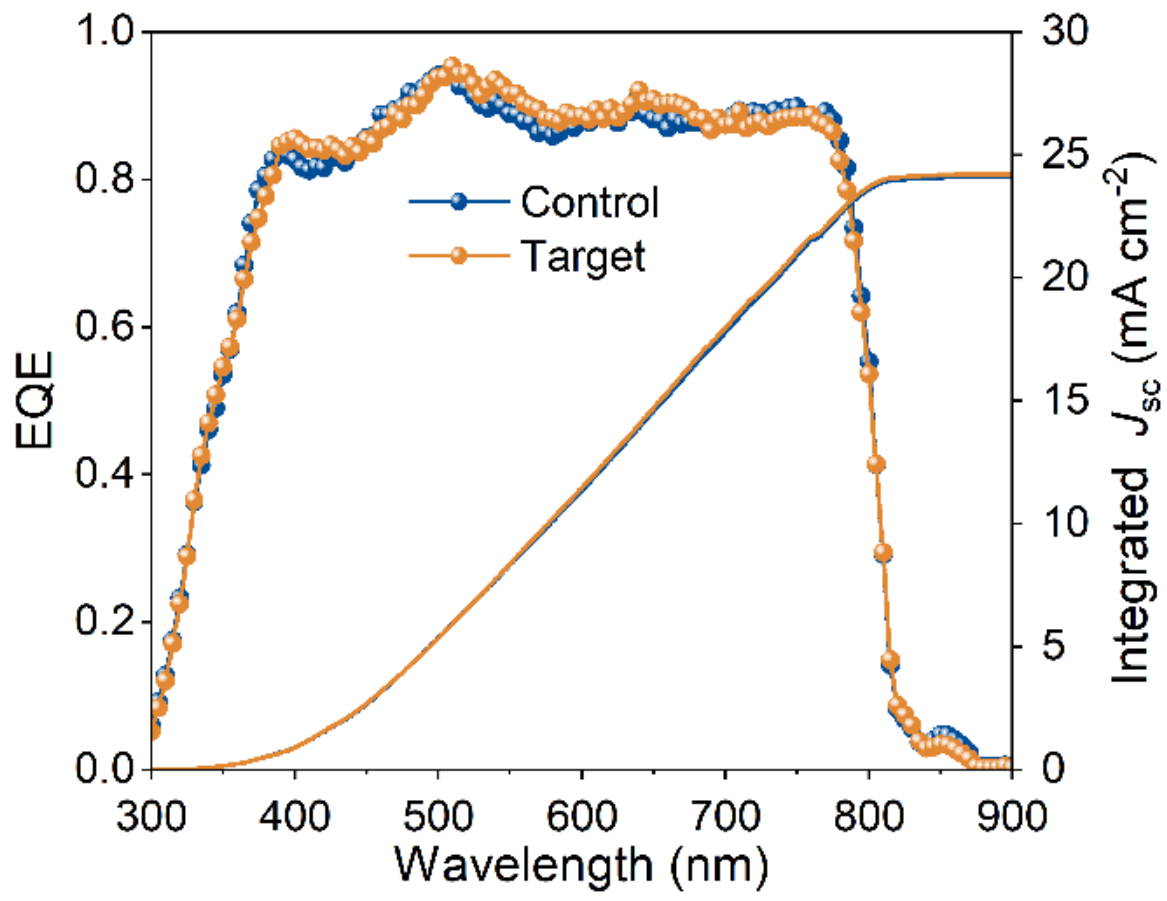

Supplementary Figure 10 | External quantum efficiency (EQE) spectra of individual sub-cells within the control and target mini-modules. The integrated J_{sc} values are 24.14 and 24.22 mA cm^{-2} for the control and target devices, respectively.

检测结果/说明：
Results of Test and additional explanation.

1. Standard Test Condition (STC)：Total Irradiance：1000 W/m^2
Temperature：25.0 °C
Spectral Distribution：AM1.5G

2.Measurement Data and I-V/P-V Curves under STC

Forward Scan

I_{sc} (mA)	V_{oc}(V)	I_{MPP}(mA)	V_{MPP}(V)	P_{MPP} (mW)	FF(%)	A (cm^2)
58.67	9.628	54.99	8.368	460.2	81.47	19.48

Reverse Scan

I_{sc} (mA)	V_{oc}(V)	I_{MPP}(mA)	V_{MPP}(V)	P_{MPP} (mW)	FF(%)	A (cm^2)
58.55	9.636	55.93	8.402	469.9	83.29	19.48

Mismatch factor:0.9966

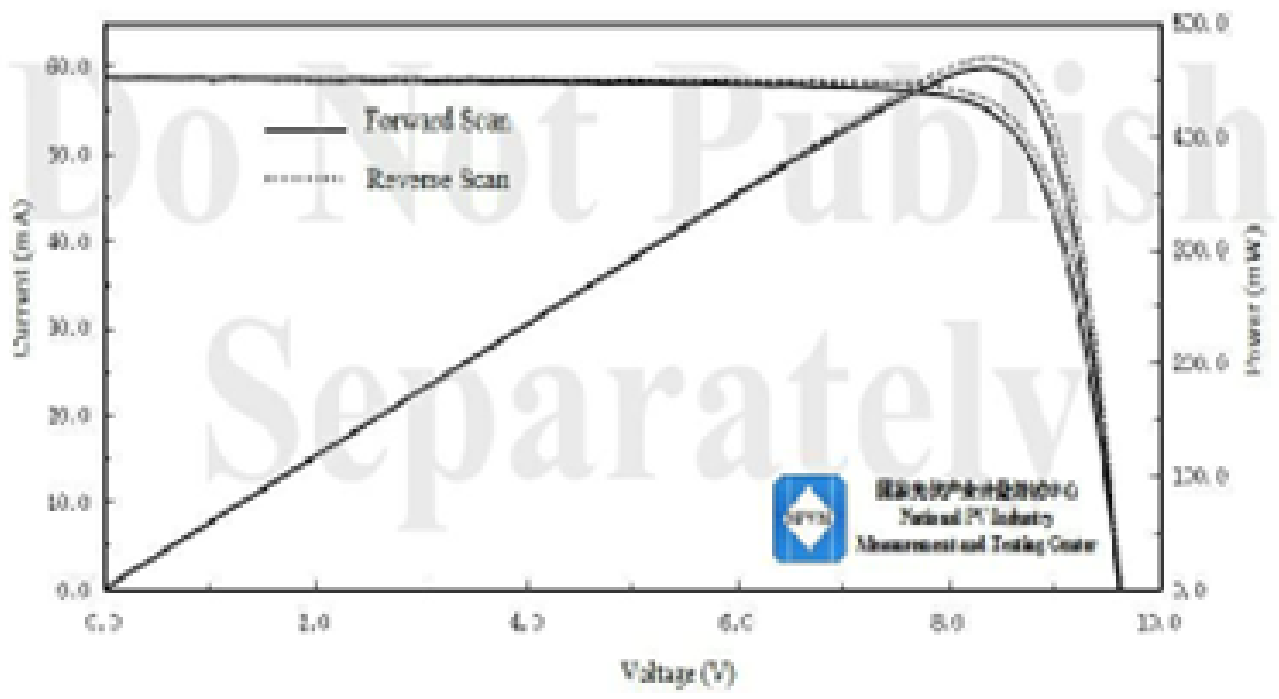

Figure 1. I-V and P-V characteristic curves of the measured sample under STC

福建省计量科学研究院
FUJIAN METROLOGY INSTITUTE
（国家光伏产业计量测试中心）
National PV Industry Measurement and Testing Center

报告编号： 25Q3-00275
Report No.

检测结果/说明：
Results of Test and additional explanation

3.Measurement Data and Curves for MPPT under STC

η (%)	23.93
P_{MPP} (mW)	466.2
I_{MPP} (mA)	55.34
V_{MPP} (V)	8.424

Note: Measurement data for MPPT under STC in the above table was the mean value acquired during the final 30 seconds of the 300 seconds test

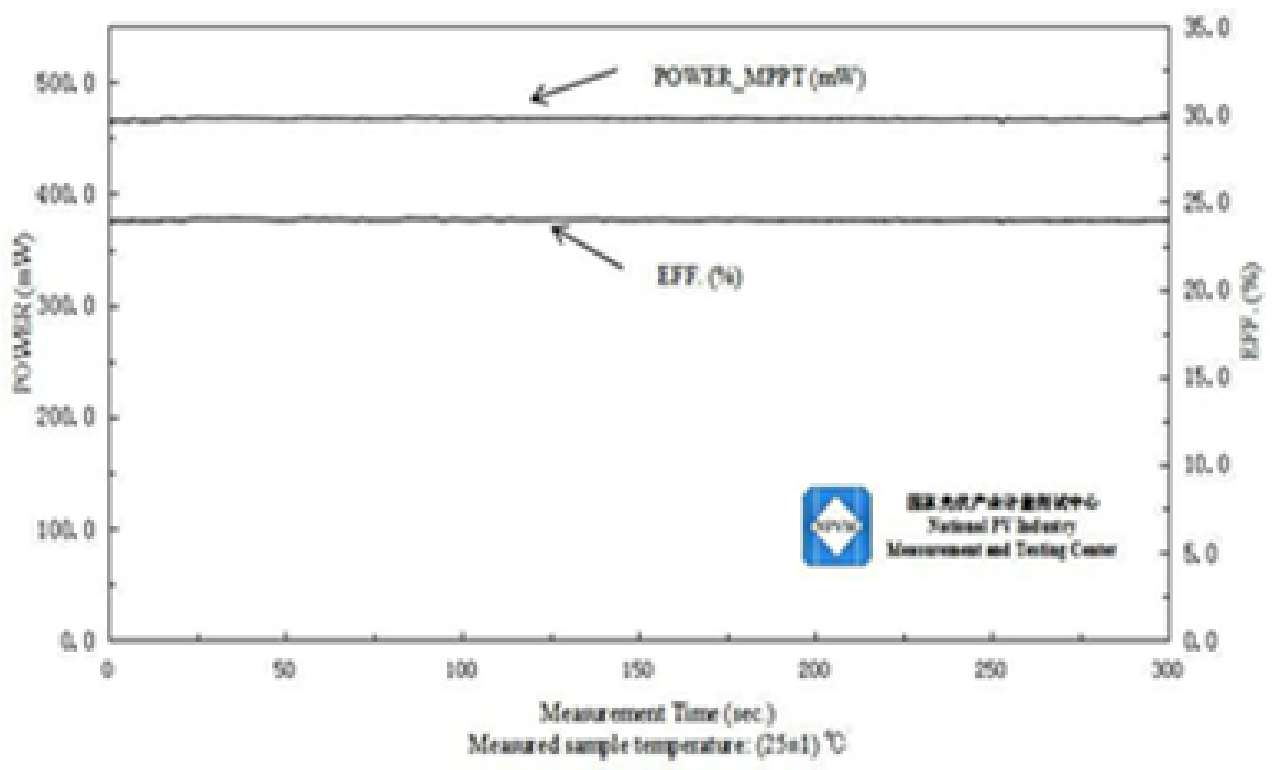

Figure 2. Measurement curves of the measured sample for MPPT

检测报告续页专用
Continued page of test report

Supplementary Figure 11 | Independent efficiency certification of the perovskite mini-module. Independent efficiency certification of perovskite mini-module by an accredited institute of National PV Industry Measurement and Testing Center (NPVM, China), giving a MPPT test efficiency of 23.93% with a 19.48 cm^2 aperture area.

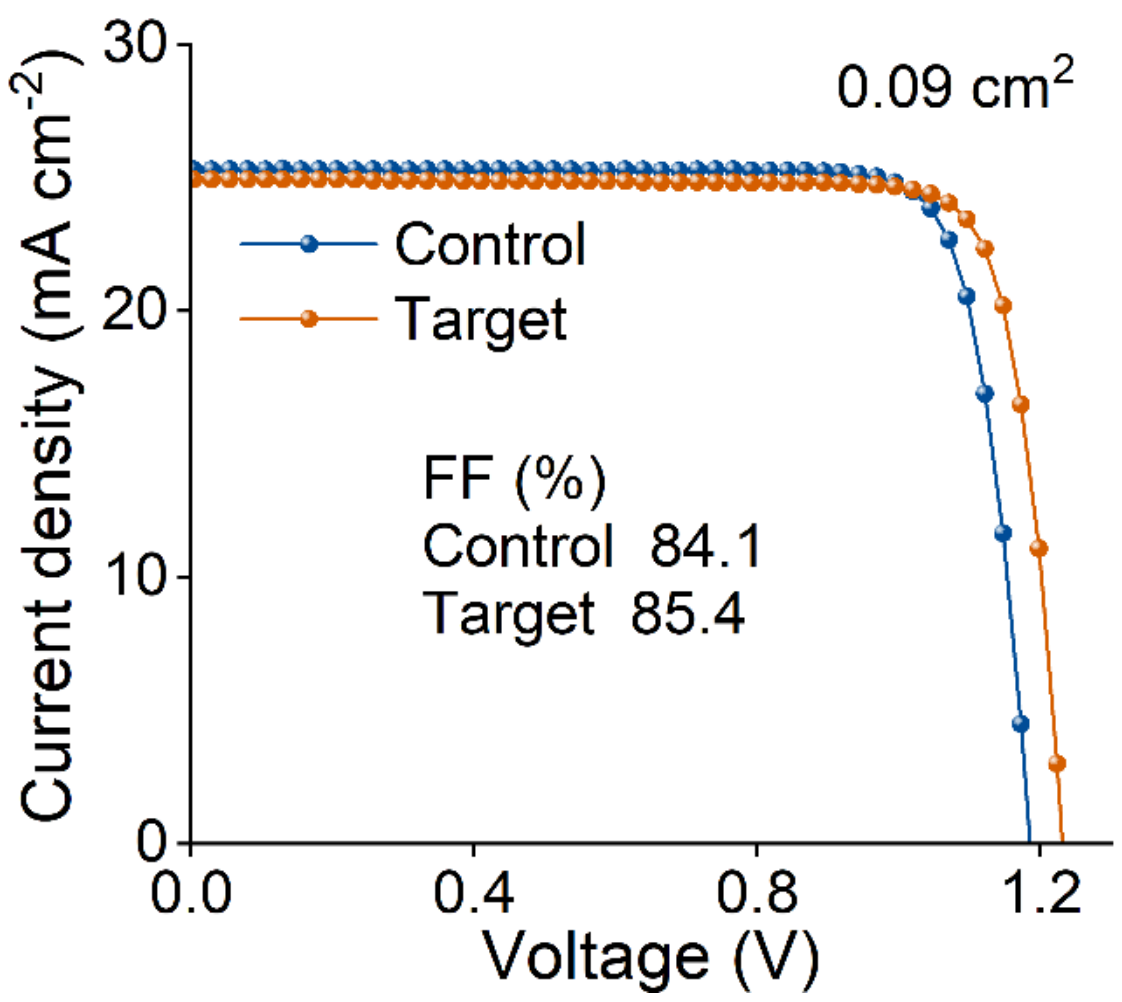

Supplementary Figure 12 | Current density–Voltage (*J–V*) characteristics of champion 0.09 cm^2 control and target PSCs. The corresponding photovoltaic performance parameters are provided in Supplementary Table 4.

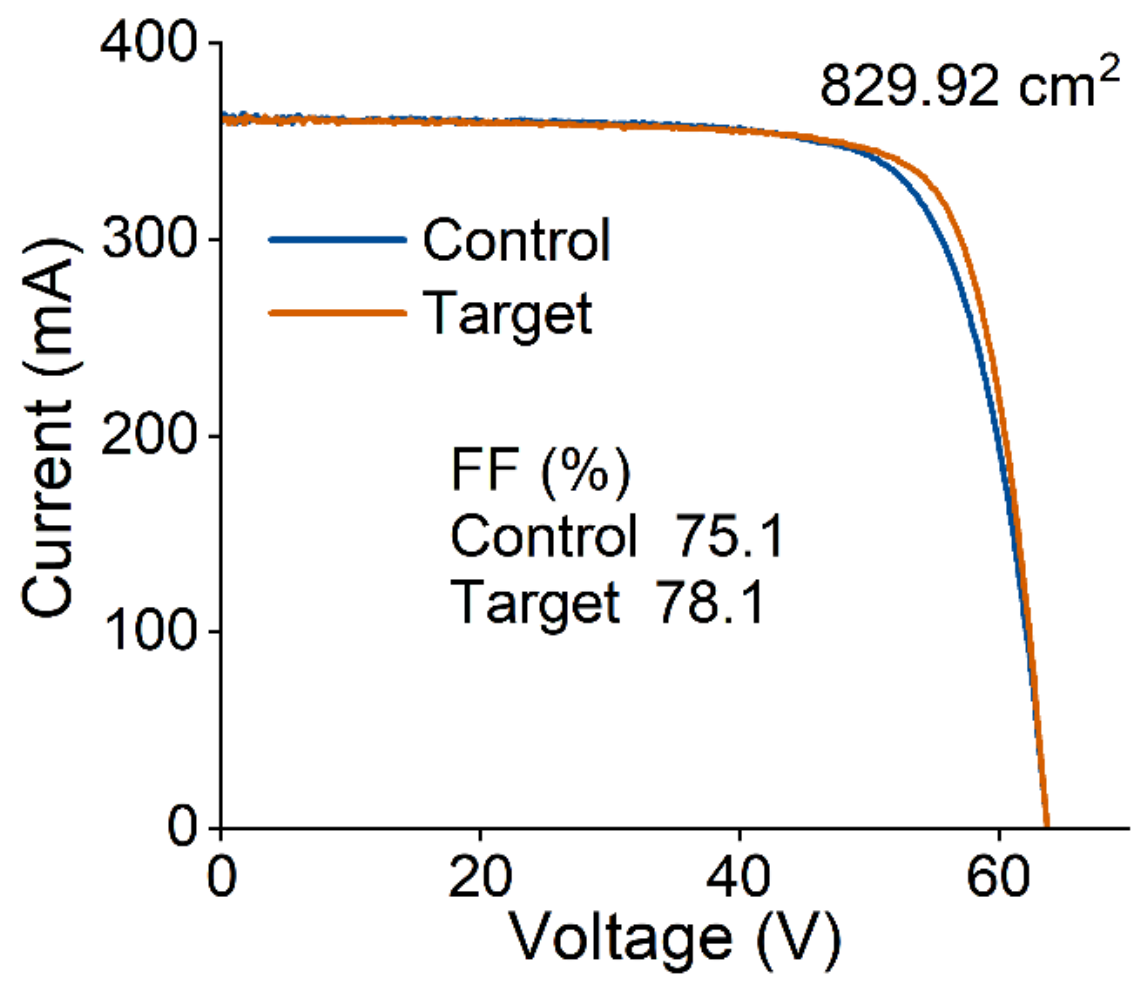

Supplementary Figure 13 | Current–Voltage (I–V) characteristics of 829.92 cm^2 control and target modules. The corresponding FF values are indicated in the plot.

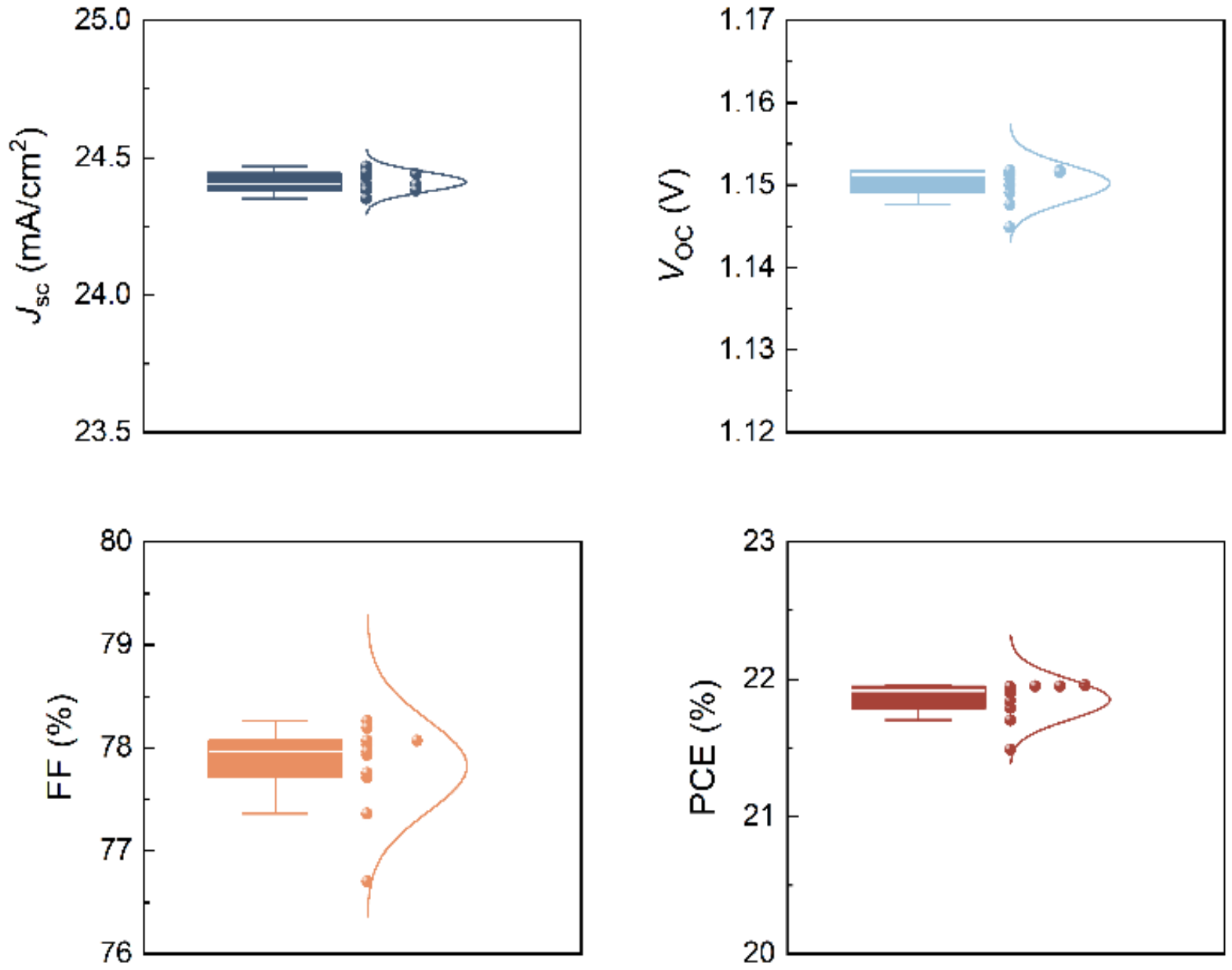

Supplementary Figure 14 | Statistical distributions of photovoltaic parameters for target 829.92 cm^2 modules with a 30 × 40 cm^2 substrate.

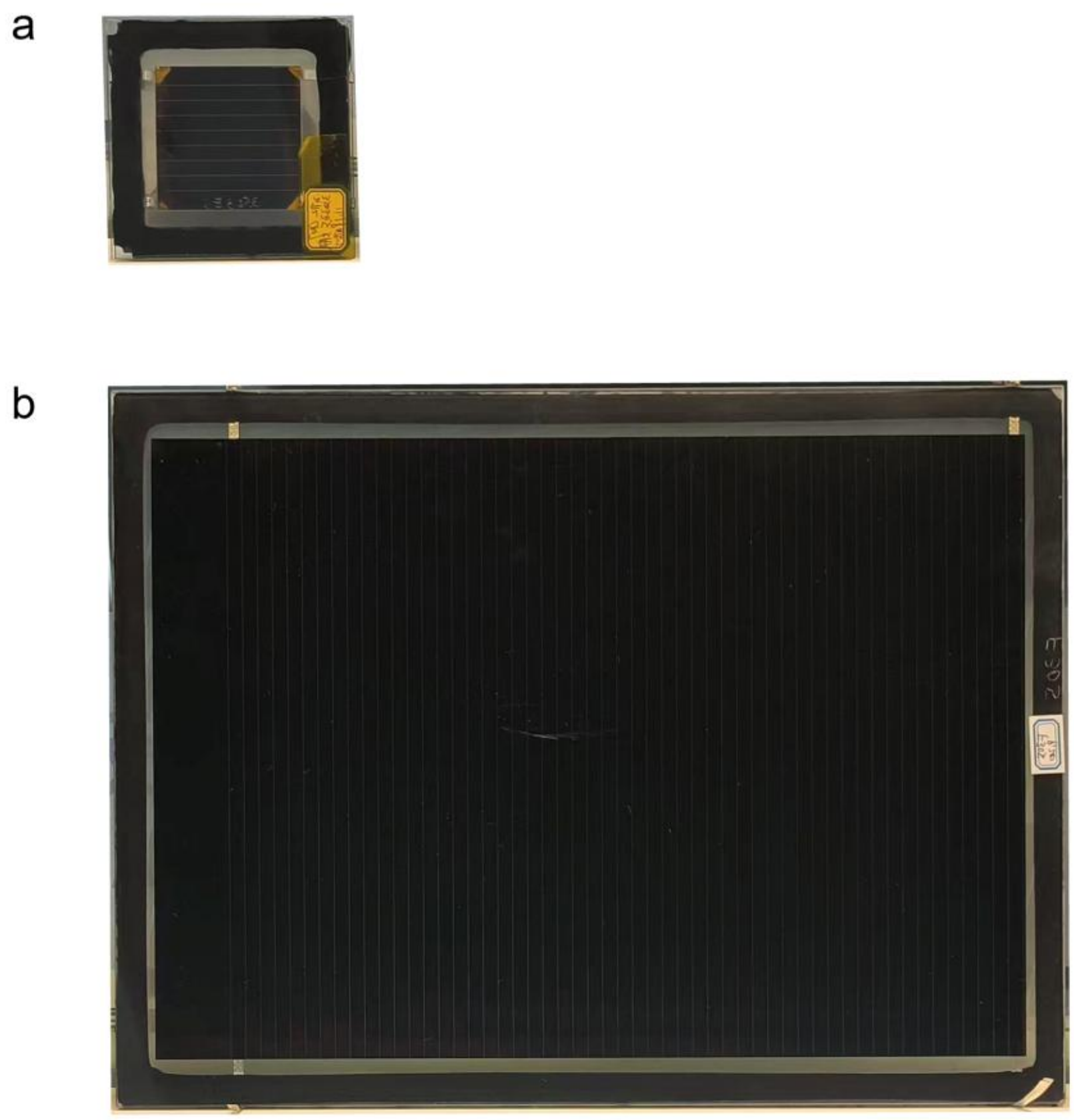

Supplementary Figure 15 | Photographs of fabricated perovskite devices across scales. a, 19.48 cm² aperture-area mini-module (eight series-connected cells). **b**, 829.92 cm² aperture-area module (fifty-six series-connected cells).

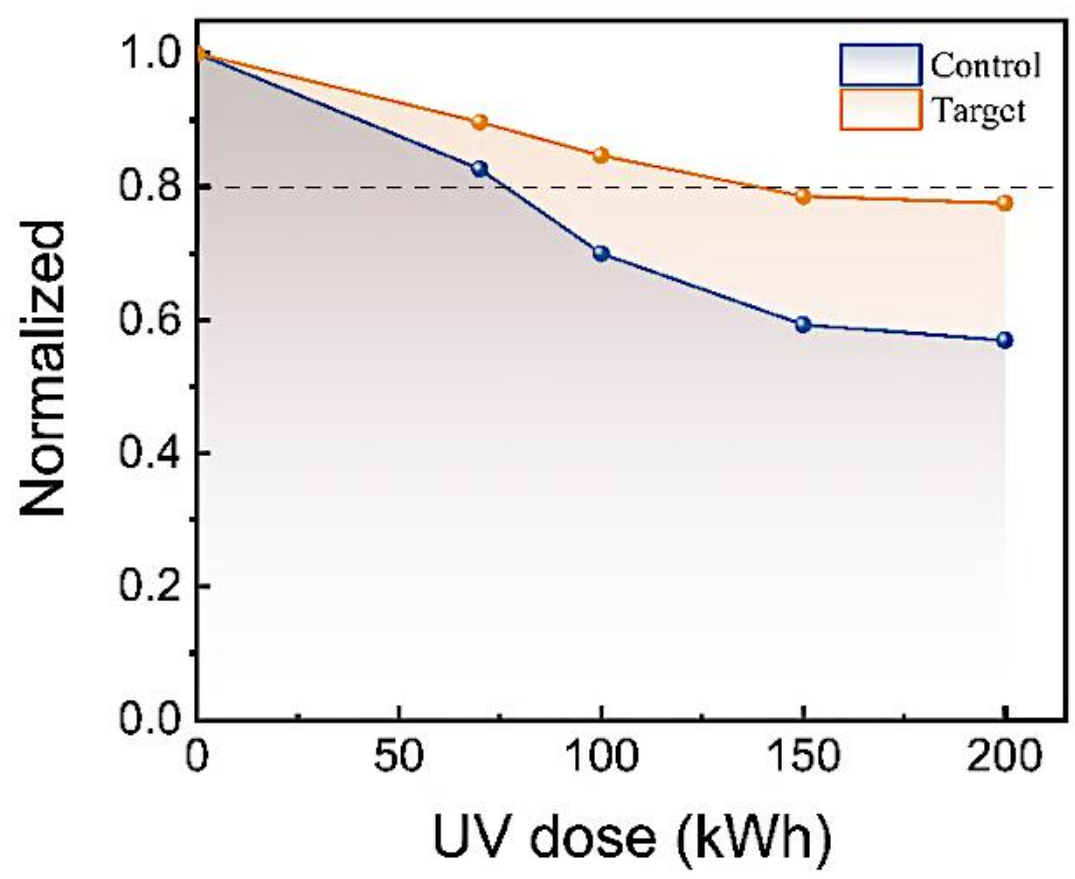

Supplementary Figure 16 | Evolution of normalized efficiency for encapsulated control and target perovskite modules under ultraviolet (UV) ageing.

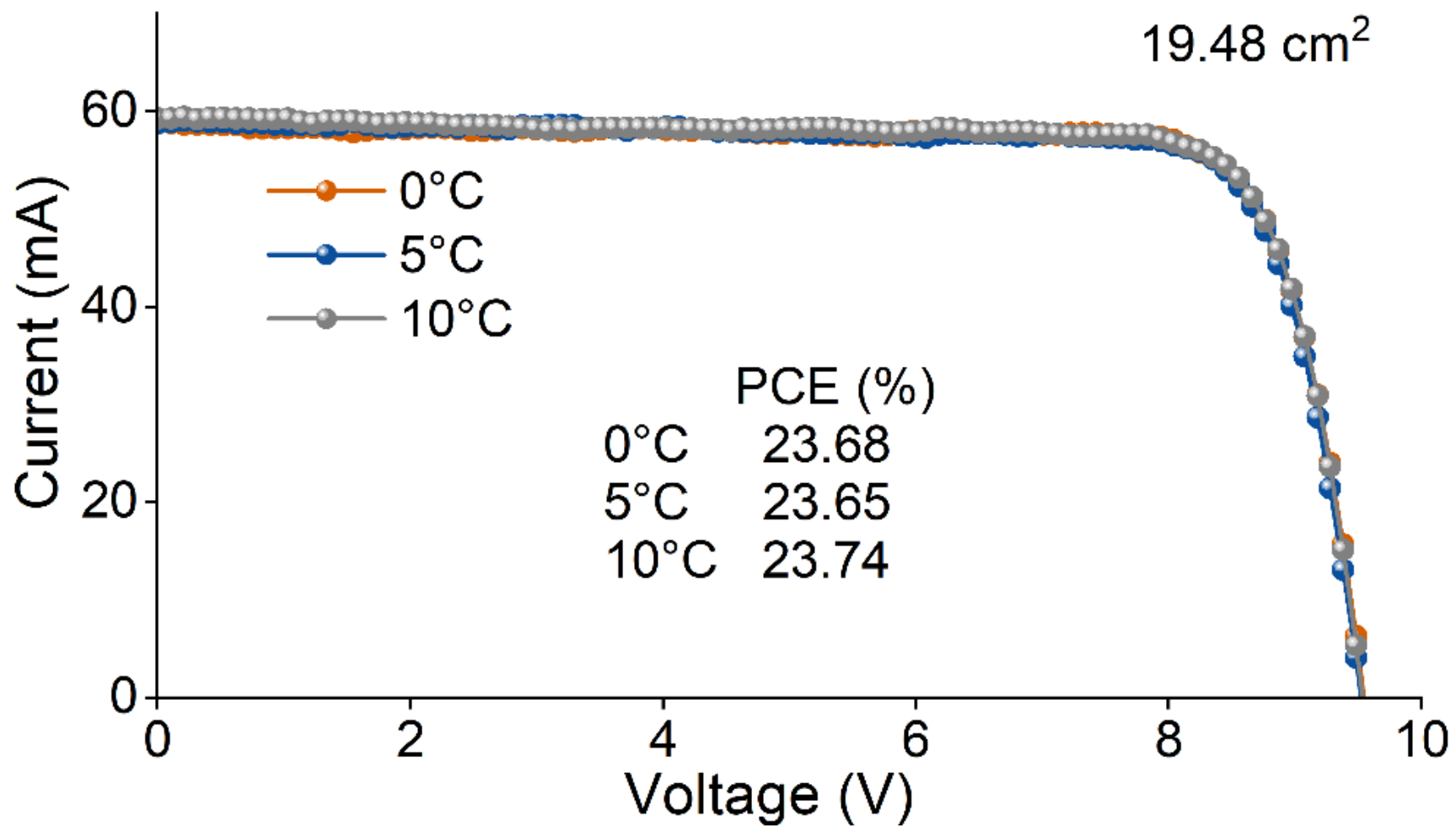

Supplementary Figure 17 | Effect of film temperature before vacuum flashing on device performance. Current–voltage (*I*–*V*) characteristics of 19.48 cm^2 perovskite minimodules processed at film temperatures of 0, 5 and 10 °C immediately before vacuum flashing. The corresponding PCEs are 23.68%, 23.65% and 23.74%, respectively.

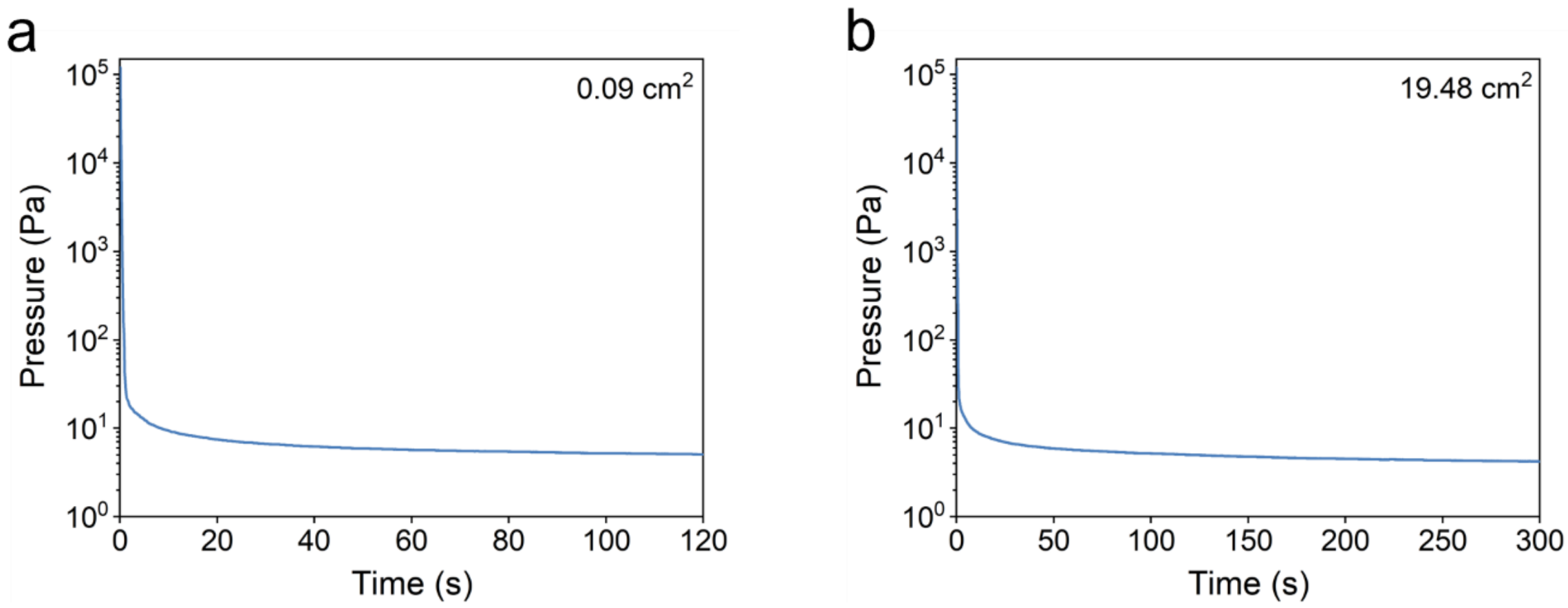

Supplementary Figure 18 | Pressure–time profiles during the vacuum-flash process. a, Pressure evolution during vacuum flashing for the 0.09 cm^2 device, with a total treatment duration of 120 s. **b**, Pressure evolution during vacuum flashing for the 19.48 cm^2 mini-module, with a total treatment duration of 300 s.

Supplementary Table 1 | Statistics of FF and PCE for perovskite devices of varying areas. The FF data are represented in Fig. 1c.

Area (cm^2)	FF (%)	PCE (%)	Ref.
0.04	87	25.5	10
0.05	84.5	26.7	11
0.06	84	25.21	12
0.07	85.5	26.5	13
0.08	84.2	25.8	14
0.09	82.9	25.3	15
0.09	84.3	26.1	16
0.09	85.4	26.5	17
0.09	86.2	25.9	18
0.1	82.8	24.9	19
0.1	83.3	25.6	20
0.1	82.5	26.1	21
1	79.5	24.88	22
1	83.32	25.26	23
1.03	82	25.2	24
11.46	82.1	23.4	20
11.7	81	22.7	10
12.4	79.6	23.3	15
12.96	78.6	22.7	16
12.96	78.4	23	17
14.61	79.5	22.7	19

19.48	83.3	23.9	This work
20.25	77.4	22.6	11
20.25	80.5	23.2	24
20.8	78.5	22.9	13
22.9	80.7	23.5	14
27.22	80.5	22.8	12
30.03	77.2	22	25
31	79.89	21.23	26
57.2	81.2	22.8	21
70	78.32	21.78	27
87.45	76.51	21.45	28
164	77.59	21.86	29
214.7	78.2	19.9	30
215.53	78.3	20.6	24
715.1	81.2	22.4	18
728	79.3	22.9	13
749.28	76.17	20.21	28
813.8	78.8	22.1	31
1027	77.1	19.2	24
7200	76	19.3	31
7906	76.5	15	11
20000	74.3	19	32

Supplementary Table 2 | Photovoltaic parameters of 19.48 cm^2 control and target perovskite mini-modules. Values are presented as mean ± s.d. from 15 devices measured under AM1.5G illumination (100 mW cm^{-2}).

	V_{OC} (V)	J_{SC} (mA cm^{-2})	FF (%)	PCE (%)
Control	1.16 ± 0.005	24.2 ± 0.14	79.24 ± 0.97	22.3 ± 0.28
Target	1.19 ± 0.004	24.4 ± 0.14	82.19 ± 0.71	23.9 ± 0.15

Supplementary Table 3 | Photovoltaic parameters of 829.92 cm^2 control and target perovskite modules measured under AM1.5G illumination (100 mW cm^{-2}).

	V_{OC} (V)	J_{SC} (mA cm^{-2})	*FF* (%)	PCE (%)
Control	1.15	24.0	75.1	20.7
Target	1.17	24.1	78.1	22.0

Supplementary Table 4 | Photovoltaic performance of the 0.09 cm^2 control and target perovskite solar cells shown in Supplementary Fig. 12, measured under AM1.5G illumination (100 mW cm^{-2}).

	V_{OC} (V)	J_{SC} (mA cm^{-2})	*FF* (%)	PCE (%)
Control	1.18	25.2	84.1	25.1
Target	1.22	24.8	85.4	25.8

Supplementary Table 5 | Photovoltaic performance of 0.09 cm^2 control and target perovskite cells. Values are presented as mean ± s.d. for 10 devices measured under AM1.5G illumination (100 mW cm^{-2}).

	V_{OC} (V)	J_{SC} (mA cm^{-2})	FF (%)	PCE (%)
Control	1.197 ± 0.014	24.95 ± 0.26	83.22 ± 0.89	24.8 ± 0.22
Target	1.220 ± 0.013	25.05 ± 0.23	83.64 ± 0.77	25.6 ± 0.28

References

1 De Boer, P. Additive Structural Decomposition Analysis and Index Number Theory: An Empirical Application of the Montgomery Decomposition. *Economic Systems Research* **20**, 97-109, doi:10.1080/09535310801892066 (2008).

2 Shorrocks, A. F. Decomposition procedures for distributional analysis: a unified framework based on the Shapley value. *The Journal of Economic Inequality* **11**, 99-126, doi:10.1007/s10888-011-9214-z (2012).

3 Green, M. A. Accuracy of analytical expressions for solar cell fill factors. *Solar Cells* **7**, 337-340, doi:10.1016/0379-6787(82)90057-6 (1982).

4 Green, M. A. Accurate expressions for solar cell fill factors including series and shunt resistances. *Applied Physics Letters* **108**, doi:10.1063/1.4942660 (2016).

5 Shockley, W. & Queisser, H. J. Detailed Balance Limit of Efficiency of p-n Junction Solar Cells. *Journal of Applied Physics* **32**, 510-519, doi:10.1063/1.1736034 (1961).

6 Rau, U. Reciprocity relation between photovoltaic quantum efficiency and electroluminescent emission of solar cells. *Physical Review B* **76**, doi:10.1103/PhysRevB.76.085303 (2007).

7 Caprioglio, P. *et al.* On the Origin of the Ideality Factor in Perovskite Solar Cells. *Advanced Energy Materials* **10**, doi:10.1002/aenm.202000502 (2020).

8 Calado, P. *et al.* Identifying Dominant Recombination Mechanisms in Perovskite Solar Cells by Measuring the Transient Ideality Factor. *Physical Review Applied* **11**, doi:10.1103/PhysRevApplied.11.044005 (2019).

9 Yuan, Y. *et al.* Shallow defects and variable photoluminescence decay times up to 280 micros in triple-cation perovskites. *Nat Mater* **23**, 391-397, doi:10.1038/s41563-023-01771-2 (2024).

10 Bi, L. *et al.* Temperature-controlled vacuum quenching for perovskite solar modules towards scalable production. *Nature Photonics* **19**, 968-976, doi:10.1038/s41566-025-01703-3 (2025).

11 Green, M. A. *et al.* Solar cell efficiency tables (Version 64). *Progress in Photovoltaics: Research and Applications* **32**, 425-441, doi:10.1002/pip.3831 (2024).

12 Ding, B. *et al.* Dopant-additive synergism enhances perovskite solar modules. *Nature* **628**, 299-305, doi:10.1038/s41586-024-07228-z (2024).

13 Jin, L. *et al.* Suppressing solvent adducts via coordination competition enables scalable perovskite photovoltaics. *Nat Commun* **17**, 1737, doi:10.1038/s41467-026-68439-8 (2026).

14 Huang, C. *et al.* Buried Interface Modification for Efficient FAPbI3 Perovskite Modules by Full Blade-Coating Process. *Advanced Energy Materials* **15**, doi:10.1002/aenm.202501227 (2025).

15 Huang, C. *et al.* Meniscus-modulated blade coating enables high-quality α-phase formamidinium lead triiodide crystals and efficient perovskite minimodules. *Joule* **8**, 2539-2553, doi:10.1016/j.joule.2024.06.008 (2024).

16 Yang, J. *et al.* Crystal phase and band edge modulation of MA- and Br-free CsFA-based perovskites for efficient inverted solar cells and minimodules. *Energy & Environmental Science* **18**, 1732-1744, doi:10.1039/d4ee05860g (2025).

17 Xu, P. *et al.* Retarding the Growth Kinetics of Chemical Bath Deposited Nickel Oxide Films for Efficient Inverted Perovskite Solar Cells and Minimodules. *Adv Mater* **37**, e2505087, doi:10.1002/adma.202505087 (2025).

18 Wang, H. *et al.* Impurity-healing interface engineering for efficient perovskite submodules. *Nature* **634**, 1091-1095, doi:10.1038/s41586-024-08073-w (2024).

19 Zhou, H. *et al.* Efficient and stable perovskite mini-module via high-quality homogeneous perovskite crystallization and improved interconnect. *Nat Commun* **15**, 6679, doi:10.1038/s41467-024-50962-1 (2024).

20 Xiao, G. B. *et al.* Achieving >23% Efficiency Perovskite Solar Minimodules with Surface Conductive Coordination Polymer. *Adv Mater* **37**, e2407225, doi:10.1002/adma.202407225 (2025).

21 Zhu, X. *et al.* Interfacial molecular anchor for ambient all-bladed perovskite solar modules. *Joule* **9**, doi:10.1016/j.joule.2025.101919 (2025).

22 Zhou, J. *et al.* Highly efficient and stable perovskite solar cells via a multifunctional hole transporting material. *Joule*, doi:10.1016/j.joule.2024.02.019 (2024).

23 Ouyang, B. *et al.* Robust 3D/2D heterojunction with oriented dion-jacobson layer for improved ion migration suppression in large-area inverted perovskite solar cells. *Nano Energy* **140**, doi:10.1016/j.nanoen.2025.111024 (2025).

24 Green, M. A. *et al.* Solar Cell Efficiency Tables (Version 65). *Progress in Photovoltaics: Research and Applications* **33**, 3-15, doi:10.1002/pip.3867 (2024).

25 Wang, F. *et al.* A Polymeric Hole Transporter with Dual-Interfacial Interactions Enables 25%-Efficiency Blade-Coated Perovskite Solar Cells. *Adv Mater* **36**, e2412059, doi:10.1002/adma.202412059 (2024).

26 Yoo, J. W. *et al.* R4N+ and Cl− stabilized α-formamidinium lead triiodide and efficient bar-coated mini-

modules. *Joule* **7**, 797-809, doi:10.1016/j.joule.2023.03.003 (2023).
27 Liu, H. *et al.* Simultaneous mechanical and chemical synthesis of long-range-ordered perovskites. *Nature Synthesis* **4**, 196-208, doi:10.1038/s44160-024-00687-2 (2024).
28 Sun, Y. *et al.* In situ coordinated HTL strategy for high-performance and scalable perovskite solar cells. *Nat Commun* **16**, 9110, doi:10.1038/s41467-025-64111-9 (2025).
29 Chen, M. *et al.* Reinforced Anchor for Scalable Self-Assembled Monolayer to Attain High-Performance Perovskite Solar Modules. *Advanced Energy Materials* **15**, doi:10.1002/aenm.202502000 (2025).
30 Yun, H. S. *et al.* Surface Engineering of Tin Oxide Nanoparticles by pH Modulation Facilitates Homogeneous Film Formation for Efficient Perovskite Solar Modules. *Advanced Energy Materials* **14**, doi:10.1002/aenm.202400791 (2024).
31 Green, M. A. *et al.* Solar cell efficiency tables: Version 68. *Joule*, doi:10.1016/j.joule.2026.102494 (2026).
32 Wang, Z. *et al.* Manipulating the crystallization kinetics of halide perovskites for large-area solar modules. *Communications Materials* **5**, doi:10.1038/s43246-024-00566-5 (2024).
