# Supplementary material for: Retarding efficiency loss upon scaling in perovskite photovoltaics as fill factor becomes the bottleneck
Source: Nat Commun. 2026 Oct 29;17:11641. doi: 10.1038/s41467-026-78867-1 (PMC13760041; doi:10.1038/s41467-026-78867-1)
Supplement: Supplementary file 2 [file 41467_2026_78867_MOESM2_ESM.pdf]

nature portfolio

Peer Review File

Retarding efficiency loss upon scaling in perovskite photovoltaics as fill factor becomes the bottleneck

Corresponding Author: Professor Dongchen Lan

This file contains all reviewer reports in order by version, followed by all author rebuttals in order by version.

Version 0:

Reviewer comments:

Reviewer #1

(Remarks to the Author)
This manuscript proposes a strategy to preserve the fill factor (FF) of perovskite solar modules by controlling the crystallization at low temperature. By prolonging the evolution of the intermediate phase, improving the crystallization quality of the perovskite films, and suppressing recombination losses, the resulting minimodule with an aperture area of 19.48 cm^2 achieves an impressing result with a certified efficiency of 23.9%. However, this work fails to demonstrate substantial innovation beyond existing literature. Similar approaches have already been reported in previous studies (Nat. Commun. 15, 6679, 2024; Energy Environ. Sci. 18, 10434-10443, 2025), in which low temperature was used to regulate intermediate-phase evolution, solvent evaporation, and perovskite crystallization. Besides, the central opinion using FF as a key descriptor for the efficiency loss and operation stability is not convincing, as FF is strongly connected with other parameters. Therefore, I cannot suggest its publication on Nature Communications.

Reviewer #2

(Remarks to the Author)
Comments

This manuscript focuses on FF preservation during the scale-up of perovskite photovoltaic devices from small-area cells to mini-modules. The authors propose a cryo-controlled processing strategy to regulate the evolution of the intermediate phase during crystallization, thereby improving film formation and reducing FF losses. Overall, this processing strategy appears to be effective in improving FF, device reproducibility, and stability. The mini-module efficiency and FF are also quite good, which is a clear strength of the work. Another interesting point is that this process seems to mainly improve FF, while having only a limited effect on the other photovoltaic parameters. Such selective FF enhancement is relatively uncommon and provides a useful entry point for studying the origin of FF losses.
However, I still have several concerns about the mechanistic interpretation. In particular, the authors argue that the FF loss during scale-up mainly originates from recombination-related losses, and further attribute the FF improvement from cryo-controlled processing mainly to recombination suppression. At present, the discussion of the ideality factor n_id is not sufficiently clear, and some interpretations appear to be somewhat inconsistent. I think these issues should be clarified before publication and would require substantial revision.

1. In the introduction and background sections, the authors emphasize that one of the highlights of this work is to show that FF is the dominant loss during the scale-up of perovskite photovoltaic devices. However, this point itself is not new. Similar meta-analyses or cross-literature analyses have already been reported in recent years. Therefore, I suggest that the authors revise the related description and clarify what is really new in the present work.

2. The manuscript states “Recently, comparative cross-literature analyses have advanced the evaluation of scale-up beyond individual device optimization…”
However, no references are provided for this statement on cross-literature analyses. Since this is an important part of the background and motivation of the manuscript, relevant references should be added here.

3. An important equation, Eq. S18, defines: ΔFF_rec = FF_SQ – FF_0. The symbols used may be somewhat misleading. FF_SQ is not a quantity calculated completely independently from the expression used for FF_0. In fact, both FF_SQ and

FF_0 can be calculated from the same type of ideal-diode FF expression, namely the expression for FF_0 given in Eq. S15. The main difference is the input parameters used in the calculation. I suggest that the authors explain this point more clearly, or modify the formula symbols accordingly.

4. I suggest using n_id instead of simply n to denote the ideality factor. In line 272, the manuscript already uses “(n=15) per group” to indicate sample number. In semiconductor physics, n is also commonly used to denote electron density. To avoid ambiguity, it would be clearer to use n_id for the ideality factor throughout the manuscript.

5. From the results shown by the authors, the difference between FF_SQ and FF_0 is rather large. Previous studies (like ref.29) have compared FF_SQ and FF_0 assuming n_id=1, and in many cases the difference is usually small. Therefore, I suspect that the large difference observed here mainly comes from the deviation of the actual ideality factor n_id from unity, rather than mainly from changes in V_oc or J_0.

6. This raises an important question: should the change in ideal FF caused by n_id ≠ 1 be classified as “non-radiative recombination loss”? At present, the authors appear to mix the loss associated with V_oc and the loss associated with n_id together, and collectively refer to them as recombination loss. I suggest that the authors separate the loss caused by changes in V_oc from the loss caused by changes in n_id. In general, the loss reflected by V_oc can be regarded as recombination-related. However, in perovskite devices, the origin of changes in n_id can be complicated, involving shallow defects, interface effects, transport limitations, and other factors. Therefore, directly assigning this contribution to non-radiative recombination loss may not be fully accurate, and may not address the real physical origin of the FF change.

7. In line 208, the authors write” The decrease in J_0 by up to five orders of magnitude indicates substantially suppressed non-radiative recombination”
This interpretation should be made more cautiously. According to the open-circuit voltage expression derived from the illuminated single-diode equation, if the other parameters do not change significantly, a five-order-of-magnitude reduction in J_0 should lead to a clear increase in V_oc. Therefore, the authors need to explain why J_0 extracted from dark J–V fitting can decrease by five orders of magnitude, while the measured V_oc under illumination does not show a corresponding large increase.
In my view, this may indicate uncertainty in the extraction of J_0. The extraction of J_0 is usually quite sensitive and can be affected by other factors.

8. In line 193, the manuscript states: the increase in V_oc is small, whereas the improvement in FF is very clear. Since V_oc is usually considered to be strongly related to recombination, this creates a possible inconsistency. It again challenges the interpretation that strong suppression of non-radiative recombination is the main reason for the FF improvement. The authors should provide more data on n_id, discuss it in more detail, and show the influence of n_id on FF. Explaining the FF improvement from this angle may be more direct and convincing than simply attributing it to suppressed non-radiative recombination.

9. The PCE shown on the right y-axis of Fig. 1b is difficult to understand. In particular, the PCE of the authors’ samples in Fig. 1b appears to be below 20%, whereas Fig. 1a shows device PCE values above 24%. Do these two PCE values have the same meaning? If not, the difference should be clearly explained in the figure caption or in the main text.

10. I suggest that the authors mark their own device data at other active areas in Fig. 1c. This would make it easier to see how the present process affects the FF trend with increasing device area, and would also help readers compare the authors’ data with the scale-up trend in the literature.

11. I did not find a clear data availability statement in the manuscript. Since the article involves a large amount of data, providing the raw data would normally be important for improving the transparency and reproducibility of the results.

12. The manuscript includes a description of the TCSPC system, but no TRPL characterization seems to be presented. If TCSPC/TRPL measurements were not used in the analysis, this part should either be removed or clearly justified.

Reviewer #3

(Remarks to the Author)
This manuscript presents a fill-factor-centred framework for understanding efficiency losses during the scale-up of perovskite photovoltaics. Through a cross-literature analysis and systematic device characterization, the authors identify fill factor as a major contributor to cell-to-module efficiency loss. They further introduce a cryo-controlled crystallization strategy to prolong intermediate-phase evolution during film formation, leading to improved film quality, suppressed recombination, improved charge transport, and better FF preservation in scaled devices. The optimized 19.48 cm^2 mini-module achieves a certified steady-state efficiency of 23.9% with an FF of 83.3%, which represents an impressive result for module-level perovskite photovoltaics. Overall, the manuscript addresses an important issue in the field of scalable perovskite solar modules. The experimental results are strong, the device performance is impressive, and the analysis provides a useful perspective for connecting scale-up efficiency loss with FF degradation and operational stability. I believe the work is suitable for publication after minor revision.

1. The Methods state that “the wet perovskite films were cooled to 0–10 °C and subjected to vacuum flash for 2 min.” The

range of 0–10 °C is relatively broad for a temperature-sensitive crystallization process. Is device performance insensitive to temperature within this range? If so, please provide supporting data, for example film or device results obtained at several temperatures within this window. In addition, please specify the cooling duration and actual film temperature before vacuum flashing. Further details of the vacuum-flash process are also required, including the chamber volume, initial and final pressures, pump-down time, pressure–time profile, holding time at the target pressure, and the interval between cooling and vacuum treatment.
2. Supplementary Fig. 4 lacks a scale bar and does not specify the mapped area. Consequently, it is unclear whether the observed PL uniformity represents only a microscopic region or the entire coated film. Please provide both microscopic, micrometre-scale PL mapping and macroscopic, centimetre-scale mapping across the full substrate.
3. The KPFM profiles in Supplementary Fig. 5 appear to show appreciable local surface-potential heterogeneity, with a variation approaching 50 mV over a lateral distance of approximately 7 μm. Could the authors explain the origin of this variation and clarify whether it is reproducible across different locations and independently prepared films?
4. The compositional uniformity of the films is not sufficiently characterised. In particular, could the authors examine whether the Cs distribution differs between the control and target films or across different regions of the large-area coating? Spatial mapping of the PL peak position and/or elemental mapping by EDX would help determine whether the cryo-controlled process improves compositional homogeneity. Measurements at multiple positions across the substrate are recommended.
5. Please provide cross-sectional SEM images of the control and target devices. These images would allow comparison of the through-thickness grain structure, grain boundaries, void formation, layer thicknesses and interfacial contact quality. Such information may also help explain the observed improvement in FF after cryo-controlled processing.
6. In Supplementary Fig. 9, the target device exhibits higher Voc and FF but a slightly lower Jsc than the control device. However, the EQE spectra do not indicate an obvious change in the absorption onset or band edge (I notice that EQE is from minimodule). Could the authors explain the origin of the reduced Jsc?

Version 1:

Reviewer comments:

Reviewer #1

(Remarks to the Author)
The authors have made a genuine effort to address my concerns, particularly by reframing the innovation narrative from cryo-controlled processing to FF preservation as a strategy for mitigating efficiency loss and by providing a quantitative analysis of FF losses. However, FF preservation for mitigating efficiency loss is an opinion rather than a novelty or a genuine breakthrough in mechanism or methodology. I suggest the authors to rigorously differentiate their approach from prior art not just in terminology, but in demonstrable mechanistic distinctions.

Reviewer #2

(Remarks to the Author)
Thank you for the authors' careful and thorough responses. My previous comments have been well addressed, and I believe the manuscript has been substantially improved. In particular, the revised manuscript establishes a clearer and more convincing link between the processing strategy, the ideality factor, and FF preservation. Such a quantitative connection remains relatively underexplored, particularly in high performance perovskite photovoltaic devices. I therefore recommend acceptance of the manuscript in its present form.

Reviewer #3

(Remarks to the Author)
The authors have adequately addressed the concerns raised in my previous review. The revisions and additional clarifications have satisfactorily resolved the major issues, and the manuscript has been further improved. I therefore recommend the manuscript for publication in Nature Communications.

Open Access This Peer Review File is licensed under a Creative Commons Attribution 4.0 International License, which permits use, sharing, adaptation, distribution and reproduction in any medium or format, as long as you give appropriate credit to the original author(s) and the source, provide a link to the Creative Commons license, and indicate if changes were made.
In cases where reviewers are anonymous, credit should be given to 'Anonymous Referee' and the source.
The images or other third party material in this Peer Review File are included in the article's Creative Commons license, unless indicated otherwise in a credit line to the material. If material is not included in the article's Creative Commons license and your intended use is not permitted by statutory regulation or exceeds the permitted use, you will need to obtain permission directly from the copyright holder.
To view a copy of this license, visit https://creativecommons.org/licenses/by/4.0/

Response to Reviewers' Comments

We thank the Editor for organizing the review and the reviewers for their careful and constructive evaluations. The reviewers positively evaluated the strong device performance and the value of our FF-centred analysis for understanding scale-up efficiency loss and operational stability, while providing many constructive suggestions to further strengthen the work. We have taken these comments fully on board and carefully addressed them throughout the revised manuscript and Supplementary Information. All changes are highlighted in yellow for ease of reference.

REVIEWER COMMENTS:

Reviewer #1:

This manuscript proposes a strategy to preserve the fill factor (FF) of perovskite solar modules by controlling the crystallization at low temperature. By prolonging the evolution of the intermediate phase, improving the crystallization quality of the perovskite films, and suppressing recombination losses, the resulting minimodule with an aperture area of 19.48 cm² achieves an impressing result with a certified efficiency of 23.9%. However, this work fails to demonstrate substantial innovation beyond existing literature. Similar approaches have already been reported in previous studies (Nat. Commun. 15, 6679, 2024; Energy Environ. Sci. 18, 10434-10443, 2025), in which low temperature was used to regulate intermediate-phase evolution, solvent evaporation, and perovskite crystallization. Besides, the central opinion using FF as a key descriptor for the efficiency loss and operation stability is not convincing, as FF is strongly connected with other parameters. Therefore, I cannot suggest its publication on Nature Communications.

Response: We thank the reviewer for evaluating our work and for the positive assessment of the mini-module performance. We have also carefully considered the comments regarding the innovation of the work and the use of FF as a key descriptor of scale-up loss and operational stability, and have now substantially revised the manuscript to clarify the central advance and strengthen the basis for the FF analysis.

The central advance of the present work is to establish FF preservation as a strategy for mitigating scale-up-induced efficiency loss. This is supported by direct quantitative analysis of practical cell-to-module efficiency loss, which reveals FF as a major contribution across device scales; by resolving the factors governing FF loss and preservation; and by cross-scale experimental validation showing that FF preservation increasingly offsets scale-up-induced loss, alongside a certified 23.9% mini-module. The cryo-controlled processing is not presented as an innovation point; rather, it provides an experimental implementation for testing and demonstrating this FF-preservation strategy. We have now made this role explicit in the Introduction, page 3, and added the references mentioned by the reviewer:

"Here, we establish FF preservation as a strategy for mitigating scale-up-induced efficiency loss, grounded in a direct quantitative attribution of the measured cell-to-module efficiency penalty to V_{oc}, J_{sc} and FF, together with a parameter-level

analysis of FF loss and preservation. To experimentally demonstrate this FF-preservation strategy, we employ cryo-controlled processing to regulate crystallization and preserve FF during scale-up[5,12,27]."

Our focus on FF arises from the power-conversion-efficiency (PCE) loss analysis rather than an a priori choice. PCE is directly determined by V_{oc}, J_{sc} and FF (PCE=V_{oc} J_{sc} FF/P_{in}); we therefore evaluate the contributions of all three parameters to the measured CTM efficiency loss on the same basis. This analysis directly illustrates that FF is the largest contributor across diverse device technologies and fabrication routes, providing the basis for subsequently focusing on FF preservation.

The strong connection of FF with other photovoltaic parameters does not undermine its use as a performance descriptor; rather, it motivates further analysis of the factors underlying its loss and preservation. We therefore substantially extended the analysis to distinguish the V_{oc}-related, n_{id}-associated and transport-associated contributions to FF. This analysis first resolves the major FF deficit identified during scale-up (revised Fig. 1d), and then shows how the FF advantage of the target devices is achieved and retained across device scales and during ageing (revised Figs. 4c,e and 5c,f). The corresponding analysis and discussion have been revised throughout the manuscript, with the decomposition method detailed in Supplementary Note 2.

In the "Efficiency loss during scale-up" section, page 4, second paragraph, the revised text now reads:

"To quantify the parameter-level basis of this FF retention, we stepwise analysed the deficit between the measured FF and its theoretical upper limit, determined by Shockley–Queisser analysis, into V_{oc}-related, n_{id}-associated and transport-associated components…"

In the "Fill-factor preservation" section, pages 9–10, the revised text now reads:

"Analysis of the FF deficit relative to its theoretical upper bound revealed that the V_{oc}-related loss remained nearly unchanged, whereas the reduction in FF loss arose predominantly from the n_{id}-associated term, with a smaller contribution from the transport-associated term…"

"Quantification of the individual parameter contributions further showed that the V_{oc}-, n_{id}-, R_s-, R_{sh}-associated terms contributed 0.22, 3.72, 0.40 and 0.20 percentage points, respectively, to the FF improvement."

In the "Scalability and stability" section, pages 11–12, the revised text now reads:

"Stepwise attribution of the FF gain showed that the n_{id}-associated contribution became increasingly dominant throughout scaling, whereas the V_{oc}-related and transport-associated contributions remained small…"

"Further partitioning of FF degradation showed that the improved FF stability manifested predominantly as a smaller increase in the n_{id}-associated contribution, while transport-associated loss was also reduced…"

These revisions directly address the reviewer's concern by showing that FF is identified from the practical efficiency-loss analysis as the principal performance bottleneck, while its dependence on other photovoltaic parameters is explicitly resolved to understand the origin and preservation of FF during scale-up and operation.

Reviewer #2:

This manuscript focuses on FF preservation during the scale-up of perovskite photovoltaic devices from small-area cells to mini-modules. The authors propose a cryo-controlled processing strategy to regulate the evolution of the intermediate phase during crystallization, thereby improving film formation and reducing FF losses. Overall, this processing strategy appears to be effective in improving FF, device reproducibility, and stability. The mini-module efficiency and FF are also quite good, which is a clear strength of the work. Another interesting point is that this process seems to mainly improve FF, while having only a limited effect on the other photovoltaic parameters. Such selective FF enhancement is relatively uncommon and provides a useful entry point for studying the origin of FF losses.

However, I still have several concerns about the mechanistic interpretation. In particular, the authors argue that the FF loss during scale-up mainly originates from recombination-related losses, and further attribute the FF improvement from cryo-controlled processing mainly to recombination suppression. At present, the discussion of the ideality factor n_id is not sufficiently clear, and some interpretations appear to be somewhat inconsistent. I think these issues should be clarified before publication and would require substantial revision.

Response: We sincerely thank the reviewer for the highly positive assessment of our work, as well as for the careful examination and constructive suggestions. Following these helpful suggestions, we have substantially revised the interpretation of FF loss and preservation by separating the V_{oc}-related and n_{id}-associated contributions to avoid conflating diode non-ideality with recombination-related loss and to more accurately identify the origins of the observed FF changes. We have further analysed the role of n_{id} across device scales and revised the corresponding FF-decomposition analysis, figures and discussion throughout the manuscript.

1. In the introduction and background sections, the authors emphasize that one of the highlights of this work is to show that FF is the dominant loss during the scale-up of perovskite photovoltaic devices. However, this point itself is not new. Similar meta-analyses or cross-literature analyses have already been reported in recent years. Therefore, I suggest that the authors revise the related description and clarify what is really new in the present work.

Response: We thank the reviewer for highlighting the need to more precisely describe the novelty of the present work. Previous cross-literature analyses using figures of merit referenced to the Shockley-Queisser (SQ) limit compare how V_{oc}-, J_{sc}- and FF-associated losses evolve relative to the SQ limit across diverse device scales and fabrication routes. Our analysis instead directly attributes the measured CTM efficiency penalty itself to V_{oc}, J_{sc} and FF, providing a more direct and intuitive measure of the practical impact of each parameter on the actual efficiency lost during scale-up. Building on this quantitative attribution, we further perform a parameter-level analysis of FF loss and preservation and establish FF preservation as a strategy for mitigating scale-up-induced efficiency loss.

Accordingly, we have revised the Introduction, page 3, in three respects:

(1) We now *explicitly acknowledge the contribution* of previous cross-literature analyses and their conclusion regarding FF:

"More recently, figures of merit referenced to the Shockley-Queisser (SQ) limit[30] have been used in cross-literature analyses to compare the evolution of losses associated with open-circuit voltage (V_{oc}), short-circuit current density (J_{sc}) and fill factor (FF) during scale-up across diverse fabrication routes, suggesting that FF generally represents the largest loss during upscaling[31]. Such analyses provide a broader picture of how V_{oc}-, J_{sc}- and FF-associated losses evolve with increasing device scale."

(2) We *clarify the remaining gap and the distinction* of our analysis-direct attribution of the measured CTM efficiency loss-and connect this to the subsequent question of the origin and control of FF loss:

"Yet, a direct quantitative attribution of the measured CTM efficiency loss to V_{oc}, J_{sc} and FF remains lacking. More importantly, understanding the origin of the pronounced FF loss during scale-up is essential for developing effective strategies for preserving FF and thereby mitigating the associated CTM efficiency loss."

(3) We accordingly *redefine the advance* of the present work as extending from direct quantitative attribution to the analysis and preservation of FF, rather than the observation of FF dominance itself:

"Here, we establish FF preservation as a strategy for mitigating scale-up-induced efficiency loss, grounded in a direct quantitative attribution of the measured cell-to-module efficiency penalty to V_{oc}, J_{sc} and FF, together with a parameter-level analysis of FF loss and preservation."

2. The manuscript states "Recently, comparative cross-literature analyses have advanced the evaluation of scale-up beyond individual device optimization…"
However, no references are provided for this statement on cross-literature analyses. Since this is an important part of the background and motivation of the manuscript, relevant references should be added here.

Response: Thank you and we have now cited Refs. 30 and 31 here to support the statement on comparative cross-literature analyses.

3. An important equation, Eq. S18, defines: ΔFF_rec = FF_SQ − FF_0. The symbols used may be somewhat misleading. FF_SQ is not a quantity calculated completely independently from the expression used for FF_0. In fact, both FF_SQ and FF_0 can be calculated from the same type of ideal-diode FF expression, namely the expression for FF_0 given in Eq. S15. The main difference is the input parameters used in the calculation. I suggest that the authors explain this point more clearly, or modify the formula symbols accordingly.

Response: We thank the reviewer for suggesting this clarification. We have revised Supplementary Note 2 to clarify the relationship between FF_{SQ} and FF_0, as shown in the revised Eqs. S15-S21 and associated text. In particular, FF_{SQ} is now explicitly expressed as $FF_{SQ} = FF_0\left(V_{oc}^{SQ}, 1\right)$, showing that it is obtained from the same resistance-free FF expression by using the SQ-limit inputs. The original ΔFF_{rec} has

also been replaced by the V_{oc}-related contribution, $\Delta FF_{V_{oc}}$, in line with the suggestion in Comment No. 6. These revisions clarify the notation and the relationship between the corresponding FF quantities as suggested by the reviewer.

4. I suggest using n_id instead of simply n to denote the ideality factor. In line 272, the manuscript already uses "(n=15) per group" to indicate sample number. In semiconductor physics, n is also commonly used to denote electron density. To avoid ambiguity, it would be clearer to use n_id for the ideality factor throughout the manuscript.

Response: Thank you, and we have now replaced n with n_{id} for the ideality factor and N for sample number.

5. From the results shown by the authors, the difference between FF_SQ and FF_0 is rather large. Previous studies (like ref.29) have compared FF_SQ and FF_0 assuming n_id=1, and in many cases the difference is usually small. Therefore, I suspect that the large difference observed here mainly comes from the deviation of the actual ideality factor n_id from unity, rather than mainly from changes in V_oc or J_0.

Response: The reviewer is correct that the large difference mainly arises from the deviation of n_{id} from unity. In the reference mentioned by the reviewer (now Ref. 31), FF_0 is evaluated assuming $n_{id} = 1$, whereas in our revised analysis $FF_0(V_{oc}, n_{id})$ explicitly incorporates the measured V_{oc} and n_{id}, rather than assuming $n_{id} = 1$, as given in Eq. S16. Accordingly, the difference between FF_{SQ} and $FF_0(V_{oc}, n_{id})$ is now separated into the V_{oc}-related and n_{id}-associated contributions in Eqs. S18 and S19, respectively, in line with the suggestion provided in Comment No. 6. We have therefore revised the analysis to explicitly quantify the influence of n_{id} on FF. As shown in the revised Fig. 1d, the n_{id}-associated FF deficit is substantially larger than the V_{oc}-related deficit. The substantial influence of n_{id} on FF is further evident in the revised Fig. 4c,e and Fig. 5c,f. These results confirm the reviewer's interpretation that the large difference is primarily associated with the deviation of n_{id} from unity.

6. This raises an important question: should the change in ideal FF caused by n_id ≠ 1 be classified as "non-radiative recombination loss"? At present, the authors appear to mix the loss associated with V_oc and the loss associated with n_id together, and collectively refer to them as recombination loss. I suggest that the authors separate the loss caused by changes in V_oc from the loss caused by changes in n_id. In general, the loss reflected by V_oc can be regarded as recombination-related. However, in perovskite devices, the origin of changes in n_id can be complicated, involving shallow defects, interface effects, transport limitations, and other factors. Therefore, directly assigning this contribution to non-radiative recombination loss may not be fully accurate, and may not address the real physical origin of the FF change.

Response: We thank the reviewer for highlighting the need for a more careful physical interpretation of the n_{id}-associated FF loss. We agree that this contribution should not be directly assigned to non-radiative recombination, and have revised the manuscript accordingly.

First, we have removed the direct attribution of changes in n_{id} to non-radiative recombination and clarified the possible origins of n_{id}. In the "Recombination-loss suppression" section, page 7, the previous statement that the decrease in n_{id} was "consistent with suppressed non-radiative recombination" has been revised to:

"The increase in V_{oc} is consistent with reduced recombination at the device level, whereas the change in n_{id} reflects altered diode non-ideality whose physical origin is not uniquely attributable to non-radiative recombination."

In the "Efficiency loss during scale-up" section, page 4, we further clarify that:

"The V_{oc}-related component represents the FF loss associated with the recombination-related voltage deficit, whereas the n_{id}-associated component captures the FF loss due to diode non-ideality, potentially reflecting defect, interfacial, ionic and other effects[36-38]."

Second, following the reviewer's suggestion, we have now separated the V_{oc}-related and n_{id}-associated contributions in the FF analysis. Supplementary Note 2 has been revised to quantify these contributions separately as $\Delta FF_{V_{oc}}$ and $\Delta FF_{n_{id}}$ in Eqs. S18 and S19, respectively. The corresponding analyses in the main text and figures have been recalculated and revised accordingly: Fig. 1d now shows the separation of the FF deficit into V_{oc}-related, n_{id}-associated and transport-associated contributions; Fig. 4c,e applies this analysis to FF preservation in the control and target mini-modules; and Fig. 5c,f extends the analysis across device scales and during ageing. The revised analysis shows that the n_{id}-associated contribution to FF preservation is predominant, whereas the V_{oc}-related and transport-associated contributions are comparatively small.

Based on this revised analysis, we have also updated the corresponding interpretation throughout the manuscript. The Abstract, page 2, now states:

"Further analysis shows that the FF-preservation advantage arises predominantly from reduced diode non-ideality, with smaller contributions associated with the recombination-related voltage deficit and transport-related losses."

The Introduction, page 3, now states:

"Stepwise analysis reveals that the resulting FF preservation manifests predominantly

through the contribution associated with the diode ideality factor (n_{id}), with smaller contributions associated with the recombination-related voltage deficit and transport."

The Conclusion, page 14, has correspondingly been revised to state:

"Stepwise analysis further shows that the FF-preservation advantage arises predominantly from reduced n_{id}-associated loss, with the other contributions being comparatively smaller."

Together, these revisions separate the V_{oc}-related and n_{id}-associated FF contributions and avoid assigning the latter uniquely to non-radiative recombination, in line with the reviewer's suggestion.

7. In line 208, the authors write" The decrease in J_0 by up to five orders of magnitude indicates substantially suppressed non-radiative recombination"
This interpretation should be made more cautiously. According to the open-circuit voltage expression derived from the illuminated single-diode equation, if the other parameters do not change significantly, a five-order-of-magnitude reduction in J_0 should lead to a clear increase in V_oc. Therefore, the authors need to explain why J_0 extracted from dark J–V fitting can decrease by five orders of magnitude, while the measured V_oc under illumination does not show a corresponding large increase.
In my view, this may indicate uncertainty in the extraction of J_0. The extraction of J_0 is usually quite sensitive and can be affected by other factors.

Response: The reviewer is correct that the large variation in J_0 is due to the high sensitivity and uncertainty of its extraction and should not be interpreted as an equivalent change in the physical recombination current. The seemingly disproportionate changes in J_0 and measured V_{oc} do not imply a physical inconsistency, because J_0 is not independently measured but is jointly constrained with n_{id} and the resistance-related parameters in the single-diode fitting. Under the open-circuit condition, once V_{oc}, n_{id}, J_{ph} and R_{sh} are specified, J_0 is correspondingly constrained by the diode equation. Therefore, a large change in the extracted J_0 does not by itself imply a correspondingly large change in V_{oc}.

To avoid this potential confusion, and following the revised FF analysis introduced in response to Comment No. 6, we have removed the previous J_0-based interpretation. This does not remove information from the FF analysis because the resistance-free $FF_0(V_{oc}, n_{id})$ is now determined directly from the measured V_{oc} and n_{id}, while the resistive correction is determined separately from R_s and R_{sh}. Thus, all quantities required for the revised FF analysis are explicitly retained, and J_0 does not constitute an additional independent term within this framework.

We have consequently removed J_0 from the corresponding FF-preservation

analysis rather than retaining a parameter whose extracted magnitude cannot be assigned an independent quantitative physical interpretation. The previous statement that the decrease in J_0 by up to five orders of magnitude indicated substantially suppressed non-radiative recombination, together with the associated J_0-based interpretation in the Introduction, has been removed. The revised FF-preservation analysis is instead based on the directly evaluated V_{oc}-related, n_{id}-associated and transport-associated contributions (Supplementary Note 2).

8. In line 193, the manuscript states: the increase in V_oc is small, whereas the improvement in FF is very clear. Since V_oc is usually considered to be strongly related to recombination, this creates a possible inconsistency. It again challenges the interpretation that strong suppression of non-radiative recombination is the main reason for the FF improvement. The authors should provide more data on n_id, discuss it in more detail, and show the influence of n_id on FF. Explaining the FF improvement from this angle may be more direct and convincing than simply attributing it to suppressed non-radiative recombination.

Response: We thank the reviewer for this further suggestion. The possible inconsistency noted by the reviewer has now been resolved through the revised FF analysis introduced in response to Comment No. 6. We now separate the V_{oc}-related and n_{id}-associated contributions and no longer attribute the main FF improvement to suppressed non-radiative recombination. Instead, the revised analysis shows that the FF improvement is predominantly associated with the n_{id}-associated contribution, which is not uniquely attributable to non-radiative recombination.

To incorporate the reviewer's suggestion to provide additional n_{id} data, discuss it in more detail and show its influence on FF, we have added a dedicated cross-scale analysis in Supplementary Note 3. This analysis compares the evolution of n_{id} and V_{oc} from cells to mini-modules and modules and evaluates their respective influences on FF through the resistance-free $FF_0(V_{oc}, n_{id})$. In particular, we added a figure entitled "Dependence of the resistance-free fill factor on n_{id} at fixed V_{oc} values" in Supplementary Note 3 to directly illustrate the influence of n_{id} on FF. The results show that, with increasing device scale, the n_{id} advantage becomes progressively larger while the V_{oc} advantage diminishes, supporting that the increasing FF advantage is predominantly associated with the n_{id}-associated contribution.

Accordingly, we have revised the corresponding interpretation in the main text. In the "Scalability and stability" section, page 11, the revised text now reads:

"Stepwise attribution of the FF gain showed that the n_{id}-associated contribution became increasingly dominant throughout scaling, whereas the V_{oc}-related and transport-associated contributions remained small (Fig. 5c), consistent with the scale-dependent V_{oc} and n_{id} analysis in Supplementary Note 3."

These revisions directly address the reviewer's suggestion by providing additional n_{id} data, quantifying its influence on FF, and revising the interpretation of the FF improvement accordingly.

9. The PCE shown on the right y-axis of Fig. 1b is difficult to understand. In particular, the PCE of the authors' samples in Fig. 1b appears to be below 20%, whereas Fig. 1a shows device PCE values above 24%. Do these two PCE values have the same meaning? If not, the difference should be clearly explained in the figure caption or in the main text.

Response: We thank the reviewer for pointing out the ambiguity in the original presentation of Fig. 1b. The stacked bars represent the contributions to CTM efficiency loss and correspond to the left y-axis, whereas the circles and star represent the corresponding module PCEs and correspond to the right y-axis. Thus, the apparent discrepancy arises from the insufficiently explicit distinction between these two graphical elements in the original figure. We have revised the figure legend and caption to explicitly distinguish the stacked bars from the circles and star and their respective y-axes.

10. I suggest that the authors mark their own device data at other active areas in Fig. 1c. This would make it easier to see how the present process affects the FF trend with increasing device area, and would also help readers compare the authors' data with the scale-up trend in the literature.

Response: We thank the reviewer for this further suggestion regarding the comparison of our FF results across device scales. Fig. 1c is intended to show the general cross-literature trend of FF with device area, with a representative high-performance device selected from each study rather than the area-dependent trajectory within individual studies. We therefore retain the representative device from this work in Fig. 1c. The effect of the present processing strategy on FF across device scales is evaluated more directly through matched control-target comparisons in Fig. 5c and Supplementary Note 3, covering cells, mini-modules and modules.

These analyses show that the FF gain from control to target increases with device area, as shown by the increasing orange contribution in Fig. 5c, thereby increasingly offsetting scale-up-induced FF loss and improving FF preservation in the target devices. We have therefore retained Fig. 1c as the cross-literature comparison, while Fig. 5c and Supplementary Note 3 provide the corresponding comparison of FF preservation across device scales.

11. I did not find a clear data availability statement in the manuscript. Since the article involves a large amount of data, providing the raw data would normally be important for improving the transparency and reproducibility of the results.

Response: We thank the reviewer for bringing this to our attention. In line with the reviewer's comment and the editorial requirement, we have added a Data Availability statement and provided the raw data underlying the figures in the main text and Supplementary Information.

12. The manuscript includes a description of the TCSPC system, but no TRPL characterization seems to be presented. If TCSPC/TRPL measurements were not used in the analysis, this part should either be removed or clearly justified.

Response: We thank the reviewer for noting this. The TCSPC/TRPL measurements were not used in the present study, and we have therefore removed the corresponding instrument description from the Methods.

Reviewer #3:

This manuscript presents a fill-factor-centred framework for understanding efficiency losses during the scale-up of perovskite photovoltaics. Through a cross-literature analysis and systematic device characterization, the authors identify fill factor as a major contributor to cell-to-module efficiency loss. They further introduce a cryo-controlled crystallization strategy to prolong intermediate-phase evolution during film formation, leading to improved film quality, suppressed recombination, improved charge transport, and better FF preservation in scaled devices. The optimized 19.48 cm² mini-module achieves a certified steady-state efficiency of 23.9% with an FF of 83.3%, which represents an impressive result for module-level perovskite photovoltaics. Overall, the manuscript addresses an important issue in the field of scalable perovskite solar modules. The experimental results are strong, the device performance is impressive, and the analysis provides a useful perspective for connecting scale-up efficiency loss with FF degradation and operational stability. I believe the work is suitable for publication after minor revision.

Response: We thank the reviewer for the highly positive assessment and support of our work. We have now carefully addressed all comments in the revised manuscript.

1. The Methods state that "the wet perovskite films were cooled to 0–10 °C and subjected to vacuum flash for 2 min." The range of 0–10 °C is relatively broad for a temperature-sensitive crystallization process. Is device performance insensitive to temperature within this range? If so, please provide supporting data, for example film or device results obtained at several temperatures within this window. In addition, please specify the cooling duration and actual film temperature before vacuum flashing. Further details of the vacuum-flash process are also required, including the chamber volume, initial and final pressures, pump-down time, pressure–time profile, holding time at the target pressure, and the interval between cooling and vacuum treatment.

Response: We thank the reviewer for the helpful suggestions on improving the experimental details. To clarify the influence of the film temperature before vacuum flashing, we performed additional measurements on 19.48 cm^2 mini-modules processed at film temperatures of 0, 5 and 10 °C immediately before vacuum flashing. As shown in the new Supplementary Fig. 17, the mini-modules exhibit nearly overlapping I-V characteristics, with PCEs of 23.68%, 23.65% and 23.74% at 0, 5 and 10 °C, respectively, while V_{oc}, J_{sc} and FF also remain nearly unchanged. These results show that device performance is insensitive to film temperature within the investigated 0–10 °C range.

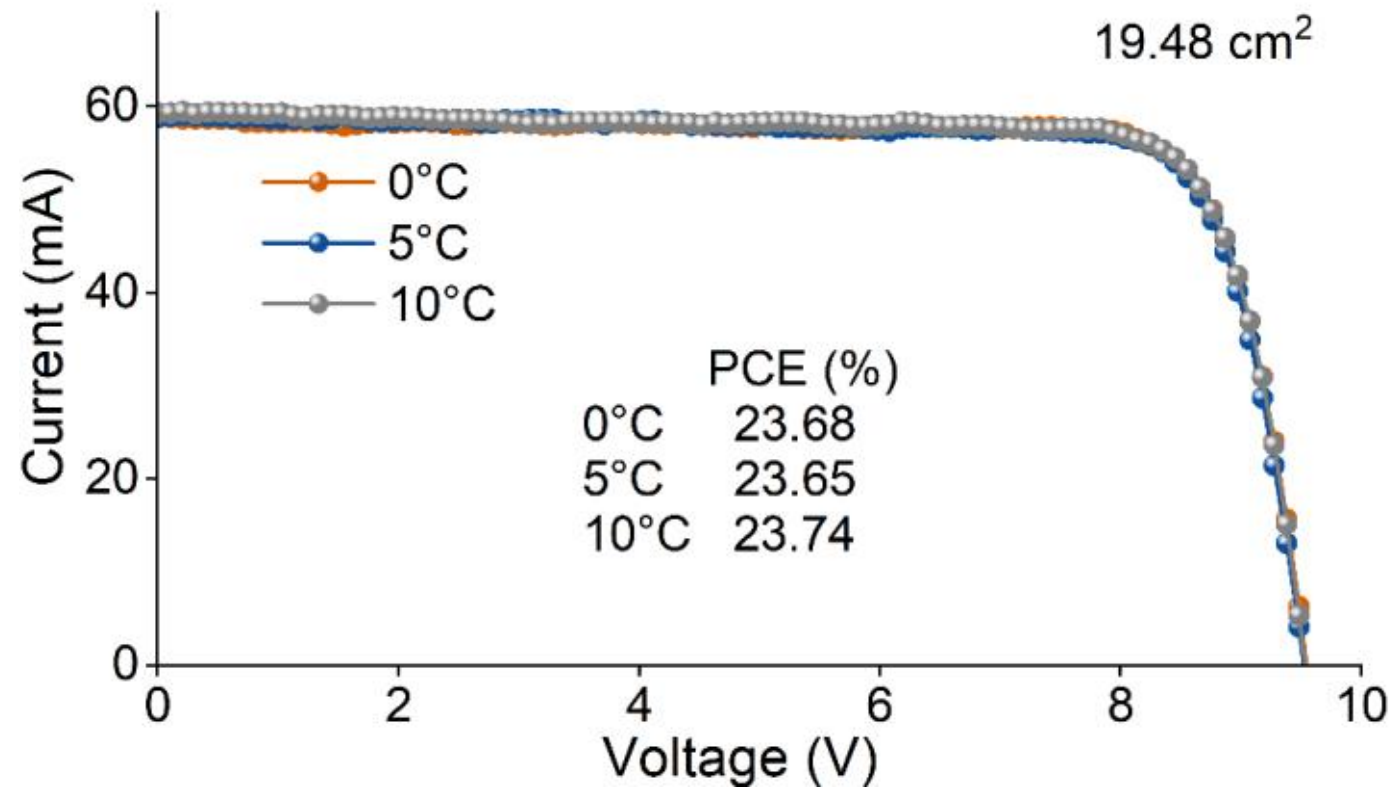

Supplementary Fig. 17 | Effect of film temperature before vacuum flashing on device performance. Current–voltage (*I*–*V*) characteristics of 19.48 cm^2 perovskite mini-modules processed at film temperatures of 0, 5 and 10 °C immediately before vacuum flashing. The corresponding PCEs are 23.68%, 23.65% and 23.74%, respectively.

In line with the reviewer's suggestion, we have also added further details of the cooling and vacuum-flash procedures to the revised Methods. In the "Device fabrication—Solar-cell fabrication" section, page 16, we have now added the following description:

"Immediately after spin-coating, the wet perovskite films were cooled for 15 s, reaching an experimentally measured film temperature of 0-10 °C immediately before vacuum treatment (Supplementary Fig. 17). The films were then transferred to the vacuum-flash chamber within approximately 5 s. Vacuum flashing was performed in a chamber with an effective internal volume of approximately 69.6 cm^3 for a total treatment time of 120 s. The chamber pressure was initially approximately 1.0×10^5 Pa and decreased to below 10 Pa within approximately 8 s. During the remaining approximately 112 s, the pressure gradually decreased from approximately 10 Pa to approximately 5 Pa, reaching a final pressure of approximately 5 Pa (Supplementary Fig. 18)."

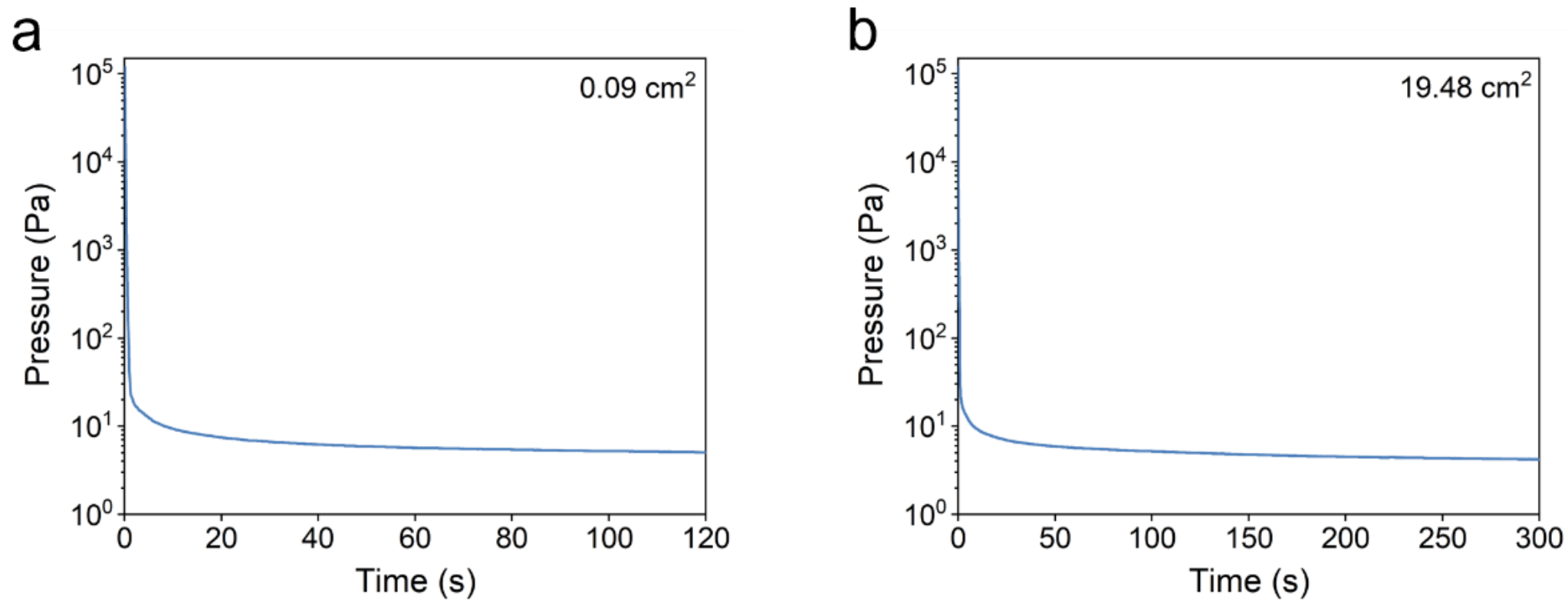

Supplementary Fig. 18 | Pressure–time profiles during the vacuum-flash process. **a**, Pressure evolution during vacuum flashing for the 0.09 cm^2 device, with a total treatment duration of 120 s. **b**, Pressure evolution during vacuum flashing for the 19.48 cm^2 mini-module, with a total treatment duration of 300 s.

2. Supplementary Fig. 4 lacks a scale bar and does not specify the mapped area. Consequently, it is unclear whether the observed PL uniformity represents only a microscopic region or the entire coated film. Please provide both microscopic, micrometre-scale PL mapping and macroscopic, centimetre-scale mapping across the full substrate.

Response: We thank the reviewer for the helpful suggestion on clarifying the characterization scale. The original PL mapping (original Supplementary Fig. 4, now Supplementary Fig. 5) was acquired at the micrometre scale, and we have now added a scale bar to clearly indicate the mapped area, as shown below.

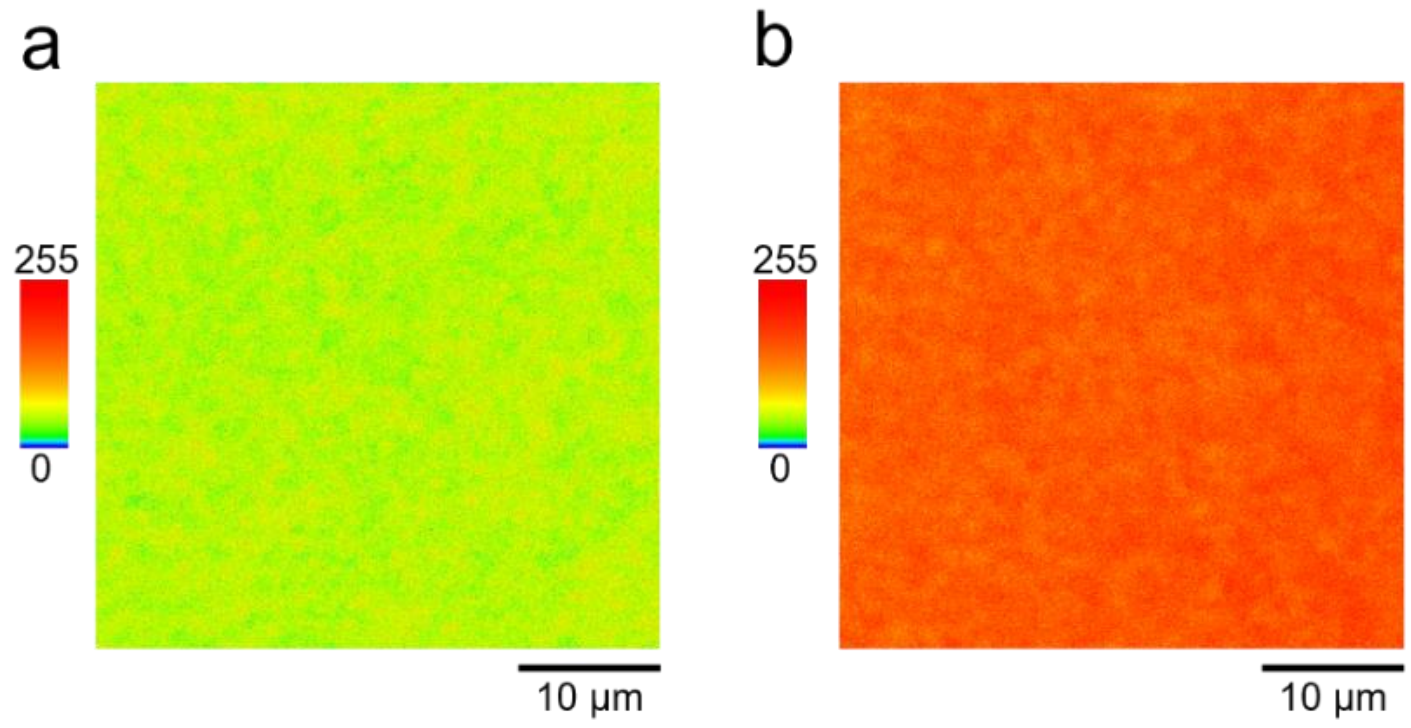

Supplementary Fig. 5 | Spatial photoluminescence (PL) mapping of perovskite films. a, Control perovskite films. **b,** Target perovskite films.

As centimetre-scale PL mapping was not available with our measurement setup, we further assessed the macroscopic spatial uniformity by full-area EL imaging of the completed mini-modules (Supplementary Fig. 7). This provides device-level characterization over a substantially larger spatial scale and directly evaluates the emission uniformity across the full mini-module area. The target mini-module exhibits a more uniform EL distribution than the control device, indicating improved spatial uniformity at the device scale. The newly added Supplementary Fig. 7 is shown below.

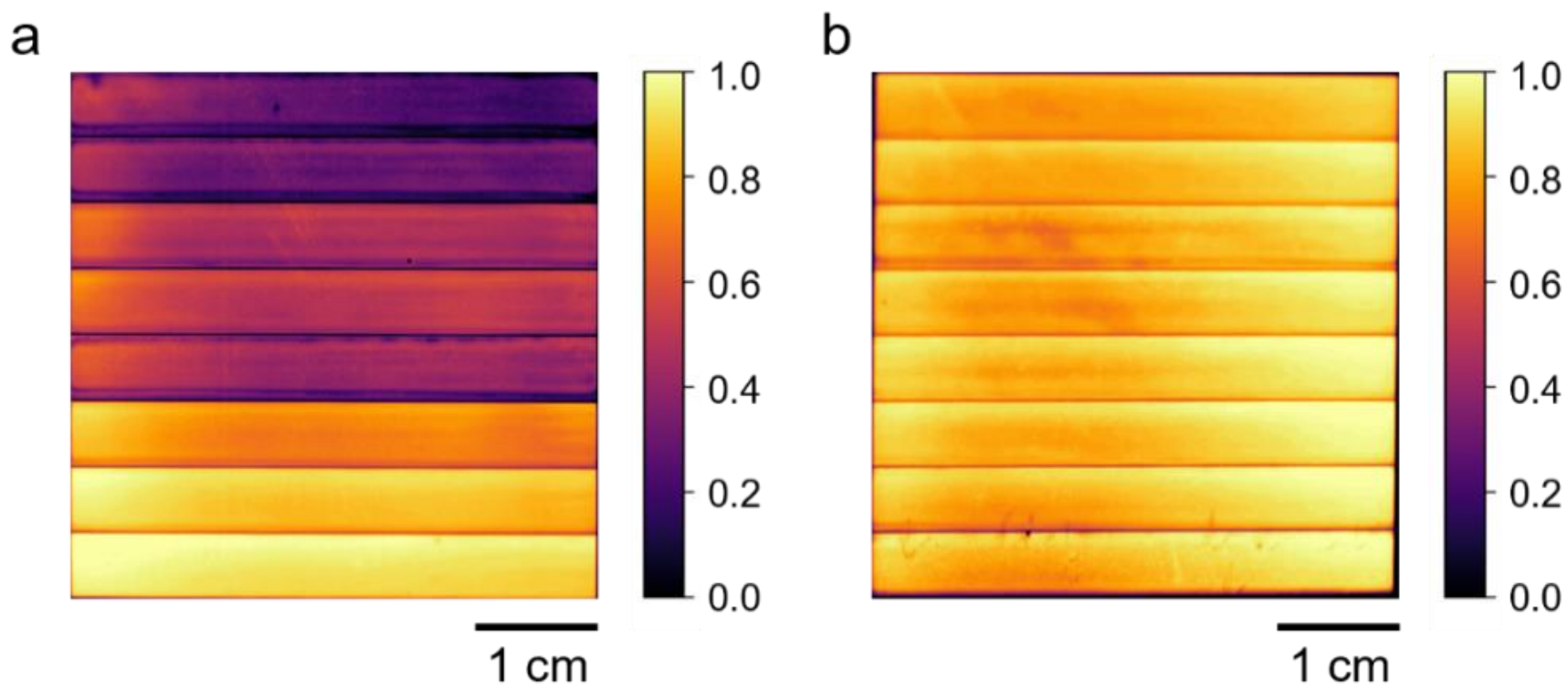

Supplementary Fig. 7 | Spatial distribution of normalized electroluminescence (EL) intensity in perovskite minimodules. a, Control minimodule. **b,** Target minimodule.

Accordingly, in the “Recombination-loss suppression” section, page 7, second paragraph, the revised text now reads:

"At the device level, EL imaging further revealed a more spatially uniform emission distribution across the target mini-module (Supplementary Fig. 7)."

3. The KPFM profiles in Supplementary Fig. 5 appear to show appreciable local surface-potential heterogeneity, with a variation approaching 50 mV over a lateral distance of approximately 7 μm. Could the authors explain the origin of this variation and clarify whether it is reproducible across different locations and independently prepared films?

Response: We thank the reviewer for raising this question. To evaluate the reproducibility of the observed surface-potential variation, we performed additional KPFM measurements on independently prepared control and target films at multiple spatially separated regions. The original Supplementary Fig. 5 has accordingly been updated with these additional measurements and is now Supplementary Fig. 8. The pronounced monotonic variation observed in the original single line scan was not universally observed at different locations, indicating that it represents a local surface-potential variation rather than a persistent film-wide potential gradient. Importantly, the relative surface-potential shift between the control and target films was consistently reproduced across different measured regions, with average differences of approximately 39 and 37 mV obtained from the original and additional KPFM measurements, respectively.

Accordingly, Fig. 3c, d have been updated. In the "Charge transport improvement" section, page 8, first paragraph, the revised text now reads:

"The target film showed a higher surface potential than the control film (Fig. 3c, d and Supplementary Fig. 8), corresponding to a relative reduction in surface work function."

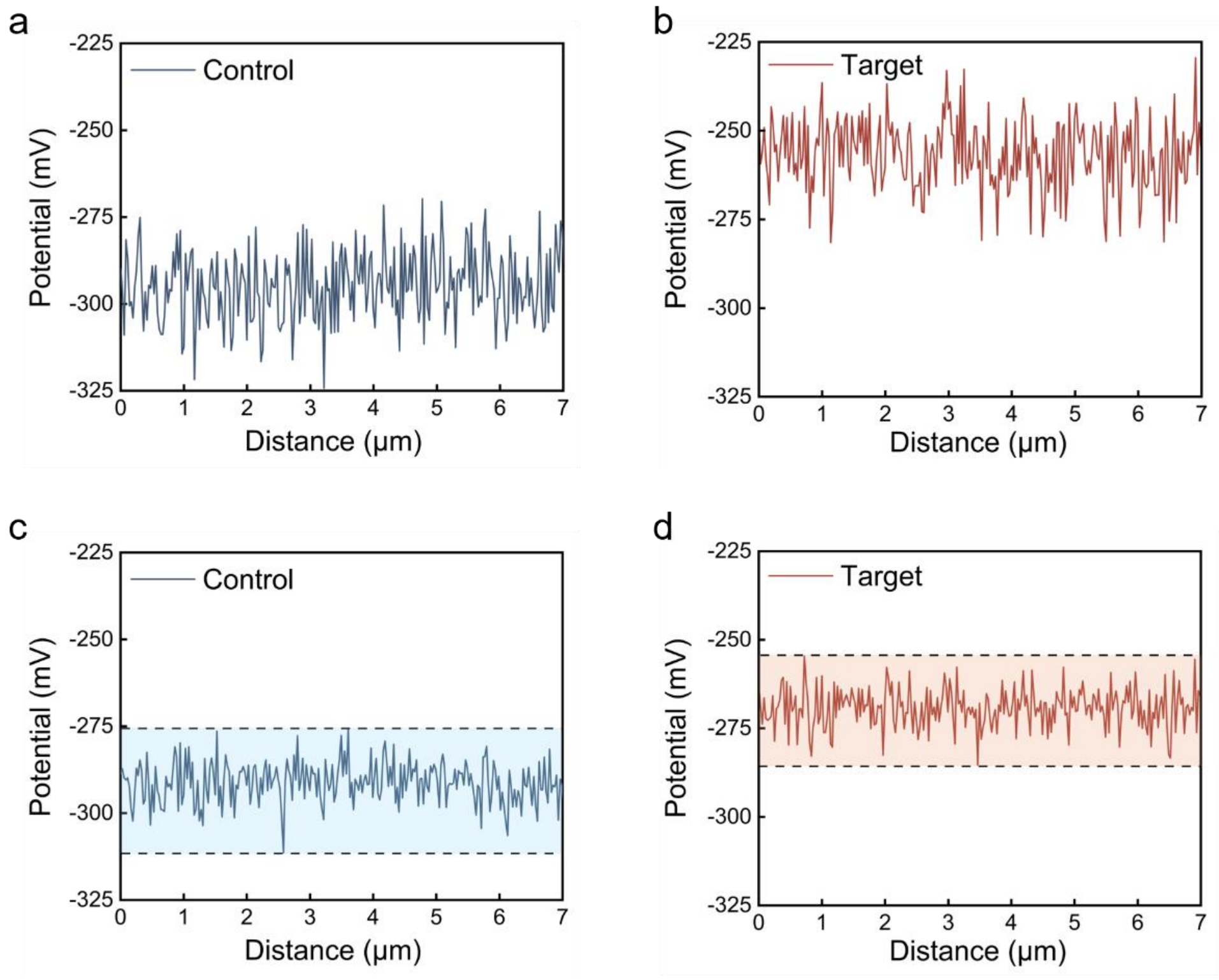

Supplementary Fig. 8 | Kelvin probe force microscopy (KPFM) surface-potential profiles of the control and target perovskite films. a, b, Surface-potential line profiles extracted along the diagonal directions of the representative control (**a**) and target (**b**) KPFM maps shown in Fig. 3. **c, d,** Mean surface-potential profiles obtained by averaging line profiles measured at three spatially separated regions of the control (**c**) and target (**d**) films, respectively.

4. The compositional uniformity of the films is not sufficiently characterised. In particular, could the authors examine whether the Cs distribution differs between the control and target films or across different regions of the large-area coating? Spatial mapping of the PL peak position and/or elemental mapping by EDX would help determine whether the cryo-controlled process improves compositional homogeneity. Measurements at multiple positions across the substrate are recommended.

Response: We thank the reviewer for this helpful advice. We have now performed energy-dispersive X-ray spectroscopy (EDX/EDS) measurements at nine spatially distributed positions across both the control and target perovskite films (Supplementary Fig. 6). The Cs/Pb atomic ratios were 0.1040 ± 0.0075 and 0.0990 ± 0.0056 for the control and target films, respectively (mean ± s.d., $N = 9$ spatial positions). Both films show good compositional uniformity across the sampled regions, with the target film exhibiting a smaller position-to-position variation in the Cs/Pb atomic ratio. In the "Recombination-loss suppression" section, page 6, second paragraph, we have now added:

"Energy-dispersive X-ray spectroscopy (EDS) measurements at nine spatially distributed positions showed good compositional uniformity in both films (Supplementary Fig. 6)."

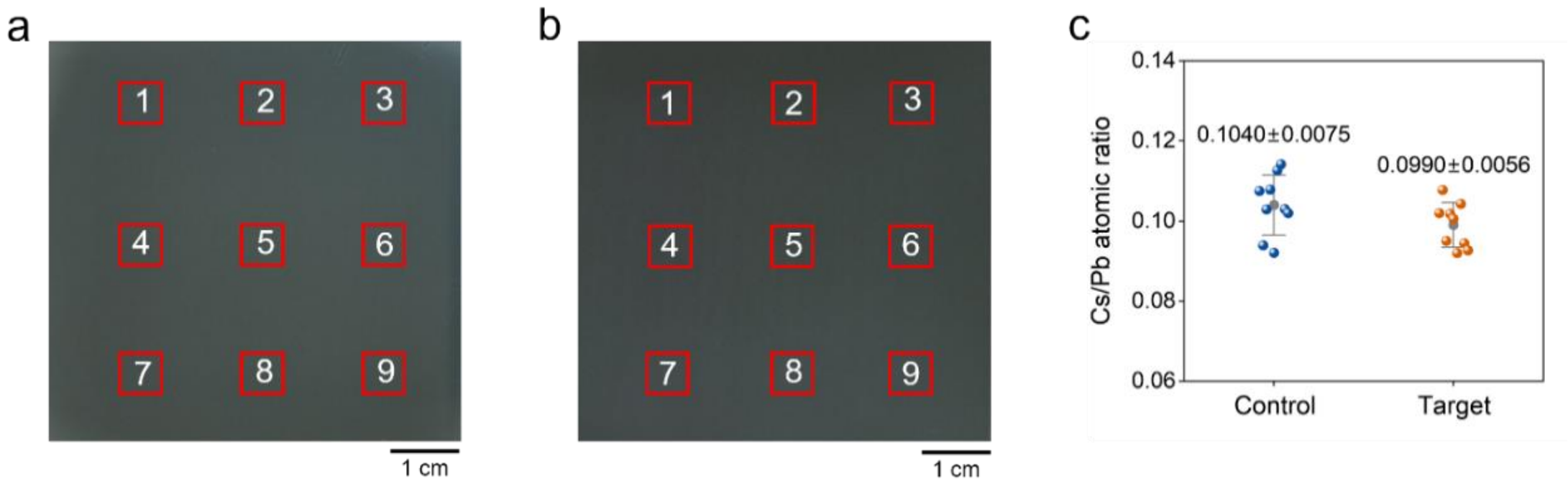

Supplementary Fig. 6 | Spatial compositional uniformity of control and target perovskite films determined by energy-dispersive X-ray spectroscopy (EDS). a, b, Photographs of the control (**a**) and target (**b**) perovskite films showing the nine spatially distributed positions selected for EDS measurements. **c,** Cs/Pb atomic ratios obtained at the corresponding positions. Individual points represent measurements at different spatial locations, and grey symbols and error bars indicate the mean ± s.d. (N = 9 spatial positions).

5. Please provide cross-sectional SEM images of the control and target devices. These images would allow comparison of the through-thickness grain structure, grain

boundaries, void formation, layer thicknesses and interfacial contact quality. Such information may also help explain the observed improvement in FF after cryo-controlled processing.

Response: We thank the reviewer for this helpful suggestion. Cross-sectional SEM images of the control and target devices have now been added in Supplementary Fig. 4. The target device shows larger grains extending through much of the perovskite-layer thickness, with fewer apparent through-thickness grain boundaries and no obvious void formation, indicating improved morphological quality of the perovskite layer. Accordingly, in the "Recombination-loss suppression" section, page 6, second paragraph, the revised text now reads:

"XRD patterns showed sharper diffraction peaks in the target films than in the control films (Fig. 2f), while top-view SEM images revealed larger and more uniform grains (Fig. 2g), and cross-sectional SEM images showed larger grains extending through much of the perovskite-layer thickness (Supplementary Fig. 4), together indicating improved crystallinity and morphological quality."

a

b

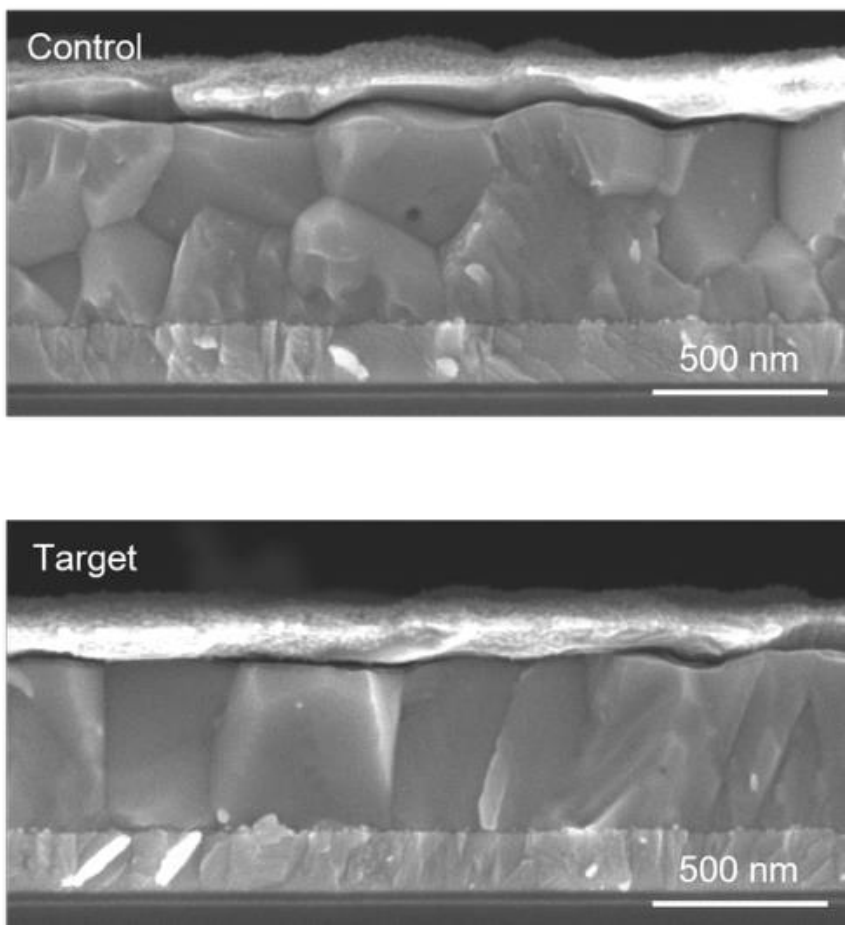

Supplementary Fig. 4 | Cross-sectional scanning electron microscopy (SEM) images of perovskite devices. a, Control perovskite device. **b,** Target perovskite device. Scale bars, 500 nm.

6. In Supplementary Fig. 9, the target device exhibits higher Voc and FF but a slightly lower Jsc than the control device. However, the EQE spectra do not indicate an obvious change in the absorption onset or band edge (I notice that EQE is from minimodule). Could the authors explain the origin of the reduced Jsc?

Response: We thank the reviewer for raising this question. The control and target devices shown in the original Supplementary Fig. 9 (now Supplementary Fig. 12) are the respective champion devices, with J_{sc} values of 25.2 and 24.8 mA cm^{-2}, respectively (Supplementary Table 4). To determine whether this difference represents a systematic effect of cryo-controlled processing, we have now added device statistics for ten 0.09 cm^2 control and target devices in Supplementary Table 5, as shown below. The average J_{sc} values are fairly close, at 24.95 and 25.05 mA cm^{-2} for the control and target devices, respectively.

Supplementary Table 5 | Photovoltaic performance of 0.09 cm^2 control and target perovskite cells. Values are presented as mean ± s.d. for 10 devices measured under AM1.5G illumination (100 mW cm^{-2}).

	V_{OC} (V)	J_{SC} (mA cm^{-2})	*FF* (%)	PCE (%)
Control	1.197 ± 0.014	24.95 ± 0.26	83.22 ± 0.89	24.8 ± 0.22
Target	1.220 ± 0.013	25.05 ± 0.23	83.64 ± 0.77	25.6 ± 0.28

Consistently, the control and target mini-modules show closely overlapping EQE spectra (Supplementary Fig. 10), with EQE-integrated current densities of 24.14 and 24.22 mA cm^{-2}, respectively. These results show that cryo-controlled processing does not systematically reduce J_{sc}, indicating that the slightly lower J_{sc} of the individual target device in Supplementary Fig. 12 falls within the normal range of device-to-device variation.

We hope that the revised manuscript is now in a form suitable for publication in Nature Communications.

Respectfully,
The Authors

Response to Reviewers' Comments

We thank the Editor and the Reviewers for their support of our manuscript and for their constructive comments and suggestions, which have substantially improved the manuscript. We have now carefully addressed the remaining comments and revised the manuscript accordingly.

REVIEWER COMMENTS:

Reviewer #1:

The authors have made a genuine effort to address my concerns, particularly by reframing the innovation narrative from cryo-controlled processing to FF preservation as a strategy for mitigating efficiency loss and by providing a quantitative analysis of FF losses. However, FF preservation for mitigating efficiency loss is an opinion rather than a novelty or a genuine breakthrough in mechanism or methodology. I suggest the authors to rigorously differentiate their approach from prior art not just in terminology, but in demonstrable mechanistic distinctions.

Response: We thank the reviewer for suggesting that we further highlight the mechanistic distinctions of our work. We directly quantify the practical cell-to-module efficiency loss, establishing FF as a major contributor, and further resolve the parameter-level factors underlying FF preservation. In our cryo-controlled demonstration, the FF-preservation advantage arises predominantly from reduced diode non-ideality and increases with device scaling. This quantitative connection, also noted by Reviewer 2, remains relatively underexplored in high-performance perovskite photovoltaics.

Accordingly, we have revised the Abstract, Introduction and Discussion to reflect this reframing and make the mechanistic distinctions clearer. For example, the Abstract now states:

"Further analysis resolves the FF deficit into voltage-, diode-non-ideality- and transport-associated contributions. … The resulting FF-preservation advantage arises predominantly from reduced diode non-ideality, with the other contributions being smaller."

Reviewer #2:

Thank you for the authors' careful and thorough responses. My previous comments have been well addressed, and I believe the manuscript has been substantially improved. In particular, the revised manuscript establishes a clearer and more convincing link between the processing strategy, the ideality factor, and FF preservation. Such a quantitative connection remains relatively underexplored, particularly in high performance perovskite photovoltaic devices. I therefore recommend acceptance of the manuscript in its present form.

Response: We sincerely thank the reviewer for the positive assessment of our revised manuscript and for the constructive comments throughout the review process.

Reviewer #3:
The authors have adequately addressed the concerns raised in my previous review. The revisions and additional clarifications have satisfactorily resolved the major issues, and the manuscript has been further improved. I therefore recommend the manuscript for publication in Nature Communications.
Response: We sincerely thank the reviewer for the positive assessment of our revised manuscript and for the constructive comments throughout the review process.

Respectfully,
The Authors
